# First Description of a Satellite DNA in Manatees’ Centromeric Regions

**DOI:** 10.3389/fgene.2021.694866

**Published:** 2021-08-24

**Authors:** Mirela Pelizaro Valeri, Guilherme Borges Dias, Alice Alves do Espírito Santo, Camila Nascimento Moreira, Yatiyo Yonenaga-Yassuda, Iara Braga Sommer, Gustavo C. S. Kuhn, Marta Svartman

**Affiliations:** ^1^Laboratório de Citogenômica Evolutiva, Departamento de Genética, Ecologia e Evolução, Instituto de Ciências Biológicas, Universidade Federal de Minas Gerais, Belo Horizonte, Brazil; ^2^Department of Genetics and Institute of Bioinformatics, University of Georgia, Athens, GA, United States; ^3^Departamento de Genética e Biologia Evolutiva, Instituto de Biociências, Universidade de São Paulo, São Paulo, Brazil; ^4^Centro Nacional de Pesquisa e Conservação da Biodiversidade Marinha do Nordeste, Instituto Chico Mendes de Conservação da Biodiversidade, Brasília, Brazil

**Keywords:** tandem repeats, *Trichechus manatus*, *Trichechus inunguis*, chromosome mapping, fluorescent *in situ* hybridization, TAREAN

## Abstract

*Trichechus manatus* and *Trichechus inunguis* are the two Sirenia species that occur in the Americas. Despite their increasing extinction risk, many aspects of their biology remain understudied, including the repetitive DNA fraction of their genomes. Here we used the sequenced genome of *T. manatus* and TAREAN to identify satellite DNAs (satDNAs) in this species. We report the first description of TMAsat, a satDNA comprising ~0.87% of the genome, with ~684bp monomers and centromeric localization. In *T. inunguis*, TMAsat showed similar monomer length, chromosome localization and conserved CENP-B box-like motifs as in *T. manatus*. We also detected this satDNA in the *Dugong dugon* and in the now extinct *Hydrodamalis gigas* genomes. The neighbor-joining tree shows that TMAsat sequences from *T. manatus*, *T. inunguis*, *D. dugon*, and *H. gigas* lack species-specific clusters, which disagrees with the predictions of concerted evolution. We detected a divergent TMAsat-like homologous sequence in elephants and hyraxes, but not in other mammals, suggesting this sequence was already present in the common ancestor of Paenungulata, and later became a satDNA in the Sirenians. This is the first description of a centromeric satDNA in manatees and will facilitate the inclusion of Sirenia in future studies of centromeres and satDNA biology.

## Introduction

The order Sirenia encompasses four extant herbivorous aquatic mammals. The Dugongidae family includes the *Dugong dugon* and the Steller’s sea cow *Hydrodamalis gigas*, the latter now extinct due to overhunting, and the Trichechidae family includes three manatee species: *Trichechus manatus*, *Trichechus inunguis*, and *Trichechus senegalensis* (Domning, 2018). *Dugong dugon* occurs across coastal waters in the Indo-West Pacific Ocean, and *T. senegalensis* is restricted to the west coast of Africa, making *T. manatus*, the West Indian manatee, and *T. inunguis*, the Amazonian manatee, the only sirenians to occur in the Americas. The West Indian manatee occurs in Caribbean waters and the Atlantic coast ranging from Florida to the northeast of Brazil, and *T. inunguis* is found along the Amazon River basin (Bonvicino et al., 2020). All extant sirenians are considered as vulnerable by the International Union for Conservation of Nature and Natural Resources (IUCN; Deutsch et al., 2008; Keith, 2015; Marmontel et al., 2016; Marsh and Sobtzick, 2019).

The West Indian manatee has two recognized subspecies: *Trichechus manatus latirostris* (Florida Manatee), found in the United Estates and Gulf of Mexico coasts, and *T. m. manatus* (Antillean manatee), found in the Caribbean, Central and South America. Recent morphological and genetic analyses suggest the need for a revision in the *T. manatus* taxonomy considering the influence of the Amazon River as a barrier to gene flow. These studies showed that the *T. m. manatus* populations from the Caribbean and up to the Amazon River mouth are phylogenetically closer to the populations of *T. m. latirostris* from the United States than to the Brazilian *T. m. manatus* populations south of the Amazon River mouth (Vianna et al., 2006; Barros et al., 2017; Lima et al., 2019, 2021). Hybrids between *T. manatus* and *T. inunguis* have also been reported on the sympatric area at the Amazon River mouth (Vianna et al., 2006; Lima et al., 2019; Luna et al., 2021).

Satellite DNAs (satDNAs) are a type of repetitive DNA found in most eukaryotic genomes. They are arranged as long arrays of tandem repeats with variable unit length, number of copies and chromosome organization. SatDNAs are usually associated with chromosome landmarks such as centromeres, telomeres, and heterochromatic regions. Despite the fact that satDNAs do not encode proteins, they are associated with important biological functions such as formation and maintenance of heterochromatin at telomeres and centromeres, and maintenance of chromosome integrity and genome stability (reviewed in Shapiro and von Sternberg, 2005; Biscotti et al., 2015; Shatskikh et al., 2020). SatDNAs can form higher-order repeat (HOR) units made of multimers with a number of diverged monomers that are tandemly repeated as a set (reviewed in Plohl et al., 2012; Vlahović et al., 2016). HOR organization has been found in several satDNAs, including the alfa centromeric satDNA in humans, and may be relevant to the centromeric function (Sujiwattanarat et al., 2015; Sullivan et al., 2017). In addition, satDNAs monomer sequences can present internal repetitions, which may be related with secondary structures relevant to centromeric function (Kasinathan and Henikoff, 2018). Centromeric satDNAs in mammals usually present the CENP-B box, a conserved 17bp region known to be the DNA-binding domain for the centromeric protein B (CENPB), with nine nucleotides (nTTCGnnnnAnnCGGGn) composing the most evolutionarily conserved domain (ECD; Muro et al., 1992; Masumoto et al., 2004; Alkan et al., 2011; Kasinathan and Henikoff, 2018). Most satDNAs are under concerted evolution, a process by which new mutations within monomers are quickly homogenized across the repeat family and fixed in reproductively isolated populations, resulting in intraspecific repeat homogeneity but interspecific divergence (Dover, 1982; Plohl et al., 2012; Smalec et al., 2019). Moreover, according to the library model, related species may share a collection of satDNAs sequences with mostly quantitative interspecies differences due to expansion or contraction (even elimination) during the evolution (Fry and Salser, 1977; Meštrović et al., 1998). Another aspect of satDNAs evolution is their relationship with mobile elements, since there are several examples of satDNAs derived from transposons and retrotransposons in plants and animals (reviewed in Meštrović et al., 2015).

The repetitive DNA fraction of manatees’ genomes has been poorly studied, especially in the case of satDNAs. We used the sequenced genome of *T. manatus* and the TAREAN (Novák et al., 2017, 2020) pipeline to explore the satDNAs present in this genome. Herein, we describe for the first time the centromeric satDNA of the West Indian manatee, which we found to be also present in the Amazonian manatee, the dugong, and in the extinct Steller’s sea cow. We characterized this sequence *in silico* and mapped it in *T. manatus* and *T. inunguis* chromosomes. In addition, we investigated the presence of the TMAsat sequence in mammals outside the order Sirenia, which allowed us to establish a rough timeline for its origin.

## Materials and Methods

### *De novo* Identification of Satellite DNAs

In order to identify satDNAs in manatees, we used whole-genome sequencing data from *T. m. latirostris* (accession number SRR328416) available in the *National Center for Biotechnology Information* – NCBI and the TAREAN pipeline (Novák et al., 2017). The first step of this pipeline is a graph-based clustering, which performs all to all similarity comparisons of DNA sequencing reads, resulting in clusters of those reads derived from repetitive elements. Then, it examines the presence of circular or globula-like graph structures to identify potential tandem repeats, classified as putative high or low confidence satellites. The raw Illumina reads (~100bp long) used in this analysis were randomly sampled by TAREAN, comprising ~2.4% (870,965 reads) of the 3.67pg estimated genome size (Kasai et al., 2013). The reads that make up each cluster are partially assembled into contigs that were used for repeat annotation with the CENSOR web server (Kohany et al., 2006) that contains a collection of Mammalia repeats from RepBase, updated in 08-24-2020 (Bao et al., 2015). The single potential tandem repeat cluster (13) with globula/ring-like structure was analyzed in detail through similarity searches against the *T. manatus* reference genome (accession GCA_000243295.1) using the BLASTn tool with default parameters (Altschul et al., 1990) to verify if the sequence is a tandem repeat. In addition to the annotation using the CENSOR web server, this cluster was annotated through BLASTn similarity searches against the whole nucleotide collection (nr/nt).

The identified satDNA sequence was characterized regarding its genome proportion, monomer length, AT content, and presence of internal direct or inverted duplications. The satDNA genome proportion was estimated by TAREAN. TAREAN tries to improve the assembly process by applying a k-mer-based approach to obtain a less fragmented monomer consensus, but it restricts itself to the 50% most prevalent k-mers in a cluster. For this reason, we chose the whole-genome assembly resource as a more representative sample of TMAsat diversity. The most common sequence (MCS) of TMAsat was generated using Geneious (prime version 2020.2.4) with a 25% threshold and 66 monomeric sequences retrieved from the reference genome, previously aligned with the muscle aligner implemented in MEGA X. The MCS was used to estimate monomer length, AT content, and presence of internal repetitions. The last feature was also conducted in the Geneious software using the diagonal plot method in high sensitive mode, with window size of 50bp and identity threshold of 60%.

We searched for the presence of TMAsat in the two other Sirenia species with a sequenced genome available in NCBI, *D. dugon* (under accession numbers of assembled genome GCA_015147995.1 and raw Illumina reads DRR251525) and the extinct *H. gigas* (under accession numbers of assembled genome GCA_013391785.1 and raw Illumina reads SRR12067498). First, we used TMAsat sequence as query in BLASTn similarity searches against these assembled genomes. In addition, we also used the raw Illumina reads (~150bp long) and TAREAN to identify TMAsat in these genomes. The analyzed reads were randomly sampled by TAREAN totalizing 1,038,927 in *D. dugon* and 570,097 in *H. gigas*. The MCS of TMAsat in *D. dugon* and *H. gigas* were generated using monomeric sequences retrieved from the reference genome after BLAST searches, totalizing 50 sequences from *D. dugon* and 40 from *H. gigas*. The TMAsat MCS in *T. inunguis* was obtained using the five cloned sequences obtained by PCR. All MCS were generated as described previously for *T. manatus*.

### Biological Samples

We used biological samples of *T. manatus* and *T. inunguis* to determine TMAsat chromosomal distribution and investigate its presence in *T. inunguis*, whose genome has not been sequenced. Skin sample from a male *T. manatus* captured at Porto de Pedras/AL, Brazil (−9.164167 and −35.294444) in 2019 was provided by CEPENE/ICMBio (SISBIO 60829-2) and used for fibroblast culture. A fibroblast cell line from a male *T. inunguis* established in 1998 was provided by Dr. Yatiyo Yonenaga-Yassuda, from the University of São Paulo, and was previously analyzed by Assis et al. (1998). Chromosome spreads from fibroblast cultures were obtained according to Stanyon and Galleni (1991) and genomic DNAs were extracted with the Wizard Genomic DNA Purification Kit (Promega).

### PCR Amplification, Cloning, and Sequencing of Satellite DNAs

TMAsat was amplified by PCR from the *T. inunguis* genomic DNA using primers designed from the satDNA consensus sequence (estimated by TAREAN) as follow: TMAsat-F CTCCTTCAAGCTGCTTAACT and TMAsat-R GGGAACTTACACTTGCTGCT. The PCR cycling conditions were as follows: 95°C – 3min, 35cycles: 95°C – 30s; 55°C for 30s; 72°C – 1min; and 72°C – 3min for final elongation. The PCR product corresponding to monomer size was excised from the agarose gel and purified using the Illustra GFX PCR DNA and Gel Band Purification Kit. The purified products were ligated into the pGEM-T Easy vector (Promega) and used in the transformation of *Escherichia coli* XL1-BLUE strain electrocompetent cells (Phoneutria). Five recombinant colonies of TMAsat were sequenced (access numbers MW272776–MW272780) with the ABI3130 platform (Applied Biosystems).

### Fluorescence *in situ* Hybridization

Fluorescent *in situ* hybridization (FISH) was performed using the TMAsat cloned (MW272776) sequence as probe on metaphase spreads of *T. manatus* and *T. inunguis*. FISH was performed with 200ng of biotin-labelled probes, following (Valeri et al., 2020). The analyses and image acquisition were performed under a Zeiss Axioimager 2 epifluorescence microscope equipped with a CCD camera and with the AxioVision software (Carl Zeiss MicroImaging, Jena, Germany), respectively.

### *In silico* Characterization of satDNAs

DNA polymorphisms and nucleotide diversity along the satDNA sequences were analyzed using the software DnaSP 6.12.03 (Rozas et al., 2017) with the same monomer sequences used to generate the MCS from *T. manatus*, *D. dugon*, and *H. gigas*. In this analysis, the monomer sequences were previously aligned with the muscle method (Edgar, 2004) implemented in MEGA X and the window length and step size were set for 10 and 1bp, respectively. Windows were classified as conserved or variable if they exhibited more than two SDs bellow or above the nucleotide average variability, respectively.

Monomer sequences of TMAsat from *T. manatus*, *T. inunguis*, *D. dugon*, and *H. gigas* were aligned with the muscle method implemented in MEGAX and used for the construction of a neighbor-joining tree. These sequences were the same used to obtain the MCS, totalizing 161 sequences, including 66 from *T. manatus*, five from *T. inunguis*, 50 from *D. dugon* and 40 from *H. gigas*. The neighbor-joining tree was obtained using MEGA X with 500 bootstrap replicates and the final tree was visualized in iTOL v4.3.3^1^ (Letunic and Bork, 2019). We also used the same set of sequences to estimate the inter- and intra-specific nucleotide divergence (number of base substitutions per site), as well as the average nucleotide divergence over all pairwise sequence comparisons using MEGA X.

We searched for any putative CENP-B box in TMAsat MCS from *T. manatus*, *T. inunguis*, *D. dugon*, and *H. gigas* using the 17bp sequence containing the ECD (nTTCGnnnnAnnCGGGn; Masumoto et al., 2004) and CENP-B box sequences of *Loxodonta africana*/*Dasypus novemcintus* (CTTTGCCGAGAACGGAG; Alkan et al., 2011). This search was conducted in the Geneious software in global pairwise alignment mode and 51% similarity cost matrix.

To investigate the presence of TMAsat in other mammals, we utilized the MCS from *T. manatus* as query in BLASTn similarity searches against Mammalia (NCBI:txid40674) wgs database excluding Sirenia (NCBI:txid9774; search date 06-07-2021). The flanking regions of TMAsat similarity hits were analyzed with the CENSOR web server (Kohany et al., 2006) containing the Mammalia RepBase library (updated in 06-14-2021; Bao et al., 2015). To better analyze these hits with the TMAsat consensus sequence, we compared their sequences using dotplots and pairwise alignments in the Geneious software.

## Results

### *In silico* Identification and satDNA Analyses

The only potential satDNA identified (with low confidence) by TAREAN in the *T. manatus* genome was represented by the cluster 13. This sequence was analyzed in detail through similarity searches against the *T. manatus* reference genome (accession GCA_000243295.1) using the BLASTn tool with default parameters (Altschul et al., 1990). Despite being classified by TAREAN with low confidence, we verified this sequence tandemly repeated at least 25 times on assembled contigs of *T. manatus*. These repeats comprise 0.87% of the genome of *T. manatus* with monomer length of ~684bp estimated by TAREAN. The consensus sequence generated by TAREAN (Supplementary Figure 1) did not show similarity with any known repetitive DNA from the mammalian RepBase collection (Bao et al., 2015). We named this new satDNA as TMAsat (for *T. manatus* satellite).

**Figure 1:**
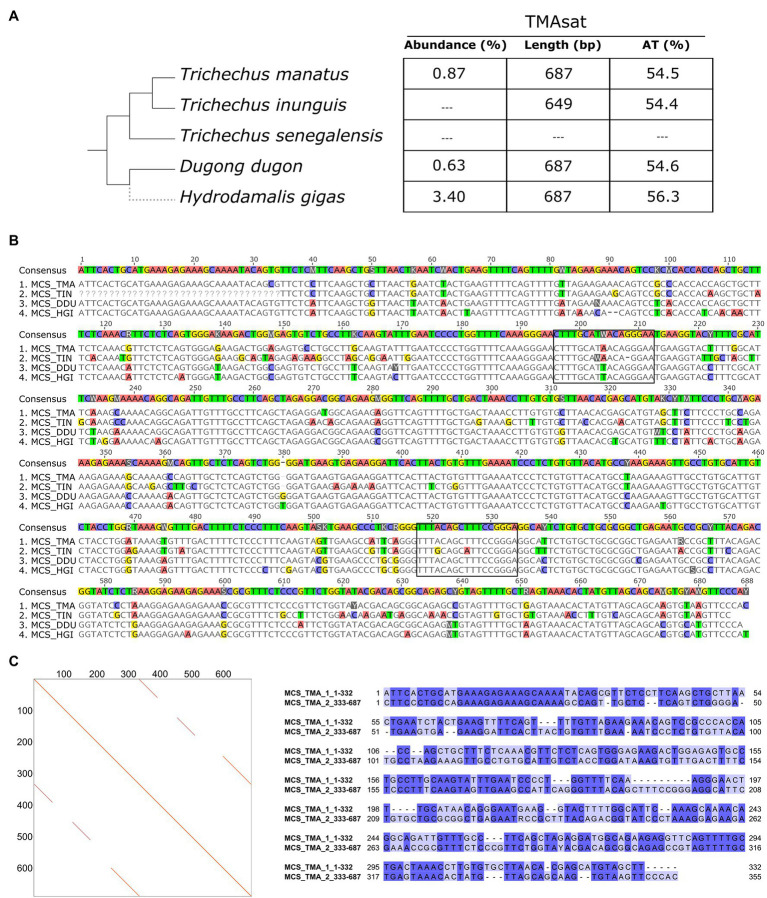
**(A)** TMAsat abundance, monomer length and AT content of the monomers for the analyzed species. **(B)** Alignment of TMAsat MCS from *Trichechus manatus*, *Trichechus inunguis*, *Dugong dugon*, and *Hydrodamalis gigas* showing the two putative CENP-B box like motifs. **(C)** Dot plot comparison of the TMAsat MCS sequence from *T. manatus* against itself and pairwise alignment of TMAsat position 1–332 against 333–687, with 55.8% of DNA sequence identity.

The TMAsat MCS from *T. manatus* was generated from an alignment of 66 monomers manually isolated from the assembled reference genome (Supplementary Figure 2; Supplementary File 1). It showed monomer length of 687bp and 54.5% of AT content (Figures 1A,B). The dotplot of TMAsat against itself revealed a segment repeated twice inside TMAsat, from position 1 to 332 and 333 to 687 (Figure 1C), A pairwise alignment of the two segments of TMAsat, 1–332 and 333–687bp, showed that they are related but quite divergent, with only 55.8% identity (Figure 1C). A detailed investigation in the assembled contigs showed that the TMAsat unit of ~687bp is organized in higher-order structure, mostly alternating the segments TMAsat1 (1–332) and TMAsat2 (333–687). However, we found one case of TMAsat1 dimer (accession NW_004443969.1 position 56,989–75,023bp), few cases of TMAsat2 dimer (accessions NW_004444053.1; NW_004444936.1; and NW_004444425.1) and in one contig (accession NW_004443969.1) three, six and 10 tandemly repeated units of TMAsat2.

### Genomic Distribution of TMAsat in the Genus *Trichechus*

**Figure 2:**
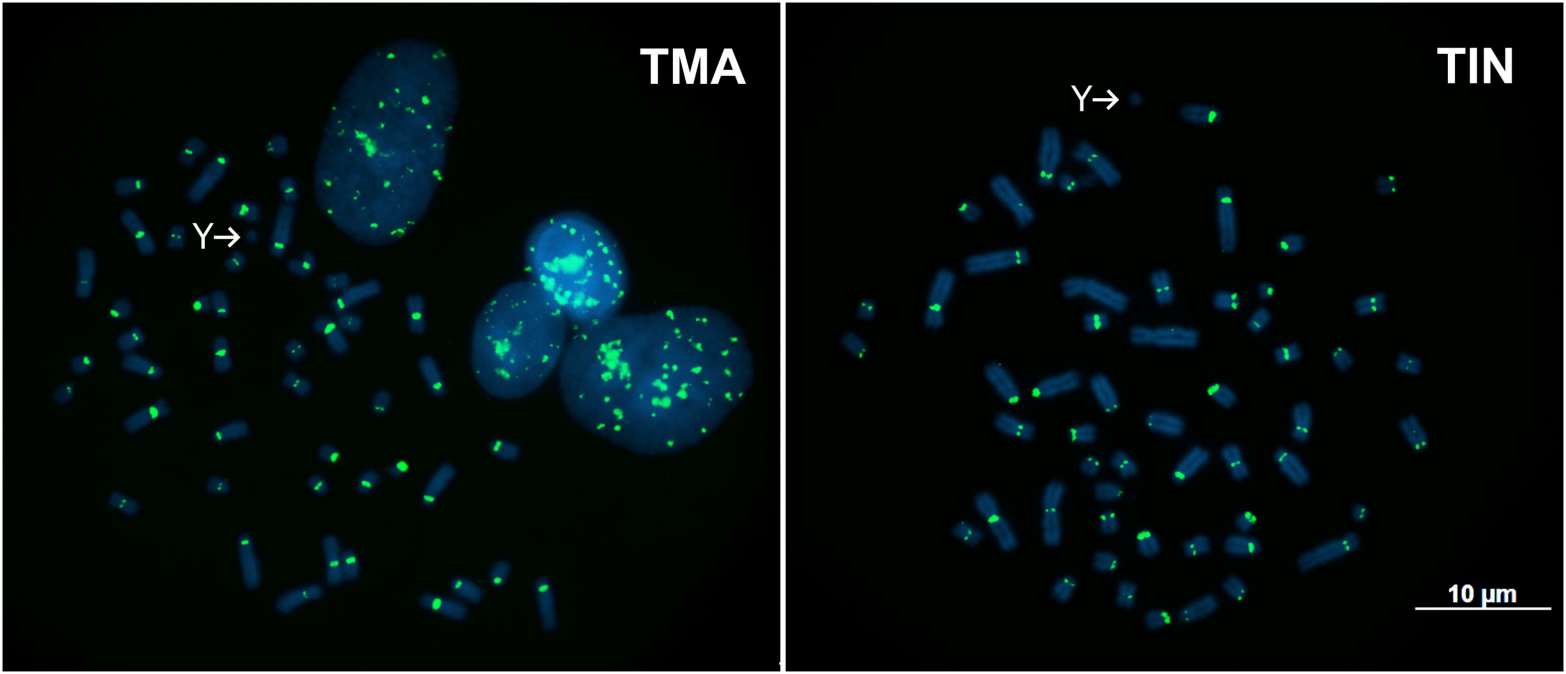
Metaphases of *T. manatus* (TMA) and *T. inunguis* (TIN) after FISH with the TMAsat probe. Y chromosomes without signals of TMAsat are indicated.

TMAsat was amplified by PCR from *T. inunguis* genomic DNA, and the resulting PCR products showed a similar monomer length of ~647bp (Supplementary Figure 3). The PCR product was cloned and sequenced in order to confirm that it was indeed homologous to TMAsat. The MCS based on the cloned sequences showed similar AT content and 89.6% of identity (Figures 1A,B; Supplementary Figure 4). A selected TMAsat cloned sequence was labeled and used as probe in FISH on chromosomes of both *T. manatus* and *T. inunguis*. TMAsat showed centromeric localization in *T. manatus* (2*n*=48) and *T. inunguis* (2*n*=56), mapping to the centromeres of all chromosomes, except the Y (Figure 2; Supplementary Figure 5). TMAsat localization is compatible with the CBG-banding pattern in both species, which reveals centromeric heterochromatin in all chromosomes (Assis et al., 1998; Gray et al., 2002), with the exception of the Y.

### TMAsat in Other Sirenia

Besides *T. manatus*, there are two additional Sirenia species with sequenced genomes available: *D. dugon* and the extinct *H. gigas*, both belonging to the Dugongidae family. A search for TMAsat sequences on the assembled contigs of these species revealed the presence of tandemly repeated TMAsat sequences. TAREAN returned with high confidence one cluster of a putative satDNA with 685bp length in both species, cluster 8 in *D. dugon* and cluster 3 in *H. gigas*, which contained homologous sequences to TMAsat (Figures 1A,B). In *D. dugon*, cluster 8 represents 0.63% of the genome and the MCS generated from the 50 monomers retrieved from the assembled genome is 687bp long with 54.6% of AT content (Supplementary Figure 6; Supplementary File 2). In addition, we found evidence of other HOR configurations rather than alternating TMAsat1 and TMAsat2 in *D. dugon*: a dimer of TMAsat1 (BMBL01107524.1 and BMBL01079760.1), a dimer of TMAsat2 (BMBL01112453.1 and BMBL01093845.1), four (BMBL01013125.1), five (BMBL01107524.1) and six (BMBL01055248.1) tandemly repeated copies of TMAsat2.

In *H. gigas*, cluster 3 comprises 3.4% of the genome and the MCS based on 40 monomers from the reference genome is 687bp long and has 56.3% of AT content (Supplementary Figure 7; Supplementary File 3). In this species, the most frequent TMAsat organization is the alternating segments of TMAsat1 and TMAsat2, and we only found one dimer of TMAsat2 (JACANZ010402190.1).

**Figure 3:**
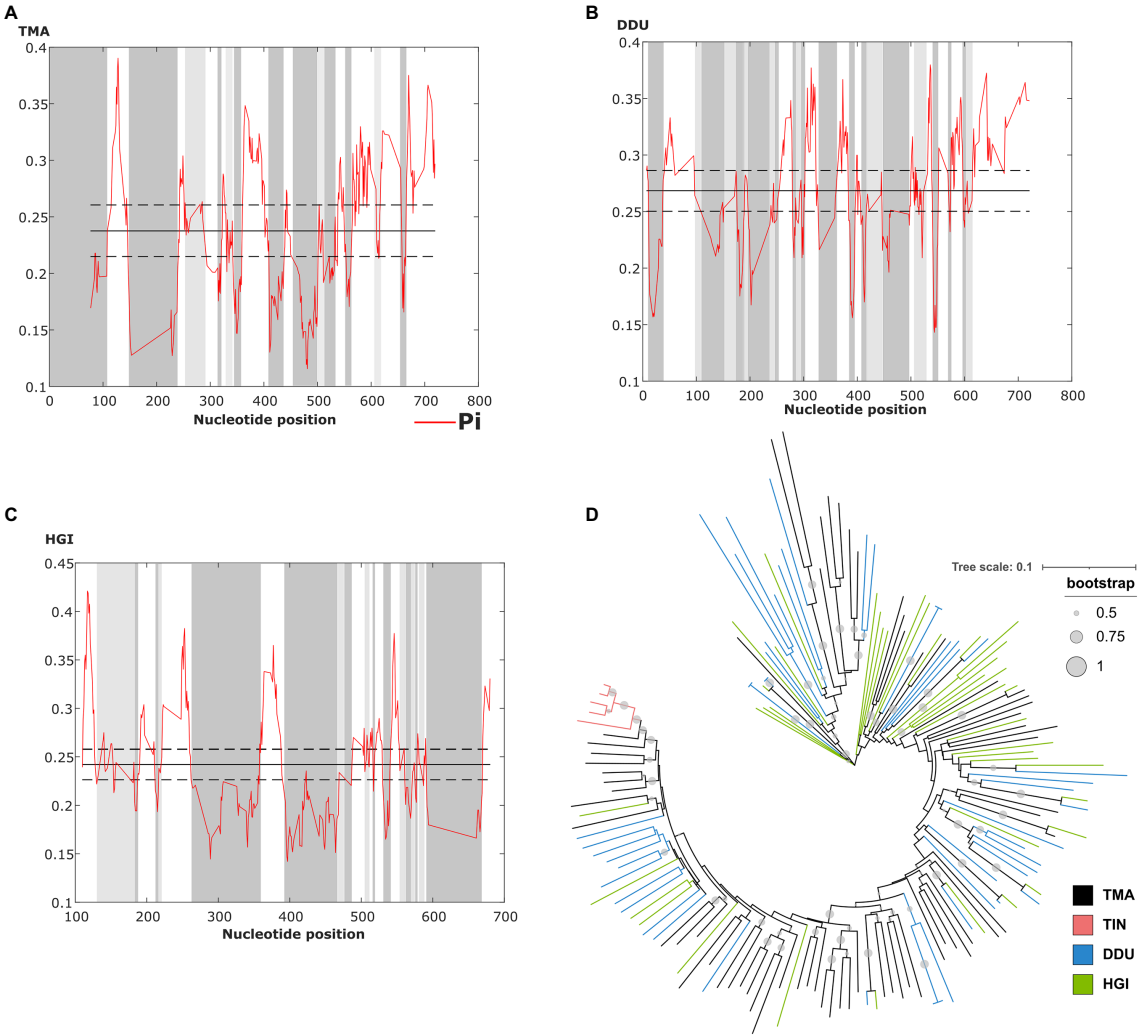
Identification of conserved and variable TMAsat segments of **(A)** *T. manatus*, **(B)** *D. dugon* and **(C)** *H. gigas* by sliding window analysis (sliding window of 10bp and step size of 1bp). Average nucleotide diversity (Pi) is indicated by the red line, while average nucleotide diversity is indicated by the solid line, and average diversity ± 2 SD is indicated by the dashed line. **(D)** Neighbor-joining tree with TMAsat sequences of *T. manatus* (TMA), *T. inunguis* (TIN), *D. dugon* (DDU), and *H. gigas* (HGI). Bootstrap values generated from 500 replicates.

The sliding window analysis of nucleotide variability of this satDNA in *T. manatus*, *D. dugon*, and *H. gigas* revealed the presence of conserved and variable regions within the monomers (Figures 3A–C). However, we did not have access to biological samples of *D. dugon* or *H. gigas* to map TMAsat on their chromosomes. The monomeric TMAsat sequences from *T. manatus*, *T. inunguis*, *D. dugon*, and *H. gigas* were aligned and used to construct a neighbor-joining tree, which did not reveal any species-specific clustering (Figure 3D). We also estimated the inter and intraspecific nucleotide divergence (Supplementary Table 1), as well as the average divergence over all sequence pairs (*d*=0.34). As expected from the Neighbor Joining results, TMAsat intraspecific diversity was not lower than interspecific diversity, except in *T. inunguis* (*d*=0.06). The low diversity of TMAsat sequences in *T. inunguis* may be due to the low number of sequences used in the analysis and the use of PCR.

### CENP-B Box Is Present in TMAsat

The CENP-B box is a 17bp region conserved among mammalian centromeric satDNAs and known to be the DNA-binding domain for the centromeric protein CENPB. We searched for putative CENP-B box-like motifs within TMAsat MCS from *T. manatus*, *T. inunguis*, *D. dugon*, and *H. gigas*, and found two putative motifs MCS (Figure 1B). The first is located in position 196 to 212bp, matching best with the CENP-B box sequence found in *L. africana* and *D. novemcinctus*. The second putative motif was found in position 518–534bp. Both motifs display 5–6 identical nucleotides to ECD out of nine in all Sirenia species (Table 1). The two putative CENP-B box motifs were present in a conserved segment as indicated by the sliding window analysis of nucleotide variability among satDNA monomers from *T. manatus* and *D. dugon* (Figures 3A,B). In *H. gigas*, these motifs spanned both conserved and variable regions of the TMAsat monomer (Figure 3C).

**Table 1:** The two putative CENPB box-like motifs identified in the MCS of TMAsat from *T. manatus* (TMA), *T. inunguis* (TIN), *D. dugong* (DDU), and *H. gigas* (HGI).

	Position 196–212bp	Position 518–534bp
ECD	NTTCGNNNNANNCGGGN	NTTCGNNNNANNCGGGN
*L. africana* and *D. novemcinctus*	CTTTGCCGAGAACGGAG	CTTTGCCGAGAACGGAG
*T. manatus*	CTTTGCATAACAGGGAA	TTTACAGCTTTCCGGGA
*T. inunguis*	CTTTGCAWAACAGGAAT	TTTGCAGCATTCCGGGA
*D. dugon*	CTTTGCATTACAGGGAA	TTTACAGCTTTCCGGGA
*H. gigas*	CTTTGCATTACAGGGAA	TTTACAGCTTTCCGGGA

Conserved nucleotides in the evolutionarily conserved domain (ECD) are shown in red/highlighted, and conserved nucleotides compared with *L. africana* and *D. novemcinctus* motif (Alkan et al., 2007) other than the ECD domain are shown in blue.

The CENP-B box-like motifs found in positions 196–212bp of TMAsat from *T. manatus* (CTTTGCATAACAGGGAA) and *T. inunguis* (CTTTGCAWAACA-GGAAT) shared 14 out of the 17 nucleotides with each other. In *D. dugon* and in *H. gigas* the CENPB-box-like motif was the same (CTTTGCATTACAGGGAA) and shared 15 out of 17 nucleotides with *T. manatus*. Six bases in *T. manatus*, *D. dugon* and *H. gigas* and five in *T. inunguis*, out of the nine from the ECD were conserved. The second putative motif (position 518–534bp) showed six out of nine identical bases to the ECD in the four analyzed species. *T. manatus*, *D. dugon*, and *H. gigas* shared an identical second motif (TTTACAGCTTTCCGGGA), whereas *T. inunguis* differed in two nucleotides (TTTGCAGCATTCCGGGA).

### TMAsat in Other Mammals

We investigated the presence of TMAsat in other mammals using the MCS from *T. manatus* as query in similarity searches against Mammalia sequences in the wgs database from NCBI excluding Sirenia. The total number of returned hits was 13 distributed in four species (Supplementary Table 2). With a cut off for query cover equal or greater than 30%, we found four hits in the African elephant (*L. africana*) and four hits in the Asian elephant (*Elephas maximus*). In addition to African and Asian elephants, *Procavia capensis* and *Heterohyrax brucei* appeared in the hits with query covers smaller than 30%. Looking closer into these contigs from *L. africana*, *E. maximus*, *Procavia capensis*, and *Heterohyrax brucei*, we verified few sequences in tandem (maximum of 18) with the repetition unit comprising roughly one TMAsat HOR monomer. The small number of hits found suggests that this sequence is not a typical satDNA in these taxa, but is instead a repetition related to a transposable element. Indeed, nine out of 10 TMAsat arrays were flanked by LINE-1 in *L. africana*, and 15 out of 16 in *E. maximus*.

## Discussion

The TMAsat, reported herein for the first time, was the only putative satDNA found in our analysis, comprising less than 1% of the *T. manatus* genome and mapping to the centromeric regions of all chromosomes, except the Y. The TMAsat could be absent or undetectable by FISH due to low copy number or sequence divergence on the Y chromosome. In *T. inunguis*, we confirmed the presence of TMAsat by PCR and FISH and despite the two species having different karyotypes (2*n*=48 and 2*n*=56, respectively), TMAsat displayed the same chromosome localization (Figure 2). This could be related to the recent ~1.34 million years ago (Mya) divergence time between the species (de Souza et al., 2021).

We also detected the TMAsat in *D. dugon* and *H. gigas* with similar monomer length, comprising 0.63 and 3.4% of the genomes, respectively. The different genome proportion found in *T. manatus* (Illumina HiSeq; 150x genome coverage), *D. dugon* (Illumina Novaseq6000; 64x genome coverage) and *H. gigas* (Illumina NovaSeq; 11x genome coverage) could be due to different genome coverage and/or sequencing platforms used for each species, and may not reflect real interspecific variation. This is especially true in the case of the extinct *H. gigas*, whose DNA source for genome sequencing is a petrous bone from a specimen who probably died during the 1760s (Sharko et al., 2021), and thus the abundance estimates need to be taken with caution.

Although there are slight differences within the MCS from each species, the Neighbor Joining analysis does not indicate intraspecific homogeneous monomers. Only the monomers from *T. inunguis* were grouped together, probably due to the low number of sequences used in the analysis or biased PCR amplification with the selected primers. Nevertheless, we cannot discard a species-specific TMAsat sequence in *T. inunguis* since some mutations are present in all or almost all five sequences and are absent or present in just few monomers outside the species. West Indian and Amazonian manatees present a recent divergence time (de Souza et al., 2021) and an incomplete reproductive isolation (Vianna et al., 2006; Lima et al., 2019), which could contribute to the TMAsat high interspecific homogeneity observed. Overall, the species-specific mutations of the group are probably not yet fixed, despite the ~46.83 Mya estimated split of Trichechidae and Dugongidae, thus lacking species-specific sequences as reflected in the neighbor joining tree (Figure 3D), which disagrees with the predictions of concerted evolution. This process, which has been described for many satDNAs, promotes fast sequence homogenization within a species or population, resulting in much higher interspecific than intraspecific differences (Plohl et al., 2012). Although interspecific satDNA sequence conservation is unexpected according to the concerted evolution model, interspecific homogeneity of centromeric satDNAs was observed in other mammalian groups, like in rodents from the *Peromyscus* genus (Smalec et al., 2019), in four squirrel monkeys (*Saimiri* genus; Valeri et al., 2020) and in two species of two-toed sloths from the genus *Choloepus* (Sena et al., 2020). In all these cases, a possible centromeric function was hypothesized. Moreover, the library model of satDNA evolution relies on the preexistence of a satDNA collection in related species, with the differences observed among the species mostly due to amplification-contraction events of these sequences pool, and does not imply in rapid sequence changes (Plohl et al., 2009). These could be the case of TMAsat evolution if considering the monomer variants as independent amplification-contraction units.

In addition to the centromeric localization in *T. manatus* and *T. inunguis*, we detected the CENPB-box like motif, another centromeric feature, twice in the TMAsat sequences of all four Sirenia species. In *T. manatus* and in *D. dugon*, both putative CENPB-boxes were located in conserved segments of TMAsat. Even though the CENPB-box-like motif found in TMAsat does not present all the nine nucleotides of the ECD, we cannot exclude its functional activity. Among *Peromyscus* species, the CENPB-box-like motifs found in the centromeric satDNA had between four and six conserved bases out of nine ECD nucleotides. It has been suggested that a divergent motif sequence may be required for functional activity in this group (Smalec et al., 2019), which could also be the case for manatees and the dugong. Divergent motif sequences have also been observed in the centromeric satDNAs of the African elephant (*L. africana*), nine-banded armadillo (*D. novemcintus*; Alkan et al., 2011) and in the two-toed sloths of the genus *Choloepus* (Sena et al., 2020).

The only genomes outside Sirenia in which the TMAsat sequence was found were those of the Order Proboscidea (elephants) and Hyracoidea (hyraxes), that together with Sirenia are reunited in Paenungulata, a subgroup of the Superorder Afrotheria (Foley et al., 2016). With only a few hits (with the short arrays mostly flanked by the transposable element L1), the TMAsat sequence is probably not a typical satDNA in these species. TMAsat in Sirenian probably evolved from these ancestral sequences still found in elephants and hyraxes, which could be the basis for both TMAsat1 and TMAsat2.

In the tree sirenians with sequenced genome, the most frequent organization of TMAsat arrays was the alternating TMAsat1 and TMAsat2 form. In the few exceptions, we found more consecutive TMAsat2 units than TMAsat1. Other satDNAs were found organized as a composite of two related units, mostly in the alternating form as TMAsat. This is the case of S1a-S1b in European brown frogs (Feliciello et al., 2006) and Tcast1a-Tcast1b in the red flour beetle *Tribolium castaneum* (Feliciello et al., 2011, 2014), in which the rolling circle amplification followed by substitutions by homologous recombination were proposed to explain the origin of the composite a-b arrays.

The sequenced genomes we used were generated from short reads (average 100–150bp) that do not cover the total length of the monomeric unit of TMAsat, resulting in an assembly that may not represent well the long satDNA arrays. Further analyses with Southern blot and dot blot experiments as well as long-reads sequencing may help clarify the overall organization of repeats in the genome and within the long satDNA arrays. As an example, Vondrak et al. (2020) using ultra-long nanopore reads found nine out of 11 putative satDNA sequences derived from short tandem arrays located within LTR-retrotransposons that occasionally expanded in length, and just two organized in long arrays typical of satDNA. In addition, the long-reads sequencing approach proved a valuable contribution in determining the origin of the satDNAs. Several satDNAs from plants and animals derived from tandem amplification of internal segments of TEs (Dias et al., 2015; Meštrović et al., 2015; Vondrak et al., 2020), as was the case of TMAsat described herein, that could be L1 related.

In conclusion, we reported for the first time the centromeric satDNA in the West Indian manatee, which seems to be present across Sirenia, a group with all extant species under threat of extinction. TMAsat monomers from *T. manatus*, *T. inunguis*, *D. dugon*, and *H. gigas* lack species-specific sequences, contradicting the predictions of concerted evolution. The TMAsat-like ancestral sequence is present in other Paenungulata, such as elephants and hyraxes, suggesting that TMAsat suffered an expansion within Sirenia less than ~69 Mya (Foley et al., 2016; de Souza et al., 2021), after the divergence of Sirenia from Proboscidea and Hyracoidea.

## Data Availability Statement

The datasets presented in this study can be found in online repositories. The names of the repository/repositories and accession number(s) can be found at: https://www.ncbi.nlm.nih.gov/genbank/, MW272776–MW272780.

## Ethics Statement

The animal study was reviewed and approved by SISBio/ICMBio permit 60829-2.

## Author Contributions

MV and GD conceived and designed the experiments, analyzed the data, and contributed to writing – original draft preparation. MV and AE performed the experiments. CM, YY-Y, and IS obtained the materials for molecular and cytological analyses. MV, GD, AE, GK, and MS contributed to writing – review and editing. GK and MS contributed to supervision and project administration. MS contributed to funding acquisition. All authors contributed to the article and approved the submitted version.

## Conflict of Interest

The authors declare that the research was conducted in the absence of any commercial or financial relationships that could be construed as a potential conflict of interest.

## Publisher’s Note

All claims expressed in this article are solely those of the authors and do not necessarily represent those of their affiliated organizations, or those of the publisher, the editors and the reviewers. Any product that may be evaluated in this article, or claim that may be made by its manufacturer, is not guaranteed or endorsed by the publisher.

## Footnotes

^1^ https://itol.embl.de/
