# Supplementary material for: First Description of a Satellite DNA in Manatees’ Centromeric Regions
Source: Front Genet. 2021 Aug 24;12:694866. doi: 10.3389/fgene.2021.694866 (PMC8421680; doi:10.3389/fgene.2021.694866)
Supplement: Supplementary file 1 [file Data_Sheet_1.PDF]

Supplementary Material

```
>TMAsat
ATTCACCACATGAAAGAGAAAGCAAAATACAGCGTTCTCCTTCATGCTGCTTAACTGAATCTACTGAAGTTTTCAGTTTT
GTTAGAAGAAAGTGTCCGCCCACCACAAGCTGCTCTCTCAAATGTTCTCTCAGTGGGAGAAGGCAGTAGAGTGAGGGCC
TAGCAGGAATTTGAATCCCCTGGTTTTCAAAGGGAACTTTGCATATGAGGAATGAAGGTATTGCTAGCTTACATAGCCA
AACAGGCAGATTGTTTGCCTTCAGCTAGAGAACAGCAGAAGAGGTTCAGTTTTGCTAACTAAAGCTTGTGTGCTTAACA
CGAGCATGTAGCTTCTTCCCTGCCTGAAAGAGAAAGCAAGAGCTTGCTGCTCTCAGTCTGGGGATGAAGGGAAAAAGAT
TCACTTTCTGGGTTTGAAAATCCCTCTGTGTTACATGCCTAAGAAAGTTGCCTGTGCATTGTCTACCTGGATAAAGTGTA
TGACTTTTCTCCCTTTCAAGTAGTTGAAGGCGTACAGGGTTTCAGCATTAAGGGAGGCATTCTGTGCTGCGCGGCTGAGA
ATACCGCTTTACAGACGGTATCGCTACAGGAGAAGAGAAACCGCGTTTCTGCCTTTCTGGAACACGAATGAGGCAAAAC
CGTAGTTGTGCTGTGTAAACACTTTGTCAGCAGCAAGTGTAAGTTCCGC
```

Supplementary Figure 1. TMAsat consensus sequence, in fasta format, generated by TAREAN.

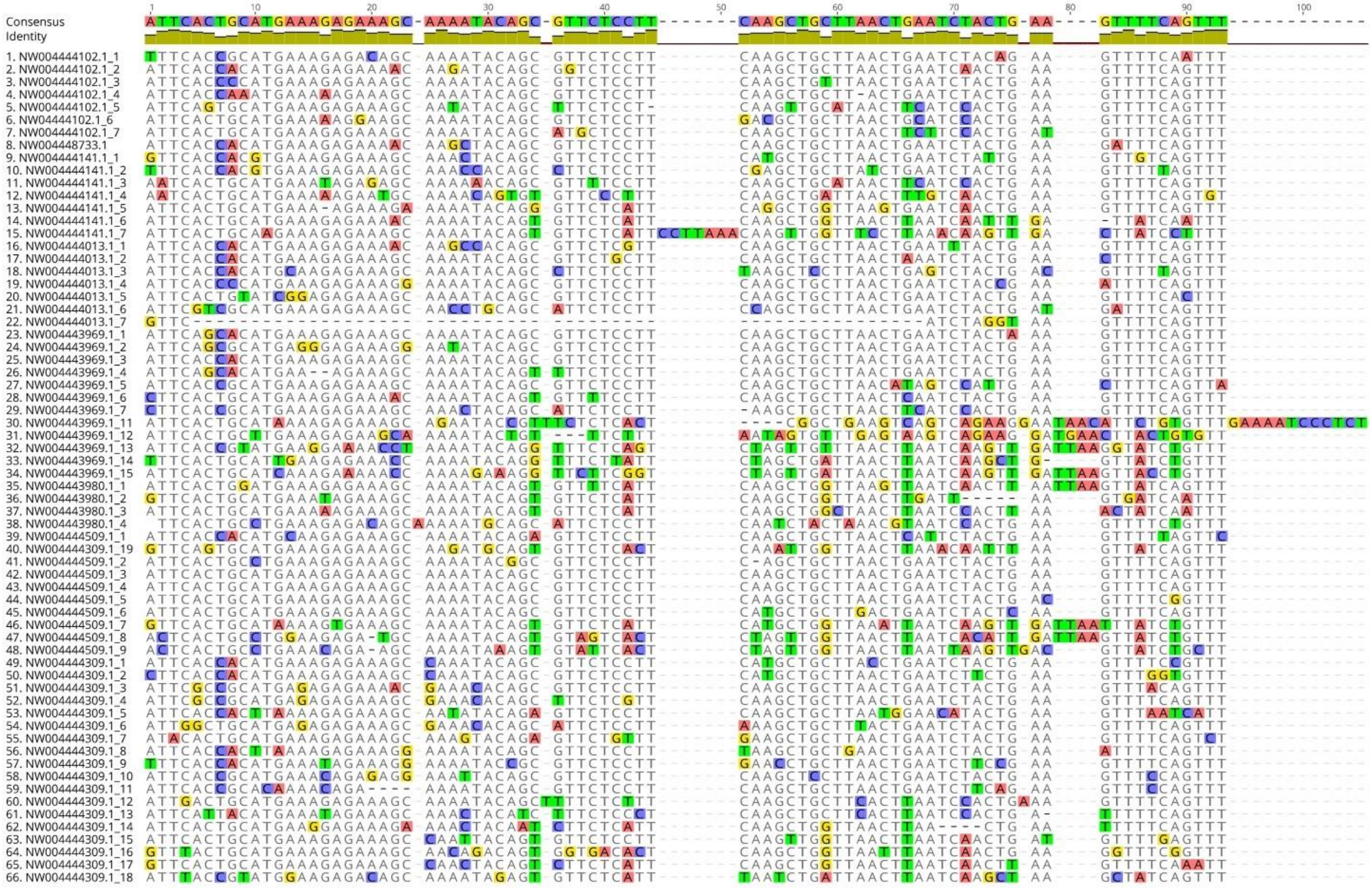

Supplementary Figure 2. Alignment of 66 monomers manually isolated from the *Trichechus manatus* assembled reference genome (accession GCA_000243295.1). Part one of eight.

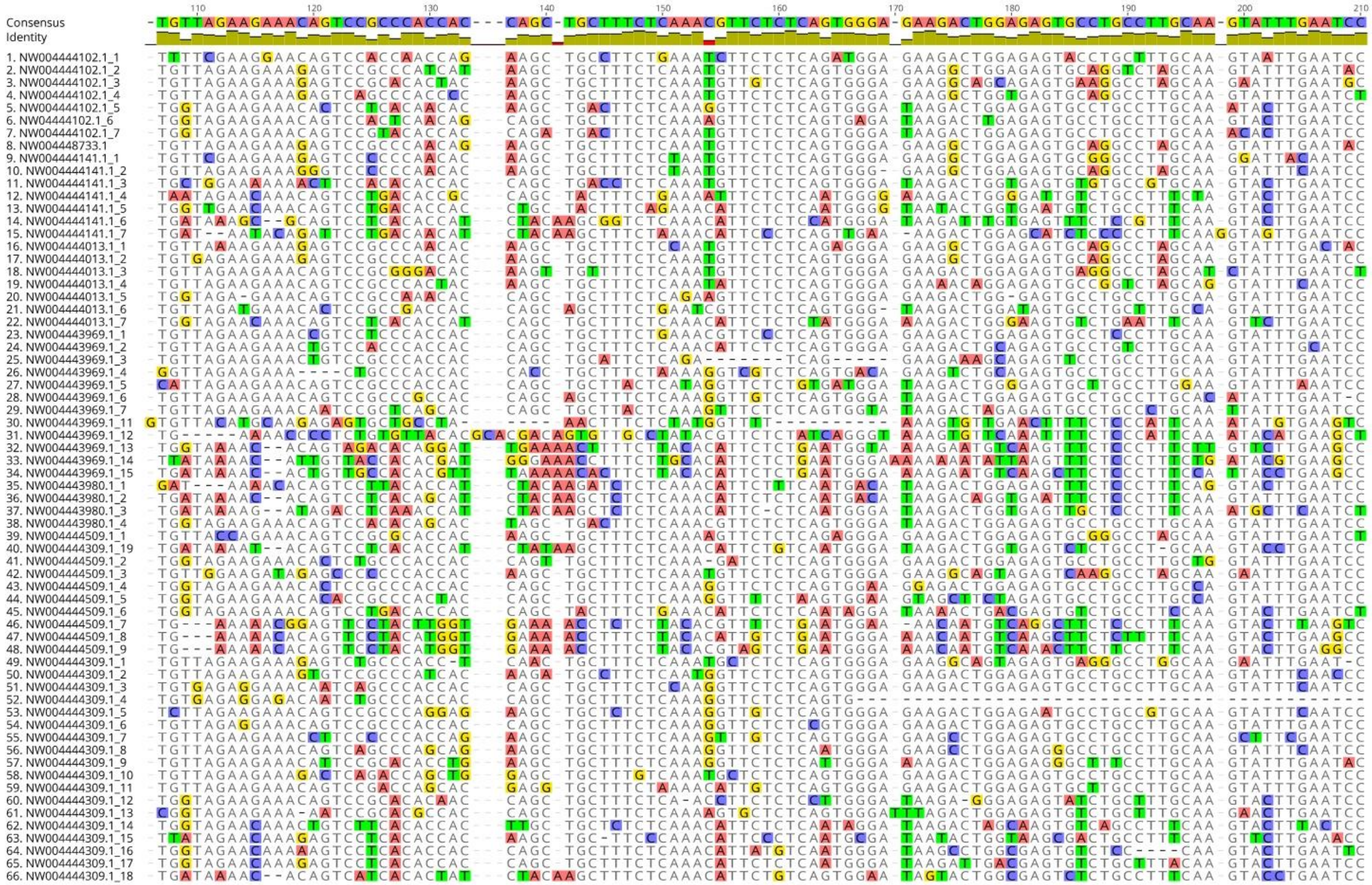

Supplementary Figure 2. Alignment of 66 monomers manually isolated from the *Trichechus manatus* assembled reference genome (accession GCA_000243295.1). Part two of eight.

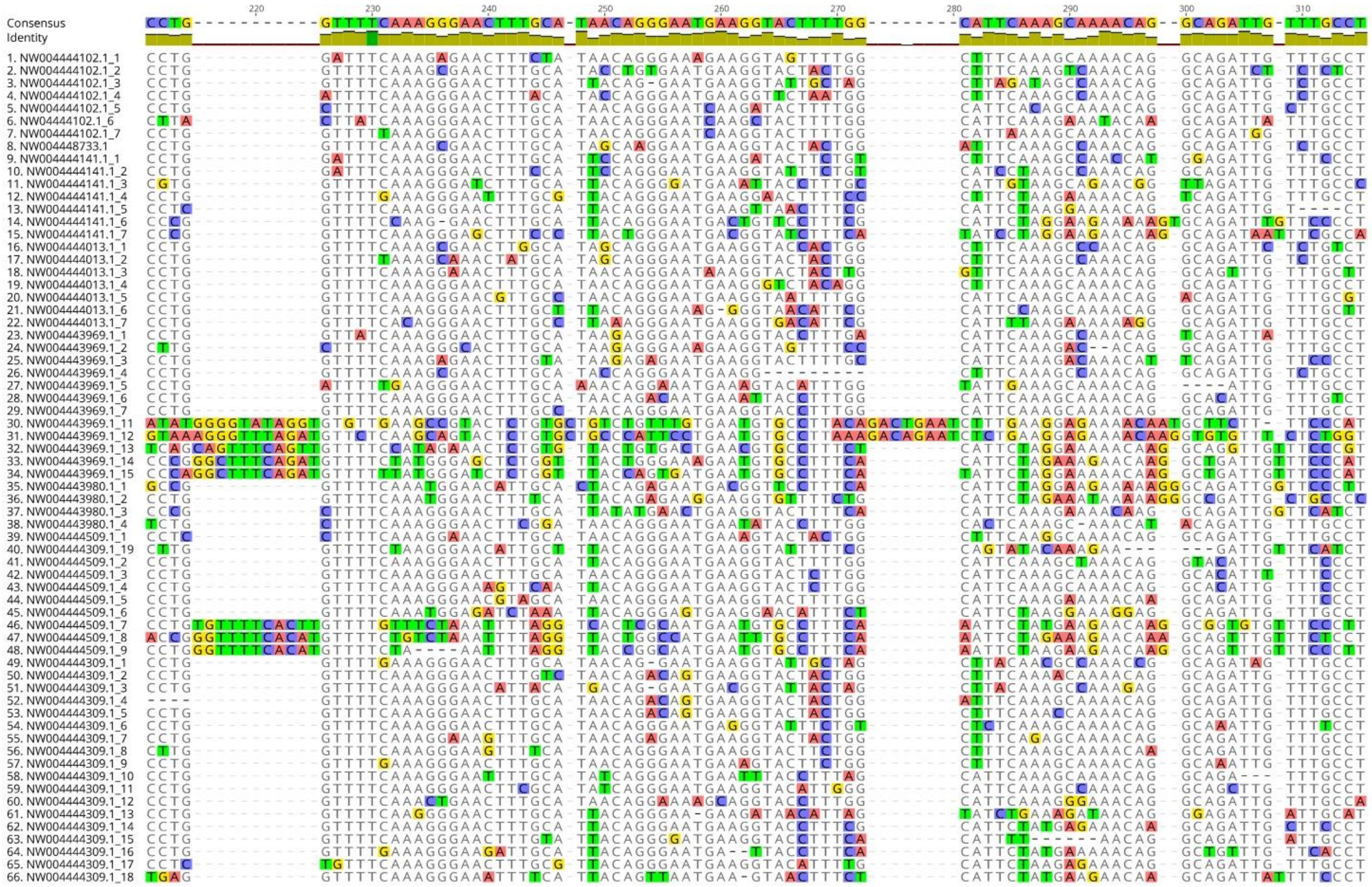

Supplementary Figure 2. Alignment of 66 monomers manually isolated from the *Trichechus manatus* assembled reference genome (accession GCA_000243295.1). Part three of eight.

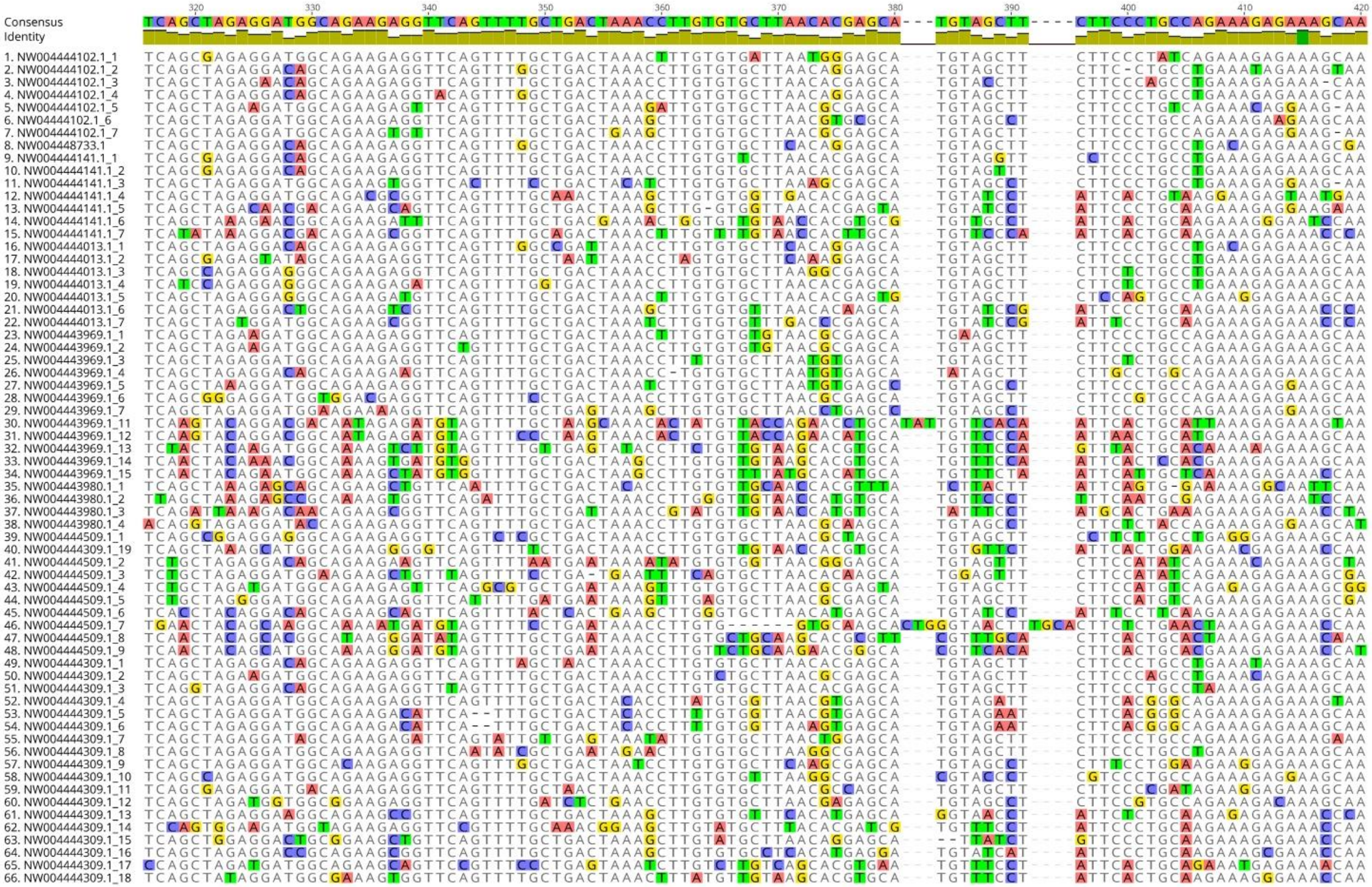

Supplementary Figure 2. Alignment of 66 monomers manually isolated from the *Trichechus manatus* assembled reference genome (accession GCA_000243295.1). Part four of eight.

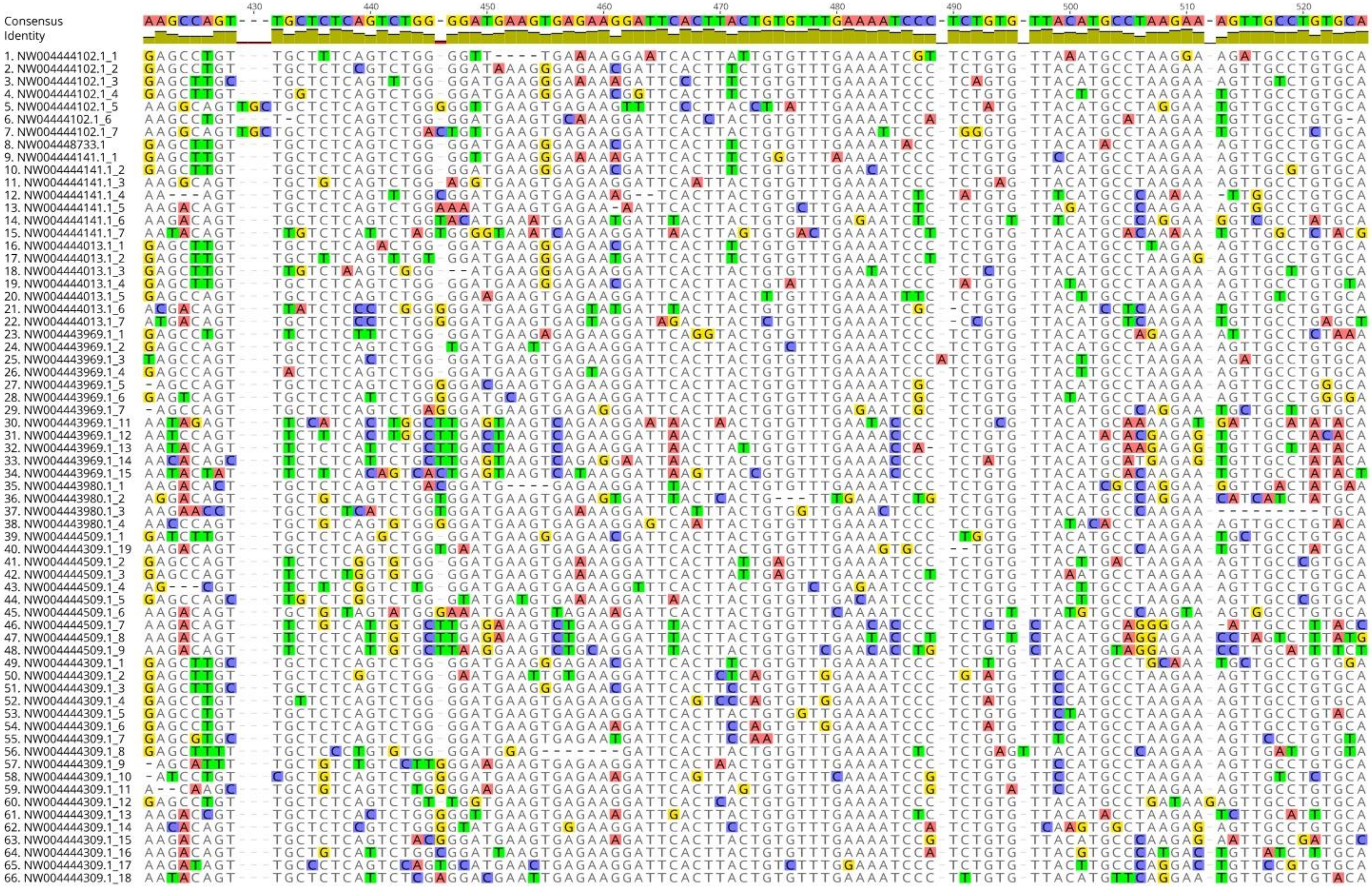

Supplementary Figure 2. Alignment of 66 monomers manually isolated from the *Trichechus manatus* assembled reference genome (accession GCA_000243295.1). Part five of eight.

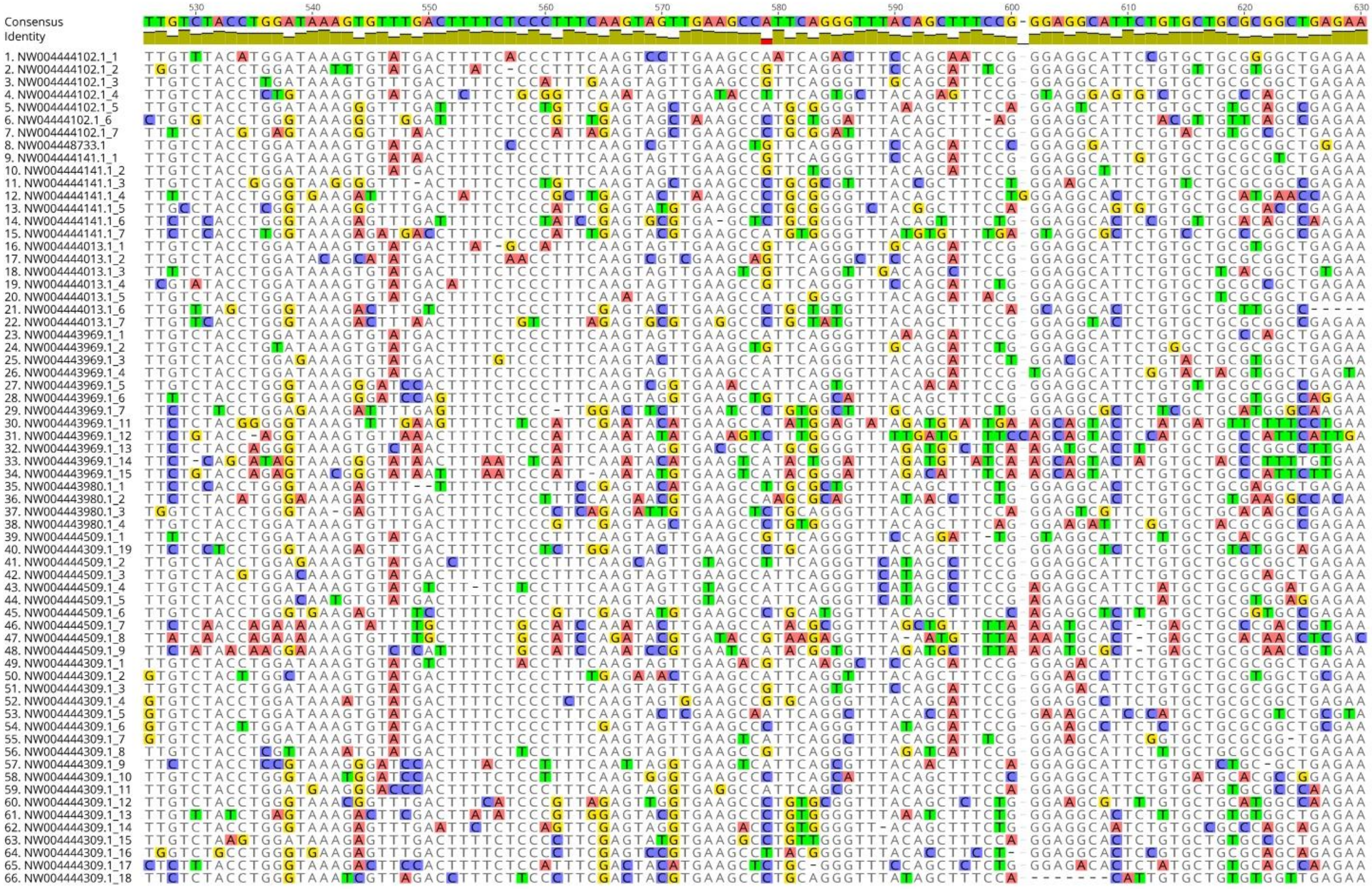

Supplementary Figure 2. Alignment of 66 monomers manually isolated from the *Trichechus manatus* assembled reference genome (accession GCA_000243295.1). Part six of eight.

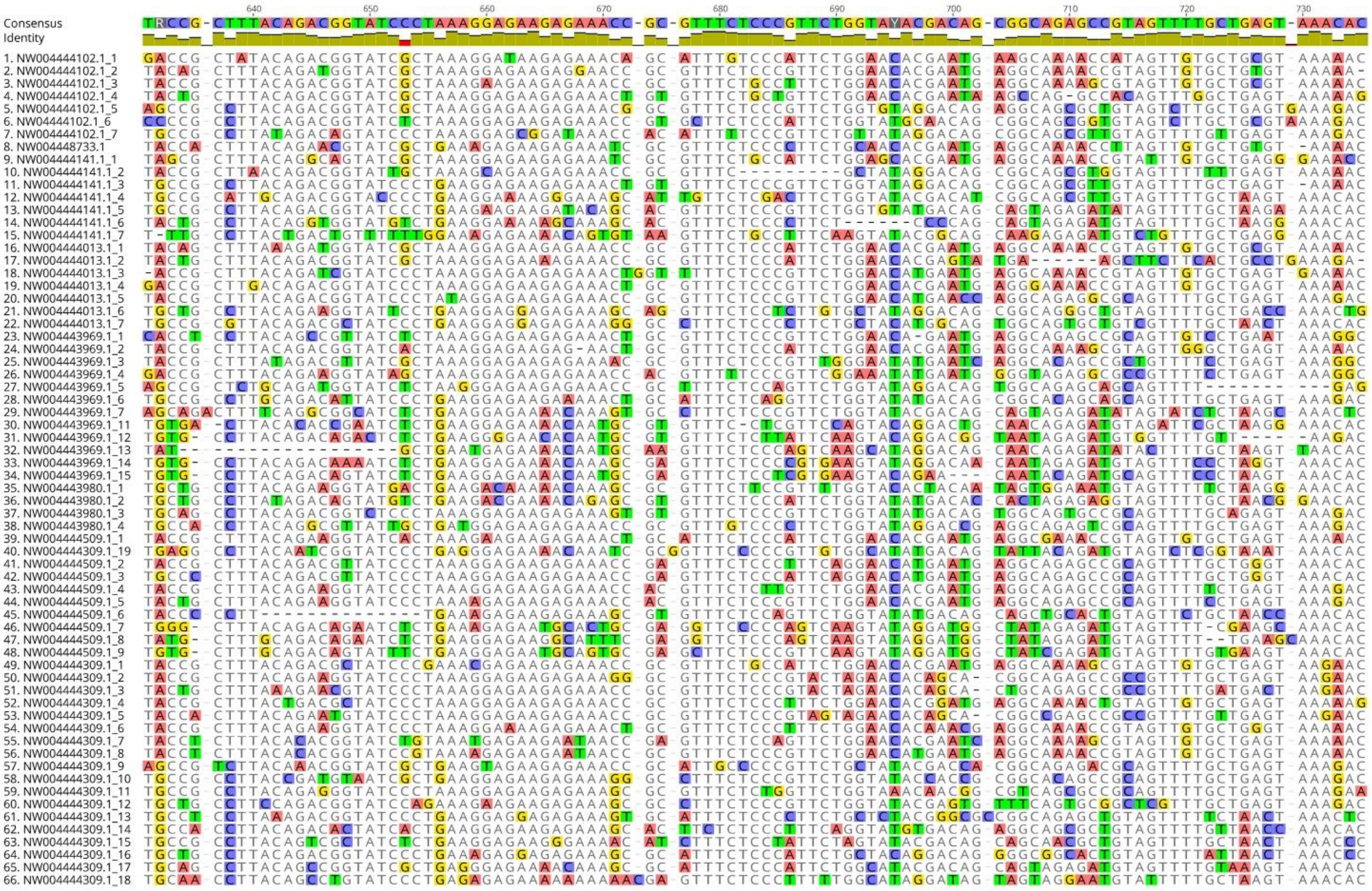

Supplementary Figure 2. Alignment of 66 monomers manually isolated from the *Trichechus manatus* assembled reference genome (accession GCA_000243295.1). Part seven of eight.

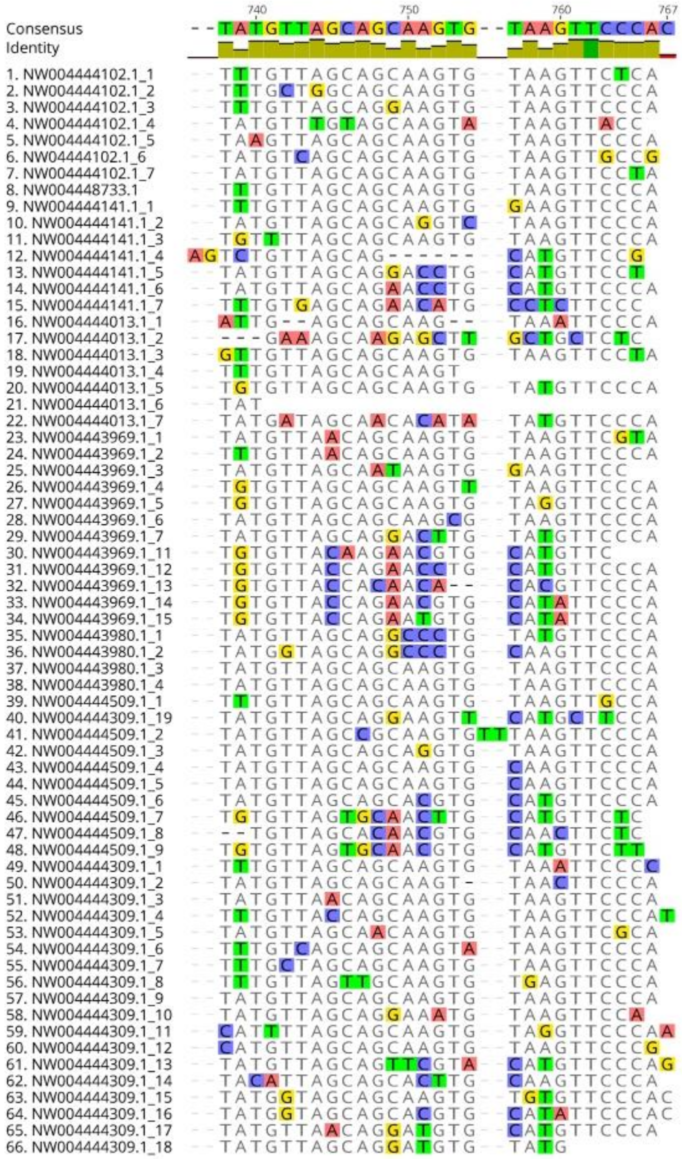

Supplementary Figure 2. Alignment of 66 monomers manually isolated from the *Trichechus manatus* assembled reference genome (accession GCA_000243295.1). Part eight of eight.

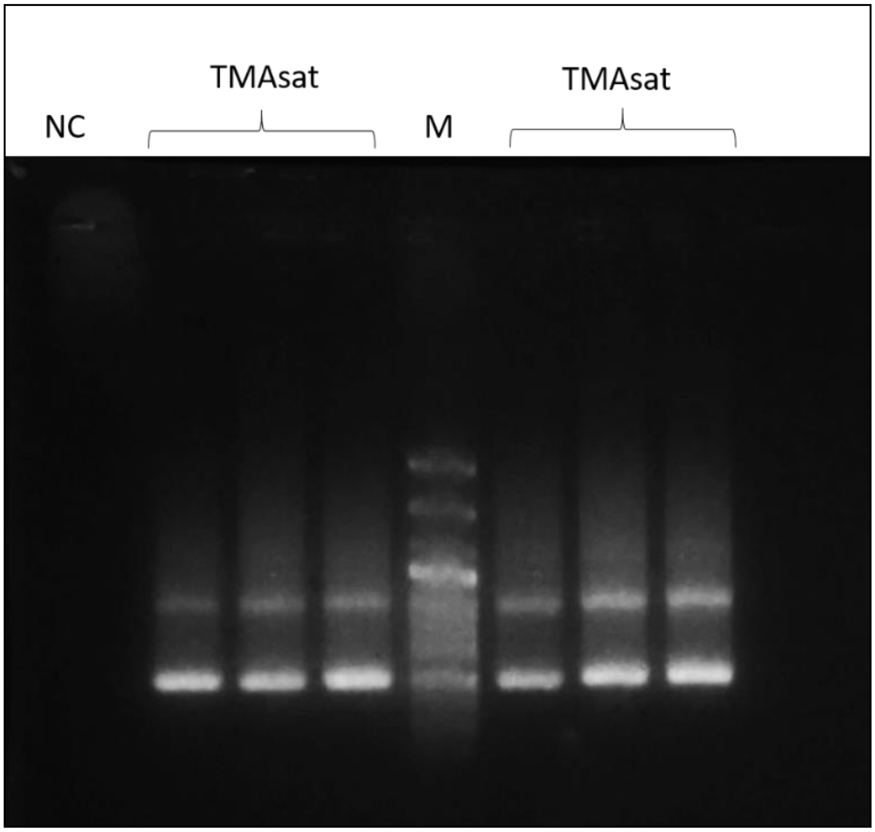

Supplementary Figure 3. Agarose gel (1%) showing the PCR products of *Trichechus inunguis* using the TMAsat primers. M - DNA size marker of 100 bp ladder; NC – Negative control (no DNA).

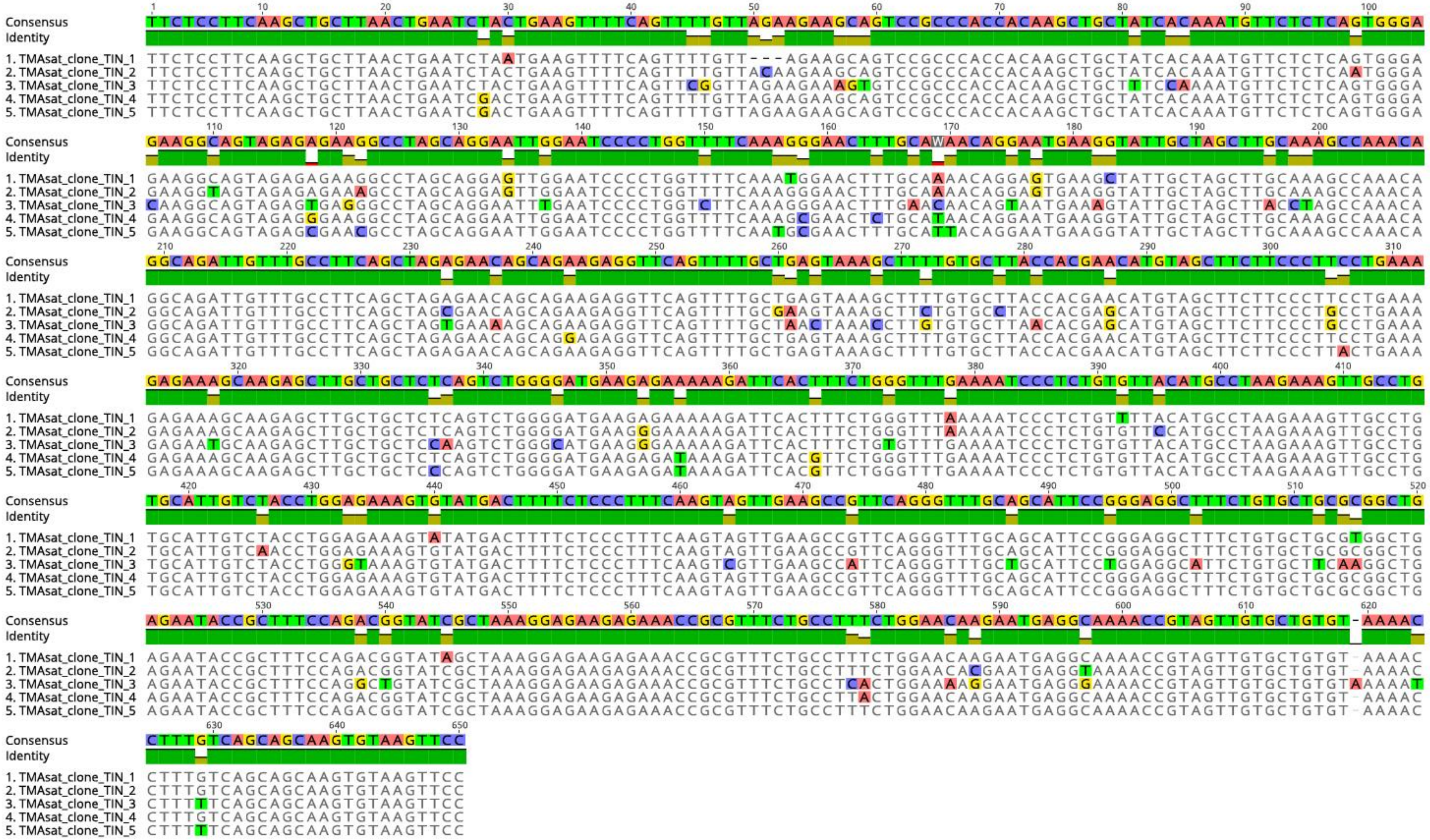

Supplementary Figure 4. Alignment of five TMAsat monomers cloned and sequenced from the *Trichechus inunguis* genome (accession MW272776- MW272780).

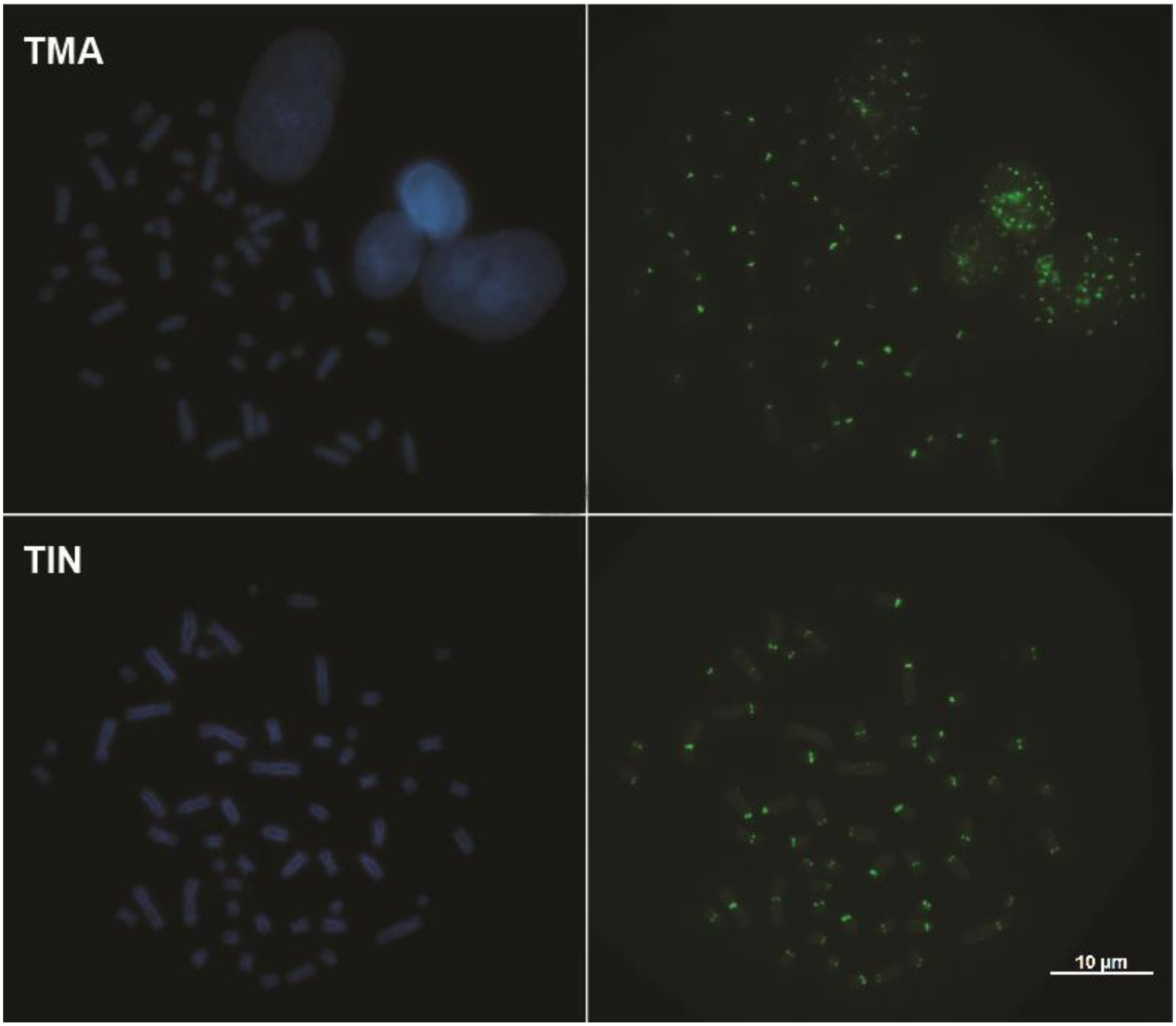

Supplementary Figure 5. Metaphases of *T. manatus* (TMA) and *T. inunguis* (TIN) after FISH with the TMAsat probe. Channels are presented separately, DAPI on left in blue and TMAsat probe on right in green.

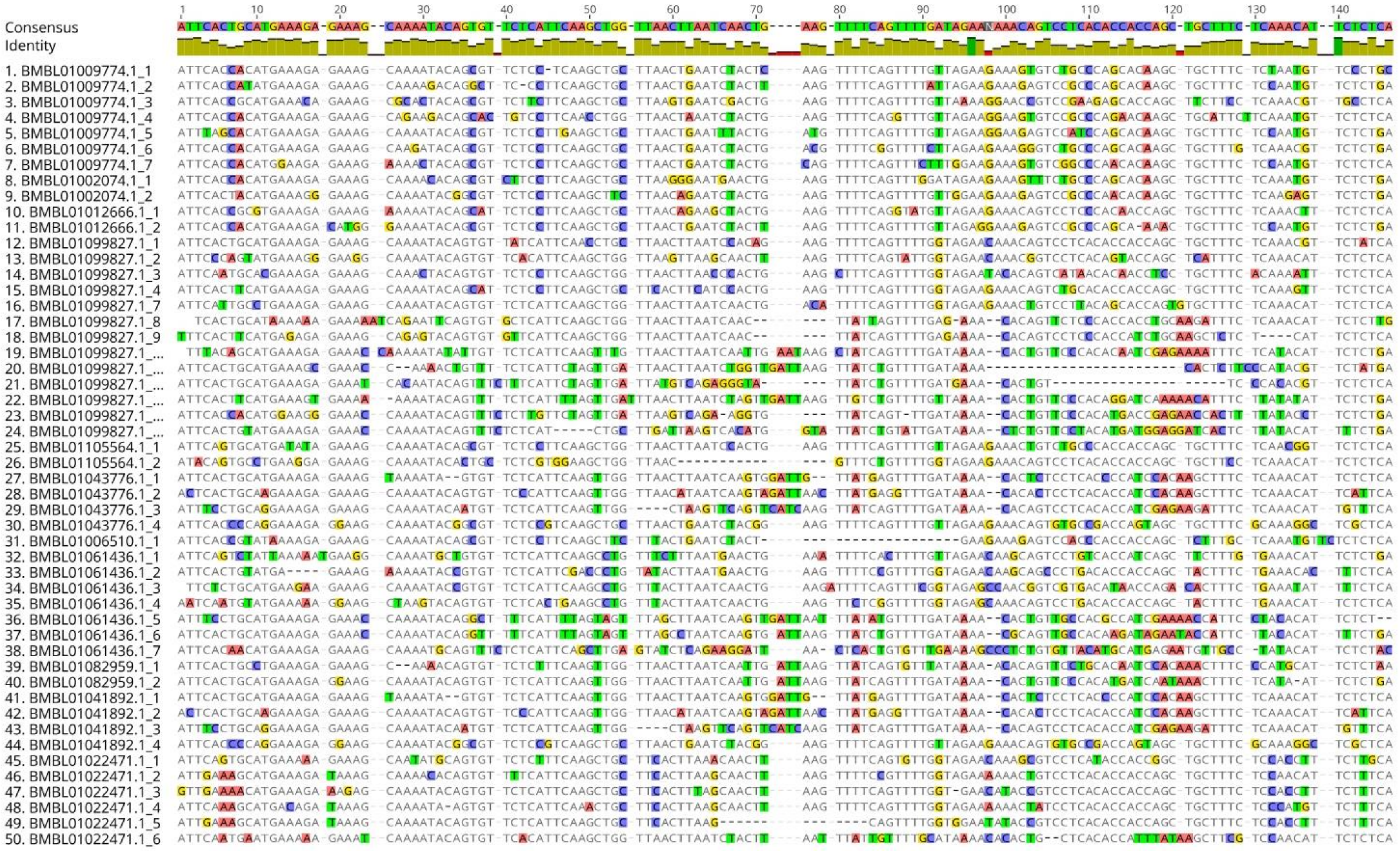

Supplementary Figure 6. Alignment of 50 monomers manually isolated from the *Dugong dugon* assembled reference genome (accession GCA_015147995.1). Part one of six.

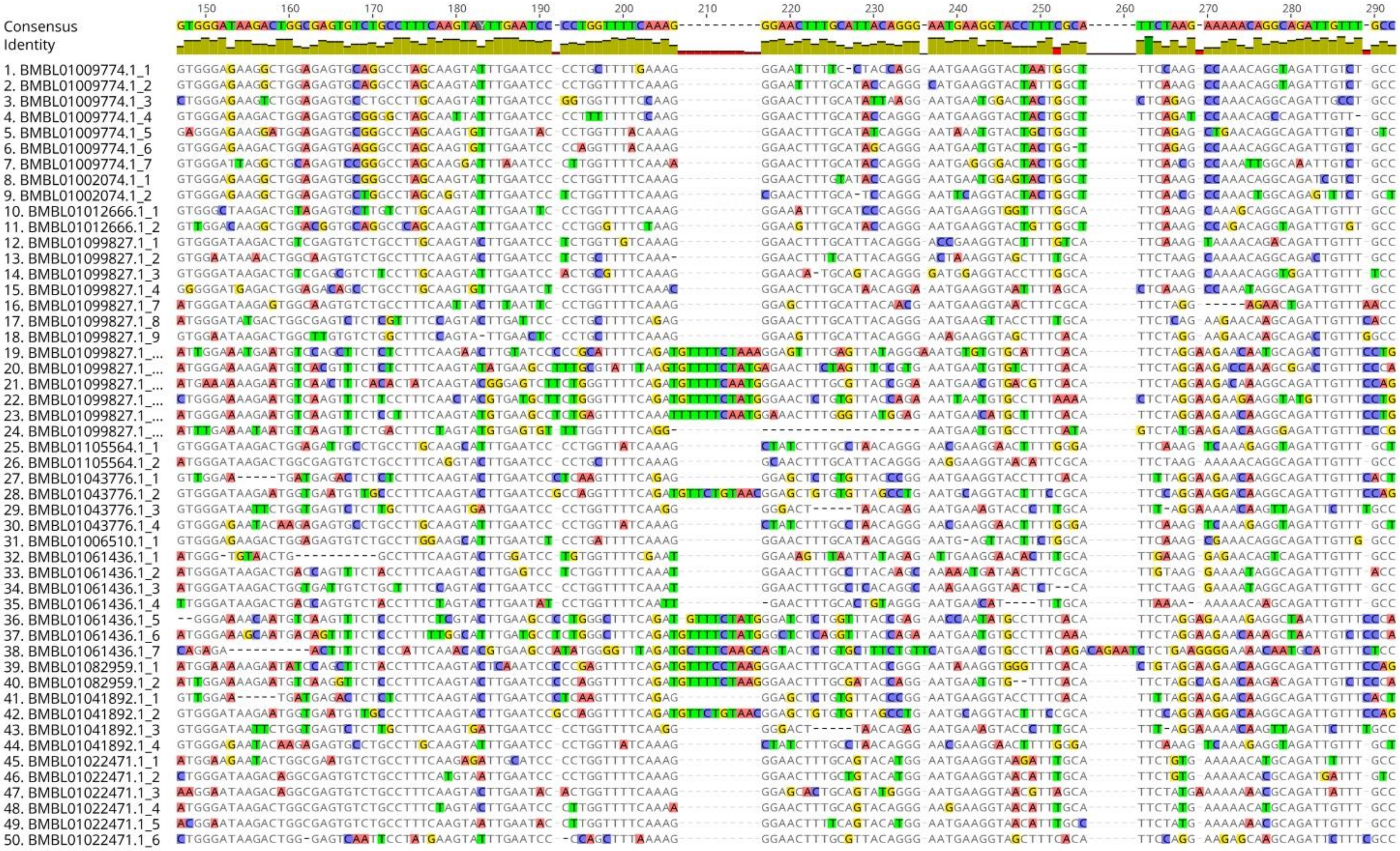

Supplementary Figure 6. Alignment of 50 monomers manually isolated from the *Dugong dugon* assembled reference genome (accession GCA_015147995.1). Part two of six.

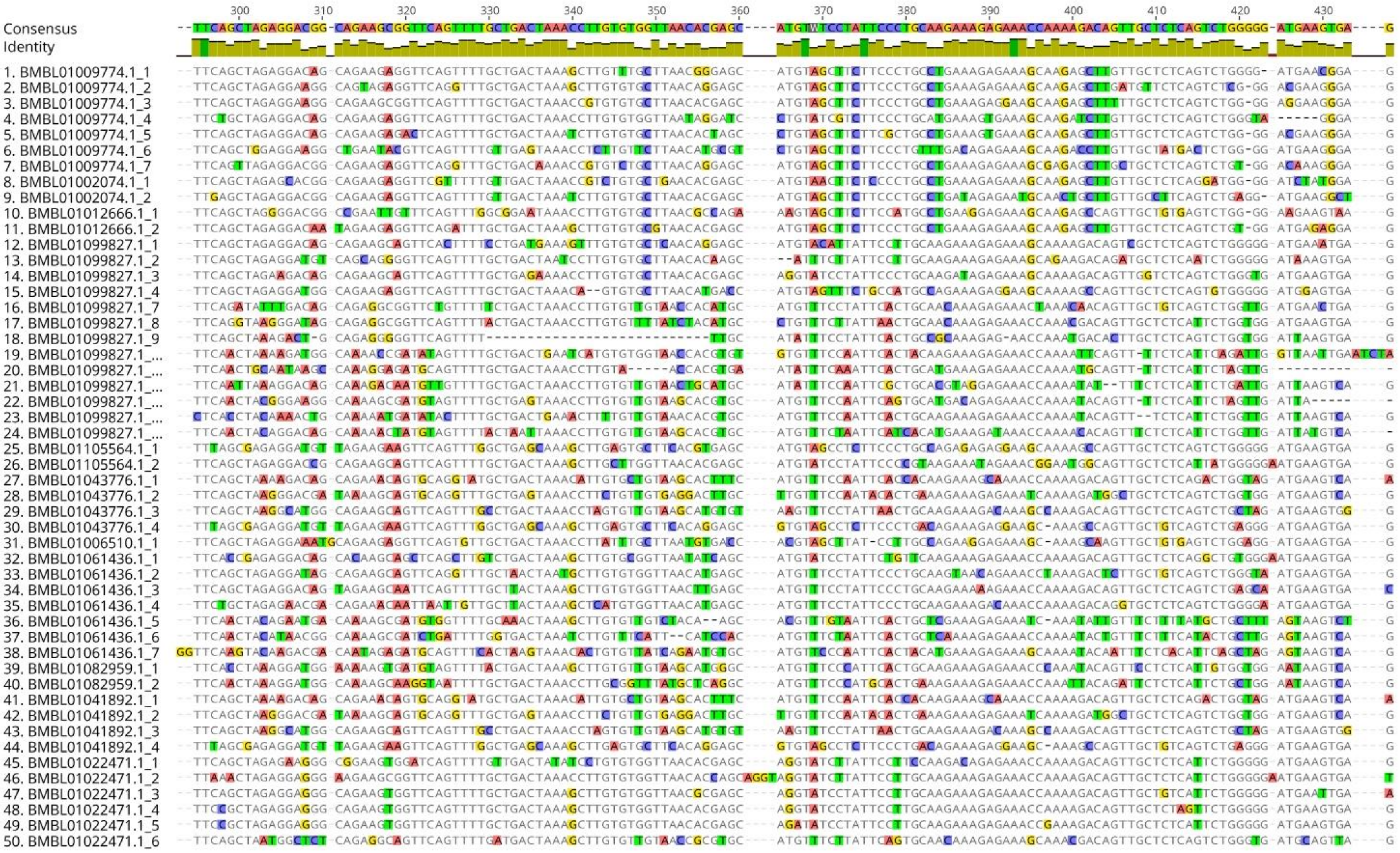

Supplementary Figure 6. Alignment of 50 monomers manually isolated from the *Dugong dugon* assembled reference genome (accession GCA_015147995.1). Part three of six.

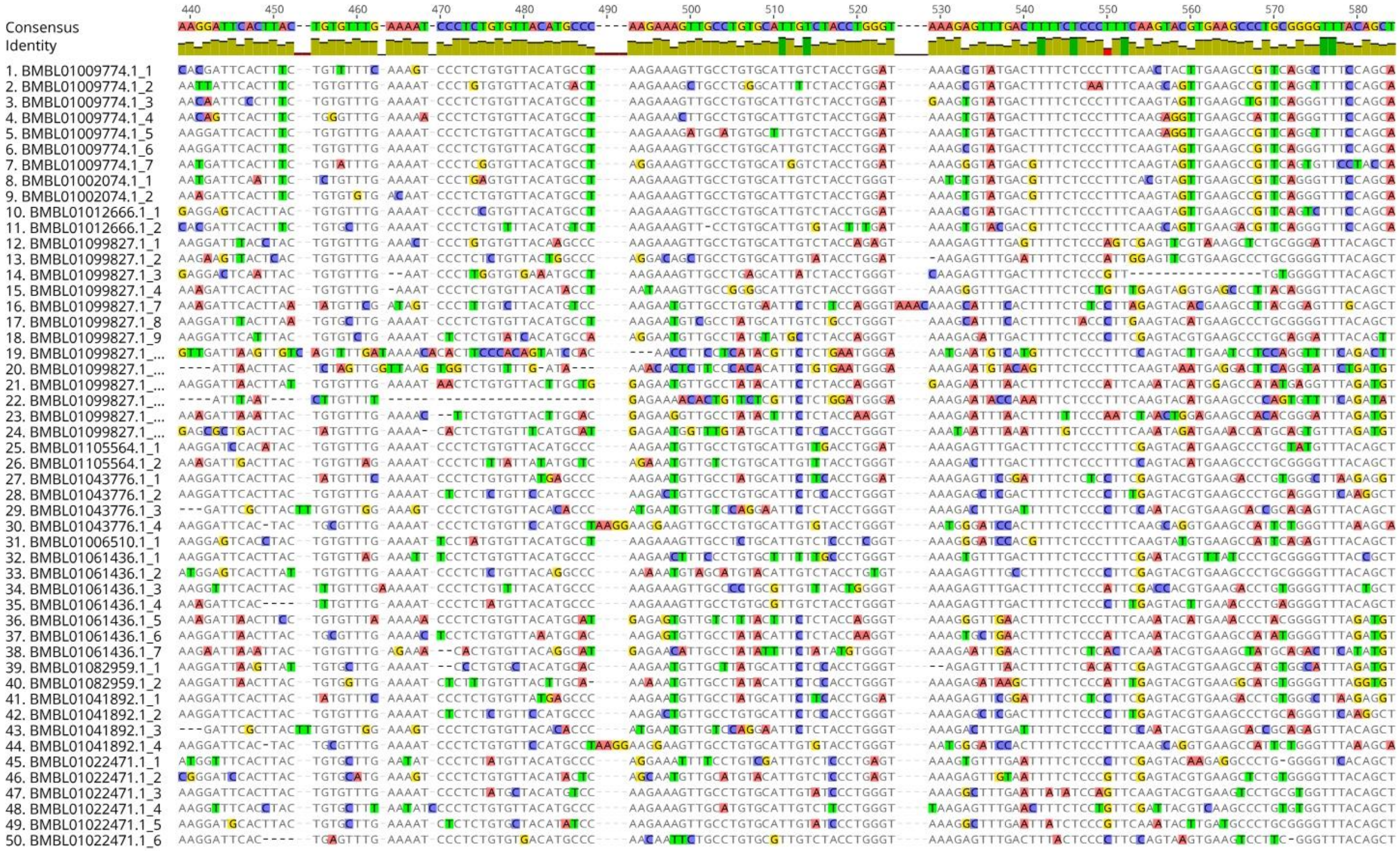

Supplementary Figure 6. Alignment of 50 monomers manually isolated from the *Dugong dugon* assembled reference genome (accession GCA_015147995.1). Part four of six.

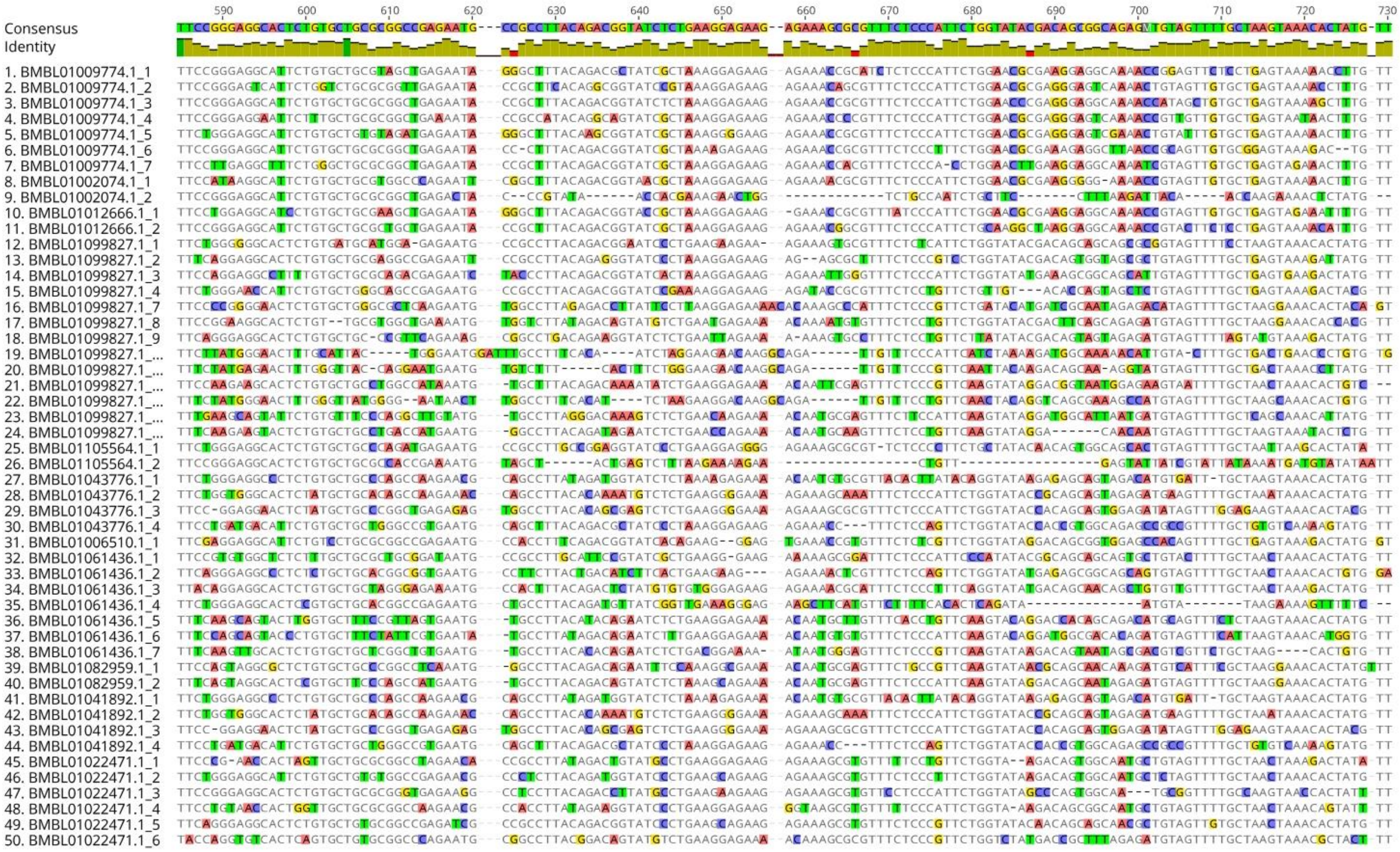

Supplementary Figure 6. Alignment of 50 monomers manually isolated from the *Dugong dugon* assembled reference genome (accession GCA_015147995.1). Part five of six.

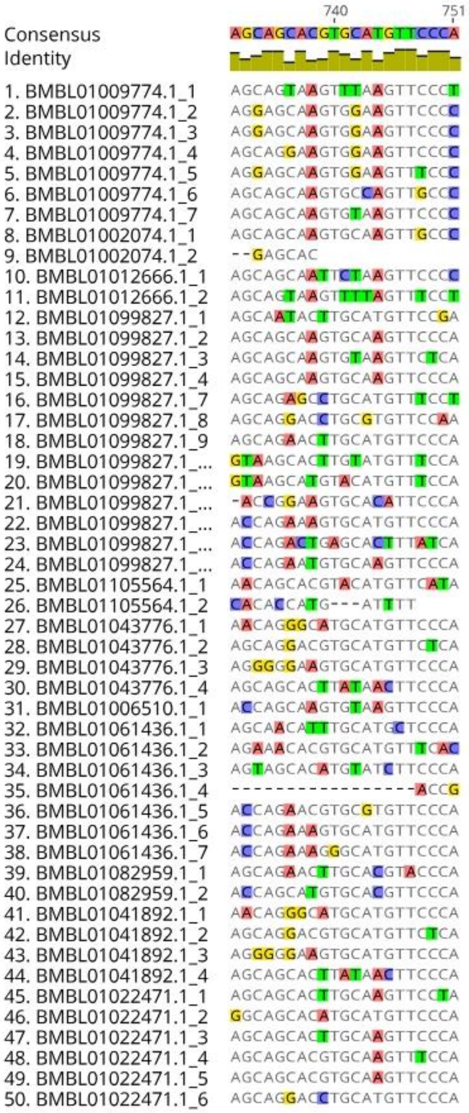

Supplementary Figure 6. Alignment of 50 monomers manually isolated from the *Dugong dugon* assembled reference genome (accession GCA_015147995.1). Part six of six.

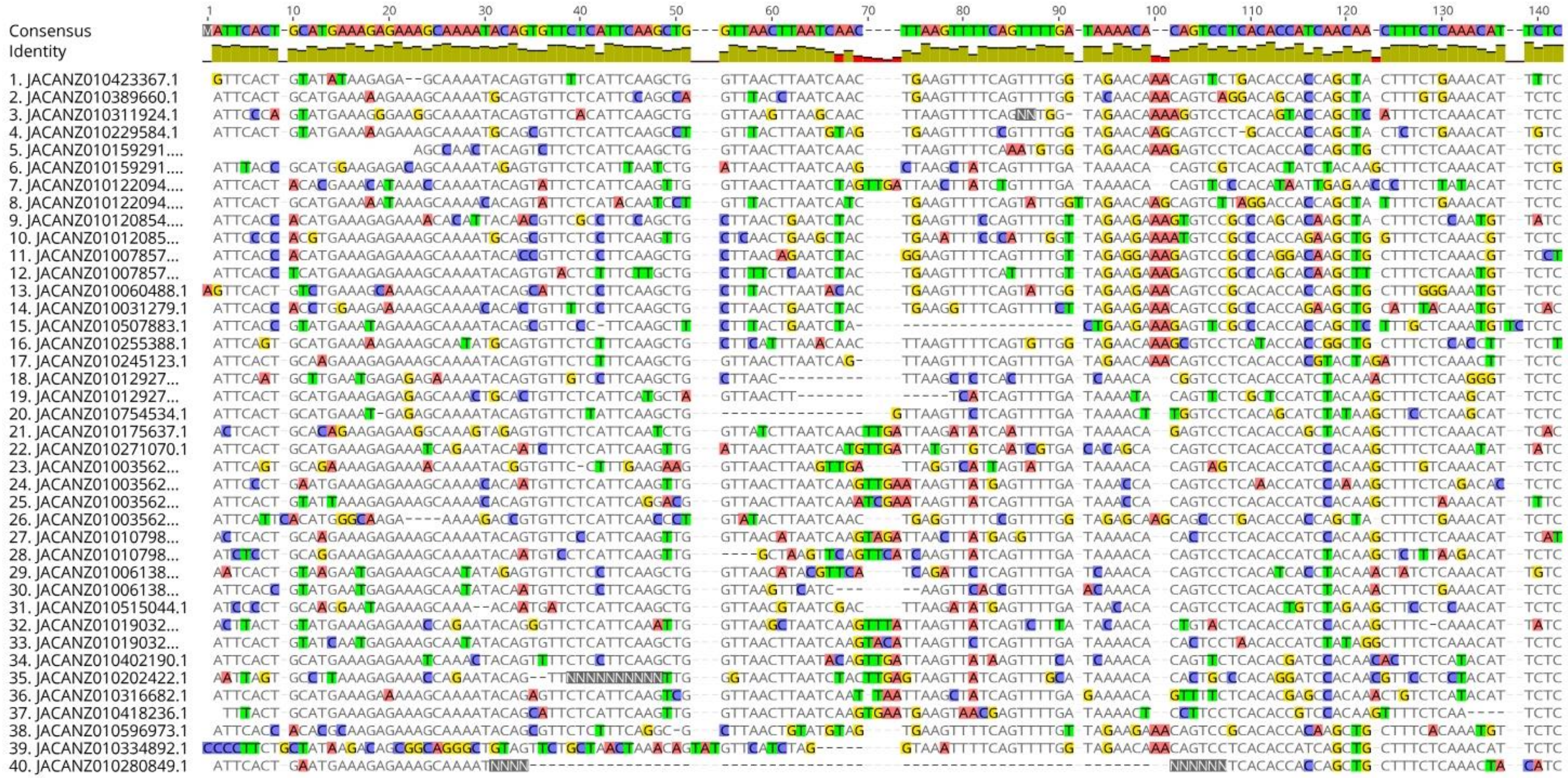

Supplementary Figure 7. Alignment of 40 monomers manually isolated from the *Hydrodamalis gigas* assembled reference genome (accession GCA_013391785.1). Part one of six.

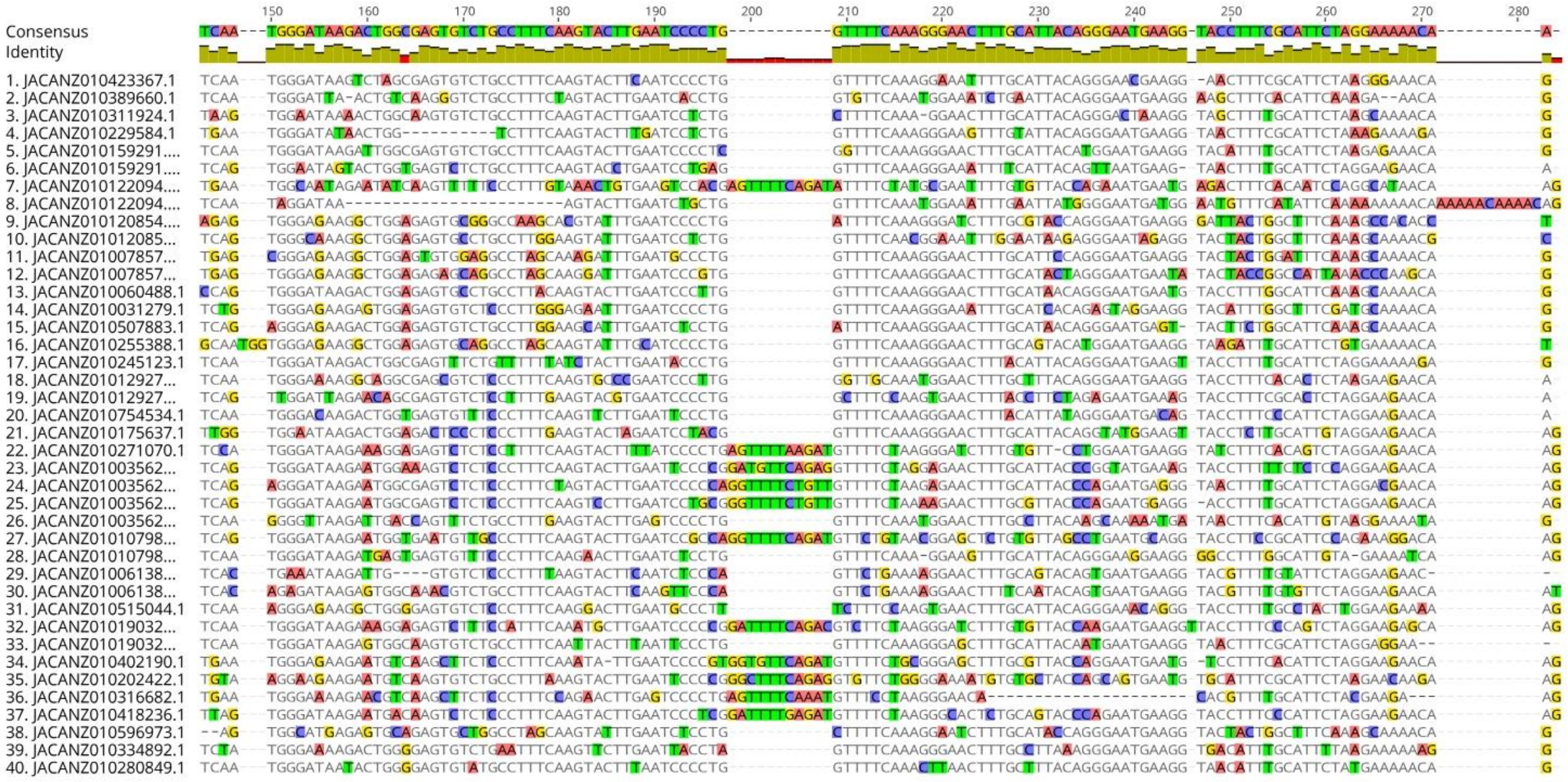

Supplementary Figure 7. Alignment of 40 monomers manually isolated from the *Hydrodamalis gigas* assembled reference genome (accession GCA_013391785.1). Part two of six.

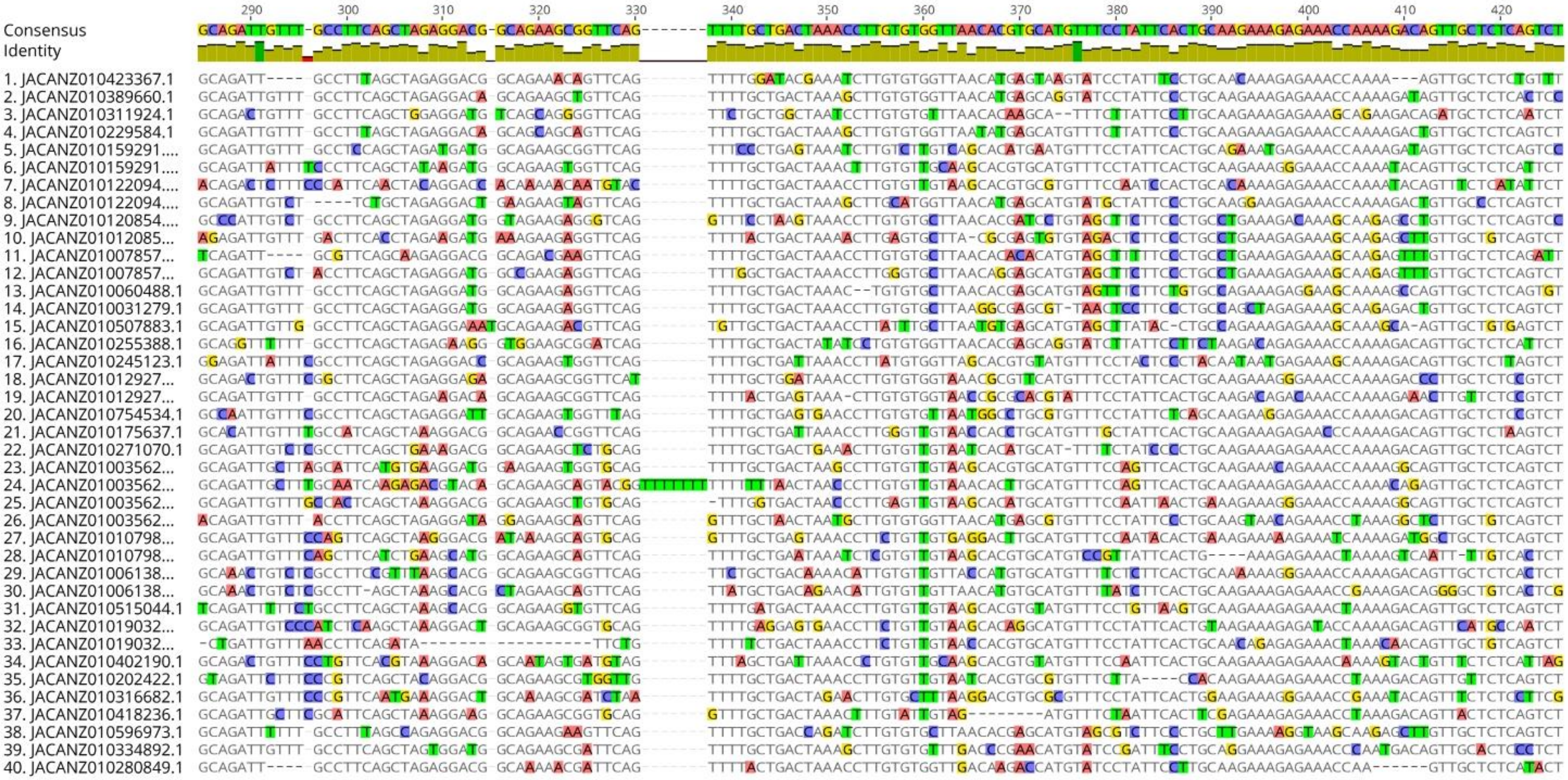

Supplementary Figure 7. Alignment of 40 monomers manually isolated from the *Hydrodamalis gigas* assembled reference genome (accession GCA_013391785.1). Part three of six.

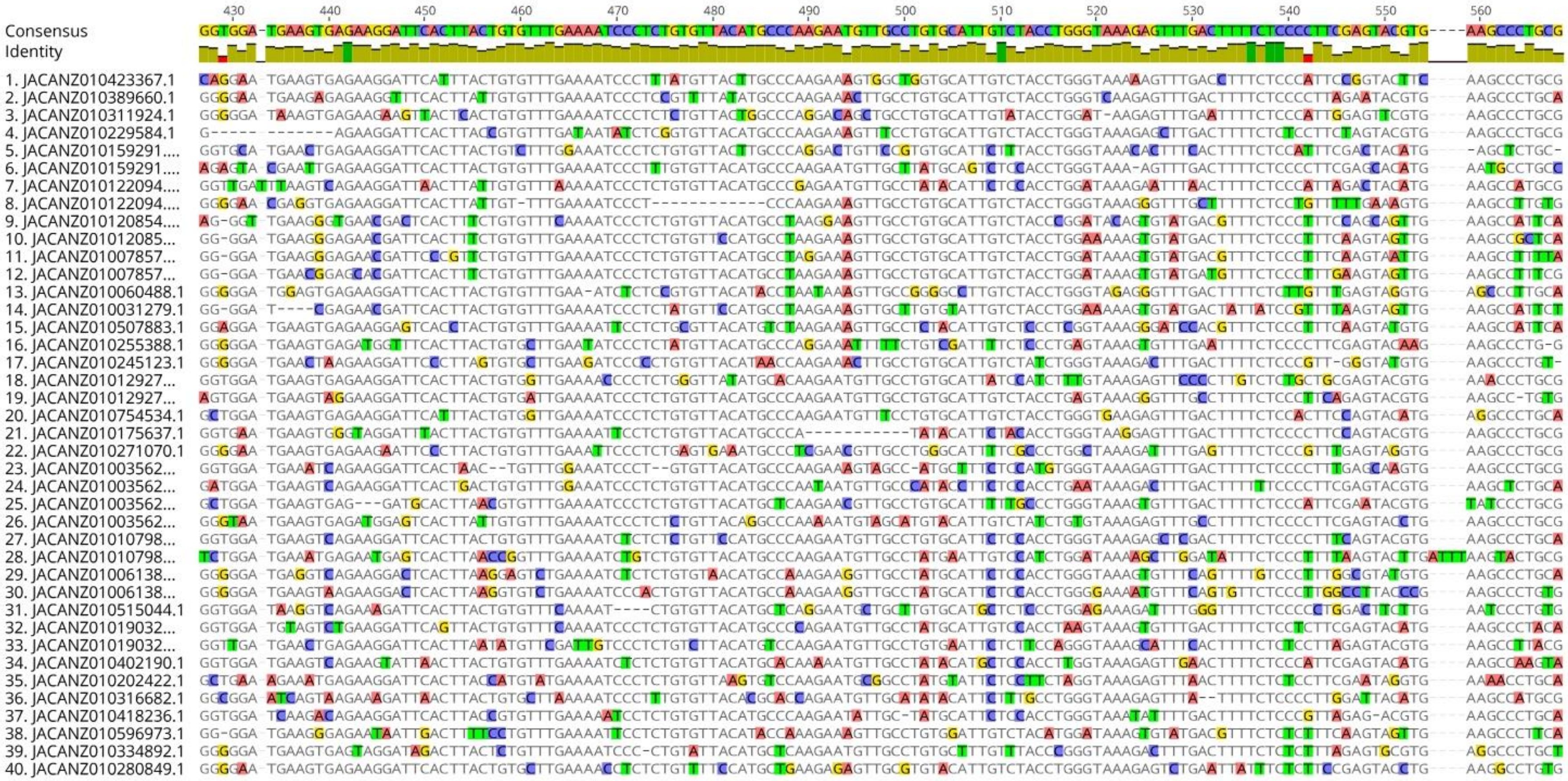

Supplementary Figure 7. Alignment of 40 monomers manually isolated from the *Hydrodamalis gigas* assembled reference genome (accession GCA_013391785.1). Part four of six.

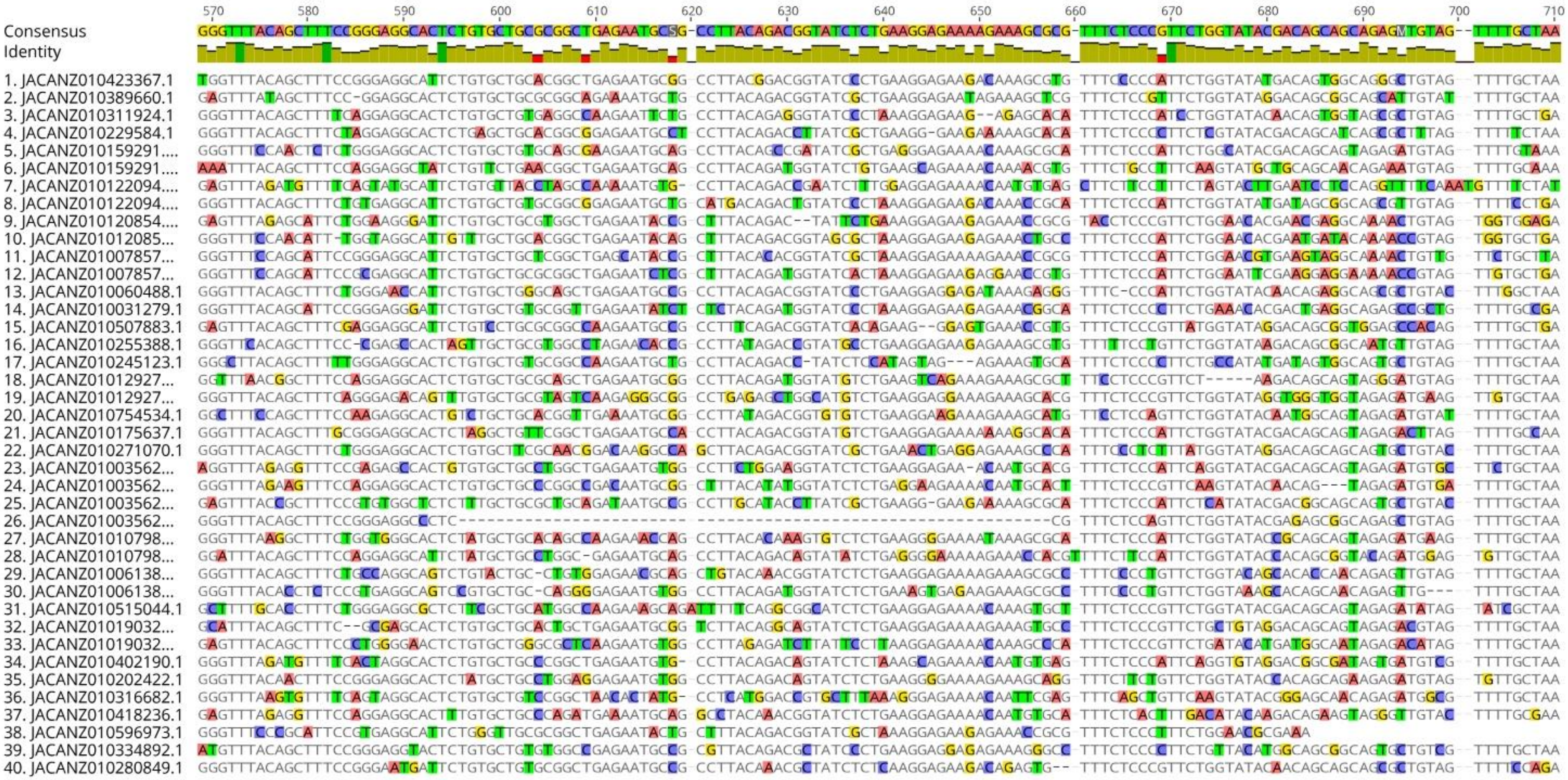

Supplementary Figure 7. Alignment of 40 monomers manually isolated from the *Hydrodamalis gigas* assembled reference genome (accession GCA_013391785.1). Part five of six.

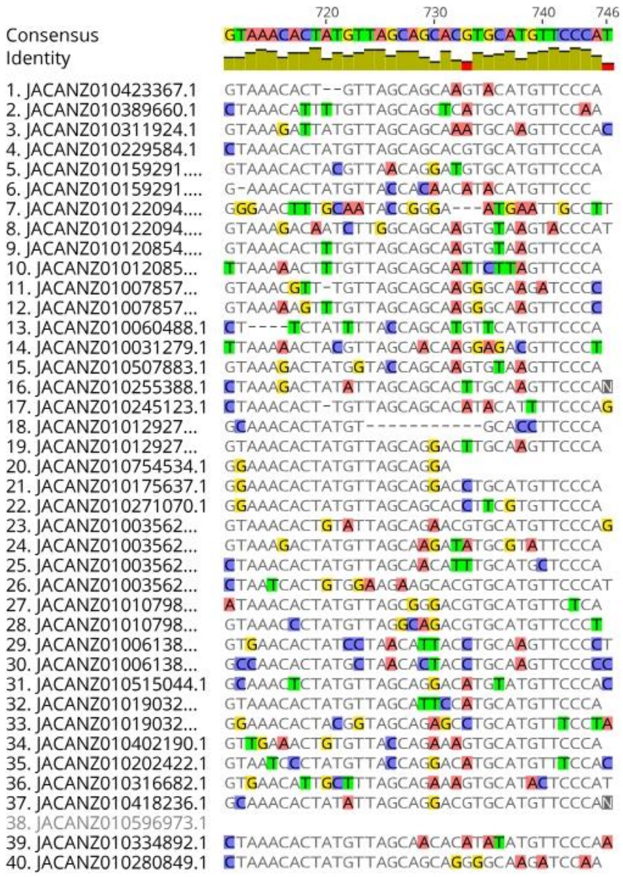

Supplementary Figure 7. Alignment of 40 monomers manually isolated from the *Hydrodamalis gigas* assembled reference genome (accession GCA_013391785.1). Part six of six.

Supplementary Table 1. Estimates of average nucleotide divergence (number of base substitutions per site) over all sequence pairs within and between species.

	TMA	TIN	DDU	HGI
TMA	0.32			
TIN	0.285	0.06		
DDU	0.354	0.357	0.37	
HGI	0.342	0.356	0.355	0.33

TMA: *T. manatus*, TIN: *T. inunguis*, DDU: *D. dugon*, HGI: *H. gigas*

Supplementary Table 2. Blast searches using the TMAsat Most Common Sequence (MCS) from *Trichechus manatus* as query against the GenBank Mammalia (NCBI:txid40674) wgs database excluding Sirenia (NCBI:txid9774).

Species	Description	Coverage (%)	Identity [%]	E-value	Accession number
Loxodonta africana	isolate ISIS603380 cont3.14052, whole genome shotgun sequence	60	68.94	7,00E-10	AAGU03014053.1
Loxodonta africana	isolate ISIS603380 cont3.64816, whole genome shotgun sequence	62	68.29	8,00E-09	AAGU03064817.1
Elephas maximus	isolate Icky scaffold_1324, whole genome shotgun sequence	60	68.51	3,00E-08	JABTCH010005241.1
Elephas maximus	isolate Icky scaffold_65, whole genome shotgun sequence	35	70.22	1,00E-07	JABTCH010002495.1
Elephas maximus	isolate Icky scaffold_751, whole genome shotgun sequence	58	65.02	1,00E-07	JABTCH010005233.1
Loxodonta africana	isolate ISIS603380 cont3.85517, whole genome shotgun sequence	58	65.02	1,00E-07	AAGU03085518.1
Elephas maximus	isolate Icky scaffold_1495, whole genome shotgun sequence	47	68.00	1,00E-06	JABTCH010003728.1
Loxodonta africana	isolate ISIS603380 cont3.56850, whole genome shotgun sequence	47	68.00	1,00E-06	AAGU03056851.1
Procavia capensis	isolate Dallas Zoo Isis #01D731 contig_177334, whole genome shotgun sequence	13	75.53	4,00E-06	ABRQ02177336.1
Heterohyrax brucei	isolate US064 HetBruBak_scaffold_45440, whole genome shotgun sequence	6	89.36	1,00E-05	PVJQ01022728.1
Loxodonta africana	isolate ISIS603380 cont3.91379, whole genome shotgun sequence	26	70.17	5,00E-05	AAGU03091380.1
Elephas maximus	isolate Icky scaffold_624, whole genome shotgun sequence	13	74.19	5,00E-05	JABTCH010004173.1
Loxodonta africana	isolate ISIS603380 cont3.14053, whole genome shotgun sequence	7	84.91	5,00E-05	AAGU03014054.1

Supplementary Sequence File 1. TMAsat monomeric sequences (in fasta) manually isolated from the *Trichechus manatus* assembled reference genome (accession GCA_000243295.1).

>NW004444102.1_1
TTTCACCGCATGAAAGAGACAGCAAAATACAGCGTTCTCCTTCAAGCTGCTTAACTGAATCTACAGAAGTT
TTCAATTTTTTTCGAAGGAACAGTCCACCAACCAGAAGCTGCCTTCTGAAATCTTCTCTCAGATGGAGAAGA
CTGGAGAGTACCTGCTTTGCAAGTAATTGAATCCCCTGGATTTCAAAGAGAACTTTCTATAACAGGGAAAG
AAGGTAGTTTTGGCTTTCAAAGCAAAACAGGCAGATTGTTTGCCTTCAGCGAGAGGATGGCAGAAGAGGTT
CAGTTTTGCTGACTAAACTTTGTGTGATTAATGGGAGCATGTAGCTTCTTCCCTATCAGAAAGAGAAAGCAA
GAGCCTGTTGCTTTCAGTCTGGGGTTTGAAAAGGAATCACTTATTGTGTTTGAAAATCGTTCTGTGTTAAAT
GCCTAAGGAAGATGCCTGTGCATTGTTTACATGGATAAAGTGTATGACTTTTCACCCTTTCAAGTCCTTGAA
GCCAATCAGACTTTCCAGCAATCCGGGAGGCATTCCGTGCTGCGGGGCTGAGAAGACCGCTATACAGACGG
TATCGCTAAAGGATAAGAGAAACAGCATTTGTCCCATTCTGGAACACGAATGAAGCAAAACCATAGTTGTG
CTGCGTAAAAACTTTGTTAGCAGCAAGTGTAAGTTCTCA
>NW004444102.1_2
ATTCACCACATGAAAGAGAAAACAAGATACAGCGGTCTCCTTCAAGCTGCTTAACTGAATCAACTGAAGTT
TTCAGTTTTGTTAGAAGAAAGAGTCCGCCCATCATAAGCTGCTTTCTCAAATGTTCTCTCAGTGGGAGAAGG
CTGGAGAGTGCAGGTCTAGCAAGTATTTGAATACCCTGGTTTTCAAAGCGAACTTTGCATACCTGTGAATG
AAGGTACTACTGGCTTTCAAAGTCAAACAGGCAGATCTTCTCTCTTCAGCTAGAGGACAGCAGAAGAGGTT
CAGTTTGGCTGACTAAACCTTGTGTGCTTAACAGGAGCATGTAGCTTCTTCCTGCCTGAAATAGAAAGTAA
GAGCCTGTTGCTCTCCGTCTGGGGATAAAGGGAGAACGATTCACTTTCTGTGTTTGAAAATCCCTCTGTGTT
ACATGCCTAAGAAAGTTGCCTGTGCATGGTCTACCTGGATAATTTGTATGACTTATCCCCTTTCAAGTAGTT
GAAGCCGTTCAGGGTTTCCAGCATTTCGGGAGGCATTCTGTGTTGCGTGGCTGAGAATACAGCTTTACAGA
TGGTATCGCTAAAGGAGAAGAGGAACCGCGTTTCTCCCGTTCTGGAACACGAATGAGGCAAAACCGTAGTT
GTGCTGTGTAAAAATTTGCTGGCAGCAAGTGTAAGTTCCCA
>NW004444102.1_3
ATTCACCCCATGAAAGAGAAAGCAAAATACAGCGTTCTCCTTCAAGCTGTTTAACTGAATCTACTGAAGTT
TTCAGTTTTGTTAGAAGAAAGAGTCCGCACACTACAAGCTGCTTTCTCAAATGTTGTCTCAGTGGGAGAAG
GCAGCAGAGTGAAGGCCTAGCAAGAATTTGAATGCCCTGGTTTTCAAAGGGAACTTTGCATTACAGGAATG
AAGGTATTGCTAGCTTAGATAGCCAAACAGGCAGATTGTCTGCCTTCAGCTAGAGAACAGCAGAAGAGGTT
CAGTTTTGCTGACTAAACCTTGTGTGCTTAACACGAGCATGTACCTTCTTCCCAGCCTGAAAGAGAAACAA
GAGCTTGCTGCTCTCAGTTTGGGGATGAAGGGAAAAAGATTCCCTTTCTGTGTTTGAAAATCCCTCAGTGTT
ACATGCCTAAGAAAGTTGTCTGTGCATTGTCTACCTTGATAAAGTGTTTGACTTTTCTCCATTTGAAGTAGTT
GAAGCCGTTCAGGGTTTGCAGCATTCCGGGAGGCATTCTGTGCTGCGCGGCTGAGAATACCGCTTTACAGA
CGGTATCGCTAAAGAAGAAGAGAAACCGCGTTTCTGCCTTTCTGGAACACGAATGAGGCAAAAGCGTAGTT
GTGCTGCGTAAAAACTTTGTTAGCAGGAAGTGTAAGTTCCCA
>NW004444102.1_4
ATTCACCAAATGAAAAAGAAAGCAAAATACAGCGTTCTCCTTCAAGCTGCTTACTGAATCTACTGAAGTTT
TCAGTTTTGTTAGAAGAAAGAGTCAGCCCACCCCAAGCTGCTTTCTCAAATGTTCTCTCAGTGGGAGAAGG
CTGGTGAGTGCAGGCCTTGCAAGTATTTGAATCTCCTGATTTTCAAAGGGAACTTTACATACCAGGGAATG
AAGGTTCTAATGGCTTTCAAAGCCAAACAGGCAGATTGTCTGCCTTCAGCTAGAGGACAGCAGAAGAGGTA
CAGTTTGGCTGACTAAACCTTGTGTGCTTAACAGGAGCATGTAGCTTCTTCCCTGCCTGAAAGAGAAAGCA
AGAGCTTGTTGGTCTCAGTCTGGGGATGAAGGGAGAACGGTTCACTTTCTGTGTTTGAAAATCCCTCTGTGT
TACTTGCCTAAGAATGTTGCCTGTGCATTGTCTACCTCTGTAAAGTGTATGACTCTTCTGCGGTTCAAATAGT
TGATACCTTTCAGTGCTTACAGAGTTCCGGTAGGGAGTGTCTGCTGCCCAGCTGAGAATACTGCTTTACAGA
CGGTATCGCTAAAGGAGAAGAGAAACTGTGTTTCTGCTGTTCTGGAACACGAATAAGCCAGGCCACAGTTT
GGCTGAGTAAAAAGTATGTTTGTAGCAAGTATAAGTTACC
>NW004444102.1_5
ATTCAGTGCATGAAAGAGAAAGCAATATACAGCTTTCTCCTCAAGTTGCATAACTTCATCCACTGAAGTTTT
CAGTTTTGGTAGAAGAAACACTCCTCACAACACAAGCTGACTTCTCAAAGGTTCTCTCAGTGGGATAAGAC
TGGAGAGTGCCTGCCTTGCAAATACTTGAATCCCCTGCTTTTCAAAGGGAACTTTGCATAACAGGGAATCA
AGATACTTTTGGCATTCACAGCAAAACAGGCAGATTGCTTGCCTTCAGCTAGAAGATGGCAGAAGAGTTTC
AGTTTTGCTGACTAAAGATTGTGTGCTTAACGCGAGCATGTAGCTTCTTCCCTGTCAGAAACAGGAAGAAA
AGGCAGTTGCTGCTCTCAGTCTGGGGGTTGAAGTGAGAAGTTTTCCCTTACCTTATTTGAAAATCCCTCTAT
GTTACATGCCTAGGAATGTTGCCTGTGCATTGTCTACCTGGATAAAGGGTTTGATTTTTCTCCTGTTCGAGTA
GCTGAAGCCATGCGGGGTTTAAAGCTTTCCAGGAGTCATTCTGTGCTGTGCAGCCGAGAAAGCCGCCTTAC

AGACGGTATCGCTAAAGGAGAAGAGAAACGGCGTTTCTCCCGTTCTGGTGTAGGACAGAGGCAGCGCTGT
AGTCTTGCTGAGTGAAAGACTAAGTTAGCAGCAAGTGTAAGTTCCCA
>NW04444102.1_6
ATTCACTGCATGAAAAAGGAAGCAAAATACAGCGTTCTCCTTGACGCTGCTTAACTGCATCCACTGAAGTT
TTCAGTTTTGGTAGAAGAAACAGTCCACTCAACAGCAGCTGCTTTCTCAAAAGTTCTCTCAGTGAGATAAG
ACTTGAGAGTGCCTGCCTTGCAAGTACTTGAATCCCTTACTTATCAAAGGGAACTTTGCATAACAGGGAAT
CAAGCTACTTTTGGCATTCAAAGAAATACAAGCAGATTATTTGCCTTCAGCTAGAGGATGGCAGAAGAGGT
TCAGTTTTGCTGACTAAAGCTTGTGTGCTTAACGTGCGCATGTAGCCTCTTCCCTGCCAGAAAGAAGAAGCA
AAAGCCTGTTCTCTCAGTCTGGGGATGAAGTGCAAAGGATTCACCTACTGTGTTTGAAAATCCATCTGTGTT
ACATGCATAAGAATGTTGCCTGTGACTGTGTACCTGGGTAAAGGGTTGGATTTTTCTCCCGTTTGAGTAGCT
AAAGCCCTGCTGGATTTACAGCTTTAGGGAGGCATTCTACGTTGTTCAGCCGAGAACCCCGCCTTACAGAC
GGTATCTCTAAAGGAGAAGAGAAACCGTGCTTCTCCCATTCTGGTATGCAACAGCGGCAGCGGTGTAGTCT
TGCTGCGTAAAAGACTATGTCAGCAGCAAGTGTAAGTTGCCG
>NW004444102.1_7
ATTCACTGCATGAAAGAGAAAGCAAAATACAGCATGCTCCTTCAAGCTGCTTAACTTCTTCCACTGATGTTT
TCAGTTTTGGTAGAAGAAACAGTCCGTACACCACCAGATGACTTCTCAAATGTTCTCTCAGTGGGATAAGA
CTGGAGAGTGCCTGCCTTGCAAACACTTGAATCCCCTGGTTTTTAAAGGGAACTTTGCATAACAGGGAATC
AAGGTACTTTTGGCATTAAAAGCAAAACAGGCAGATGGTTTGCCTTCAGCTAGAGGATGGCAGAAGTGTTT
CAGTTTTGCTGACTGAAGCTTGTGTGCTTAACGCGAGCATGTAGCTTCTTCCCTGCCAGAAAGAGGAAGAA
AAGGCAGTTGCTGCTCTCAGTCTGACTGTTGAAGTGAGAAGGATTCACTTACTGTGTTTGAAATTCCCTGGG
TGTTACATGCCTAAGAATGTTGCCTCTGCATTTTCTACGTGAGTAAAGGGTTTAACTTTTCTCCCATTAGAGT
AGCTGAAGCCCTGCGGATTTTACAGCTTTCCGGGAGGCATTCTGTACTGTGCCGCTGAGAATGCCGCCTTAT
AGACAGTATCCCTAAAGGAGCGGATAAACCACATTTTTCCCGTTCTGTTATAGGACAGCGGCAGCGTTGTA
GTTTTGTTGAGTAAAGACTATGTTAGCAGCAAGTGTAAGTTCCTA
>NW004448733.1
ATTCACCACATGAAAGAGAAAACAAGCTACAGCGTTCTCCTTCAAGCTGCTTAACTGAATCTACTGAAGAT
TTCAGTTTTGTTAGAAGAAAGAGTCCGCCCAACAGAAGCTGCTTTCTCAAATGTTCTCTCAGTGGGAGAAG
GCTGGAGAGTGCAGGCCTAGCAAGTATTTGAATACCCTGGTTTTCAAAGCGAACTTTGCATAGCAAGGAAT
GAAGGTACTACTGGATTTCAAAGCCAAACAGGCAGATTGTCTGCCTTCAGCTAGAGGACAGCAGAAGAGG
TTCAGTTTGGCTGACTAAACCTTGTGTGCTTCACAGGAGCATGTAGCTTCTTCCCTGCCTGACAGAGAAAGC
GAGAGCTTGTTGCTCTCAGTCTGGGGATGAAGGGAGAACGATTCACTTTCTGTGTTTGAAAATACCTCTGTG
TTACATACCTAAGAAAGTTGCCTGTGCATTGTCTACCTGGATAAAGTGTATGACTTTTCCCCCTTTCAAGTC
GTTGAAGCTGTTCAGGGTTTCCAGCATTCCCGGAGGGATTCTGTGCTGCGCGGCTGGGAATACCACTTTACA
GAACGTATCGCTGAAAGAGAAGAGAAATCGCGTTTCTCCCCTTCTGCAACACGAATGAGGCAAAACCTTAG
TTGTGCTGTGTAAAACTTTGTTAGCAGCAAGTGTAAGTTCCCA
>NW004444141.1_1
GTTCACCACGTGAAAGAGAAAGCAAACTACAGCGTTCTCCTTCATGCTGCTTAACTGAATCTATTGAAGTT
GTCAGTTTTGTTCGAAGAAAGAGTCCCCCCAACACAAGCTGCTTTCTCTAATGTTCTCTCAGTGGGAGAAGG
CTGGAGAGTGCGGGCCTTGCAAGGATTACAATCCCCTGGATTTCAAAGGGAACTTTGCATTCCAGGGAATG
AAGATACTTCTGTCTTTCAAAGCCAACCATGGAGATTGTTTCCCTTCAGCGAGAGGACAGCAGAAGAGGTT
CAGTTTTGCTGACTAAACCTTGTGTTCTTAACACGAGCATGTAGGTTCCTCCCTGCCTGAAAGAGAAAGCAA
GAGCTTGTTGCTCTCAGTCTGGGGTTGAAGGGAAAAAGATTCACTTTCTGGGTTTAAAAATCCCTCTGTGTT
CCATGCCTAAGAAAGTTGCCTGTGCATTGTCTACCTGGATAAAGTGTATAACTTTTCTCCCTTTCAAGTAGT
TGAAGCCGTTCAGGGTTTCCAGCATTCCGGGAGGCATTGTGTGCTGCGCGTCTGAGAATAGCGCTTTACAG
GCAGTATCGCTAAAGGAGAAGAGAAATCGCGTTTCTGCCATTCTGGAGCACGAATGAGGCAAAACCGTATT
TGTGCTGAGGGAAACCTTTGTTAGCAGCAAGTGGAAGTTCCCA
>NW004444141.1_2
TTTCACCACGTGAAAGAGAAAGCAAACCACAGCCTTCTCCTTCGAGCTGCTTATCTGAATCTACTGAAGTTT
TTAGTTTTGTTAGAAGAAAGGGTCCCCCCAACACAAGCTGCTTTCTCTAATGTTCTCTCAGTGGGGAAGGCT
GGAGAGTGCAGGCCTAGCAAGTATTTCAATCCCCTGGATTTCAAAGGGAACTTTCCATTCCAGGGAATGAA
GGTATTTCTGTCATCCTAAGCCAAACAGGCAGATTGTTTGCCTTCAGCGAGAGGACAGCAGAAGAGGTTCA
GTTTTGCTGACTAAACCTTGTGTGCTTAACACGAGCATGTAGTTTCTTCCCTGCCTGAAAGAGAAAGCAAGA
GCTTGTTGCTCTCAGTCTGGGGATGAAGGGAGAACGATTCACTTTCTGTGTTTGAACATCCCTCTGTGTTAC
ATGCCTAAGAAAGTTGCGTGTGCATTGTCTACCTGGATAAAGTGTATGACTTTTCTCCCTTTCAAGTAGTTG
AAGCCGTTCTGGGTTTACAGCATTCCGGGAGGCTTTCTGTGCTGCGCGGCTGAGAATACCGCTTAACAGAC
GGTATTGCTAAAGCAGAAGAGAAACCGCGTTTCGCTATAGGACAGCGGCAGCGTGGTAGTTTTTTTGAGTA
AAAACTATGTTAGCAGCAGGTCTAAGTTCCCA

>NW004444141.1_3
AATCACTGCATGAAATAGAGAGCAAAAAACAGCGTTTTCCTTCAAGCTGCATAACTTCATCCACTGAAGTT
TTCAGTTTTGCTGGAAAAAAACTTCCACACACCACCAGCTGACCTCTCAAATGTTCTCTCAGTGGGATAAGA
CTGGTGAGTGTGTGCCGTGCAAGTACTTGAATCCCGTGGTTTTCAAAGGGATCTTTGCATTACAGGGGATGA
AATTACCTTTGCCATTGTAAGCAGAACGGTTAGATTGTTTGCCCTCAGCTAGAGGATGGCAGAAGTGGTTC
ACTTTTCCTGACTACATCTTGTGTGCTTAAAGCGAGCATGTAGCCTCTTCCCTGCCTGAAAGAGGAAGAAAA
GGCAGTTGCTGTCAGTCTGGAGGTGAAGTGAGAAGGATTCAATTACTGTGTTTGAAAATCCCTCTGAGTTA
CATGCCTAAGAAAGTTGCCTGTGCATTGTCTACCGGGGTAAGGGGTTTACTTTTCTCCTGTTCAAGTAGCTG
AAGCCCTGCGCGTTTTACCGCTTTCCTGGAAGCATTCTGTTCTGCGCGGCCGAGAATGCCGCCTTACAGACG
GTATCCCTGAAGGAGAAGAGAAACTGTGTTTCTCCCGTTCTGGTATAGGACAGCGGCAGCGTTGTAGTTTT
GCTGAGTAAAACTGTTTTAGCAGCAAGTGTAAGTTCCCA
>NW004444141.1_4
AATCACTGCATGAAAAAGAATGCAAAACAGTGTGTTCCCTTTCAAGCTGATTAACTTTGTCAACTGAAGTTT
TCAGTGTTAATAGAACAAACAGTCCTGACACCGCCAGCTACTTTCTGAAAATTTCTCTCAATGGGGAAAGA
CTGGGGATTGTCTGCCTTTCTAGTACTTGAATCCCCTGGTTTTGAAAGGGAATTTTGCGTTACAGGGAATGA
AGGAACTTTTCCCTTTCTAAGAAAAACAGTCAGATTGTTTGCCTTCAGCTAGAGGATGGCAGACGCGGTTC
AGTTTTGCAAACTAAAGCTTGTGTGGTTGACACGAGCATGTATCCTATTCACTGTAAGGAAGAGTAATGAA
AAAGTTGCTCTCAGTTTGGCGGATGAAGTGAGAAAGTCACTTACTGTGTTTGAAAATCTCTATGTGTTTCAT
GCCCAAAAATTGGCCTGTGCATTTTCTACCTGGGTGAAGATTTTGACTATTCTCCCGCTTGAGTACTTAAAG
CCCTGCGGGGTTTACAGCTTTCCTGGGAGGCACTCTGTGCTGCATGAACCAGAATGCCGCATTGCAGACGG
TACCCCTGAAGGAAAAGGGAAAGCATTGTTCTCGACTTCTGGTATACGACATCGGCAGCGTTGTAGTTTTG
CTAAGTAAACACAGTCTGTTAGCAGCATGTTCCG
>NW004444141.1_5
ATTCACTGCATGAAAAGAAAGAAAAATACAGGGTTCTCATTCAGGCTGGTTAAGTGAATCAACTGAAGTTT
TCAGTTTTGGTTGAACAAACAGTCCTGACACCACCTGCTACTTTCAGAAACATTCTCTCAATGGGGTAATAC
TGGTGAATGTCTGCCTTTCAAGTACTTGAATCCCCTCGTTTTCAAAGGGAACTTTGCATTACAGGGAATGAA
GTTAACTTTCGCATTCTAAGGAAAACAGGCAGATTGTCTTCAGCTAGACAACGACAGAAGCAGTTCAGTTT
TGCTGACTAAAGCTTGGTGGTTAACACGAGTATGTATCCTATTCCCTGCAAGAAAGAGGAAGAAAAAGACA
GTTGCTCTCAGTCTGGAAAATGAAGTGAGAAAATTCACTTACTGTGCTTGAAAATCTCTCTGTGTTAGATGC
CCAAGAAAGTGGCCTGTGCATTGCCTACCTCGGTAAAGGGTTTGACTTTTCTCCCATTCGAGTATGTGAAGC
CCTGCGGGGTCTACGGCTTTCCAGGAGGCAGTGTGTGCTGCGCACCCGAGAATGCCGCCTTACAGACGGTA
TCCCTGAAGAAGAAGATACAGCACGTTTCTCCCGTTCTGGTGTATGACAGCAGTAGAGATATAGTTTTGCT
AAGAAAACACTATGTTAGCAGGACCTGCATGTTCCT
>NW004444141.1_6
ATTCACTGCATGAAAGAGAAAACAAAATACAGTGTTCTCATTCAAGCTGGTTAACTTAATCAATTTGATTAT
CAATTTTGATAAAGCGCAGTCCTCACACCATCTACAAGCGGTCTCAAACATTCTCTCCATGGGATAAGATTT
GTGAGTTTCTCCGTTTCAAGTACTTGAATCCCCCGGTTTTCCAAGGAACTTTGCATTACAGGGAATGACTGT
TCCTTTCGCATTCTAGGAAGAAAAAGTGCAGATTTGTTCCCCTTCAGCTAAAGAACGGCAGAAGATTTTCA
GTTTTGCTGACGAAAACTGGTGTTGTAACCACGTGCGTGTTGCCTATTCACTGCAAGAAAGGGAATCCAAA
AGACAGTTGCTCTCAGTCTGGTACATGAAATGAGAATGATTTACTTACTGTGTTTGAGAATCTCTCTGTTTTT
CATGCCCAGGAAGGTCGCCTATGCATTCTCCACCTGGGTAAAGAGTTTGATTTTTCTCCTATCCGAGTGCGT
GAGCTCTGCGGGGTTTACAGTTTTCTGGGAGGCACTCCGTGTTGCACAGCCAAGAATACTGCCTTACAGGT
GGTATGTCTGAAGGAAAAAGCAAAGCACGTTTCTCCCCTTCTCCCCAGCAGTAGAGATGTAGTTTTGCTAA
GGAAACACTATGTTAGCAGAACCTGCATGTTCCCA
>NW004444141.1_7
ATTCACTGCAAGAAAGAGAAAGCAAAATACAGTGTTCTCATTCCTTAAACAAGTTGGTTTCCTTAAACAAG
TTGACTTATCCTTTTTGATAATACAGATTCCTGACAACATCTACAAGCTTTCTAAAACATTCCCTCAGTTGA
AAAGACTGGAGCATCTCCCCCTTTCAAGGTAGTTGAATCCCCCGGTTTTCAAAGGGAGCTTTCCCTTACTGG
GAATGACGGTATCTTTCATATCCTAGGAAGAACAAGGCAGATAATTTCGCCATCATATAAAGGACGACAGA
AGCGGTTCAGTTTTGCAGACTAAACTTTGTTTTGTAACCACTTGCATGTTCCCAATTCACTGCAAGAAAGAG
AAACCCAAATACAGTTTGTCTCATTCTAGTGGGGTAAATCAGAAGGATTAACTTAGTGTGACTGAAAATCC
TTCTGTGTTACATGCACAAAAATGTTGGCTCTACGTTCTCCACCTTGGTAAAGAGATGACCTTTTCTCCCATT
TGAGTACGTGAAGCCATGTGGGGTTTATGTGTTTTGAGTAGGCGCTCTGTCCTGCCCGGCCGAGAATTTGCC
TTACTGACTGTTTTTTGGAAAGAGAAAACAGTGTAAGTTTCTGCCTTTCAAGTATACGGCAGCAAGAGAGA
TGTCTGTTTGCTGAGGAAACACTTTGTGAGCAGAACATGCCTCTTCCC
>NW004444013.1_1
ATTCACCACATGAAAGAGAAAACAAGCCACAGCGTTCTCGTTCAAGCTGCTTAACTGAATTTACTGAAGTT
TTCAGTTTTGTTAAAAGAAAGAGTCCGCCCAACACAAGCTGCTTTCTCCAATGTTCTCTCAGAGGGAGAAG

GCTGGAGAGTGCAGGCCTAGCAAGTATTTGACTACCCTGGTTTTCAAAGCGAACTTGGCATAGCAGGGAAT
GAAGGTACCACTGGCTTTCAAAGCCCAACAGGCAGATTCTCTGTCTTCAGCTAGAGGACAGCAGAAGAGGT
TCAGTTTGGCCGATTAAACCTTGTGTGCTTCACAGGAGCATGTAGCTTCTTCCCTGCCTGACAGAGAAAGCA
AGAGCTTGTTGCTCTCAGACTGGGGATGAAGGGAGAACGATTCACTTTCTGTGTTTGAAAATCCCTCTGTGT
TACATGCCTTAGAAAGTTGCCTGTGCATTGTCTACCTGGATAAAGTGTATGACTTATGCCATTTCAAGTAGT
TGAAGCCGTTCAGGGTTTGCAGCATTCCGGGAGGCATTCTGTGCTGCGTGGCTGAGAATACAGCTTTAAAG
ATGGTATCGCTAAAGGAGAAGAGAAACCGCGTTTCTCCCATTCTGGAACACGAATGAGGCAAAACCGTAG
TTGTGCTGCGTAAAAACATTGAGCAGCAAGTAAATTCCCA
>NW004444013.1_2
ATTCACCACATGAAAGAGAAAGCAAAATACAGCGTTCTGCTTCAAGCTGCTTAACTAAATCTACTGAACTT
TTCAGTTTTGTGAGAAGAAAGAGTCCGCCCACCACAAGCTGCTTTCTCAAATGTTCTCTCAGTGGGAGAAG
GCTGGAGAGTGCAGGCCTAGCAAGTATTTGAATCCCCTGGTTTTTAAAGCAAACTATGCATAGCAGGGAAT
GAAGGTACTACTGGCTTTCAAAGCCAAACAGGCAGATTGTCTGCCTTCAGCGAGAGTATAGCAGAAGAGGT
TCAGTTTTGCTAATTAAACCTAGTGTGCTTCAAAGGAGCATGTAGCTTCTTCCCTGCCTGAAAGAGAAAGCA
AGAGCTTGTTGCTTTCAGTTTGTGGATGAAGGGAGAATGATTCACTTTCTGTGTTTGAAAATCCTTCTGTGTT
ACATGCCTAAGAGAGTTGCCTGTGCATTGTCTACCTGGATACAGCATATGACTTTTCAACCTTTCAAGTCGT
CGAAGCAGTTCAGGGCTTCCAGCATTCCGGGAGGCATTCTGTGCTGCGCGGCTGAGAATACTGCTTTACAG
ACGGTATCGCTAAAGGAGAAAAGAAACCGCGTTTCTCCCATTCTGGAACACGAGTATGAAGCTTCTTCACT
GCCTGAAAGAGAAAGCAAGAGCTTGCTGCTCTC
>NW004444013.1_3
ATTCACCACATGCAAGAGAAAGCAAAATACAGCCTTCTCCTTTAAGCTCCTTAACTGAGTCTACTGACGTTT
TTAGTTTTGTTAGAAGAAACAGTCCGCGGGACACAAGTTGTTTTCTCAAATGTTCTCTCAGTGGGAGAAGAC
TGGAGAGTGAGGGCCTAGCATCTATTTGAATCTCCTGGTTTTCAAAGGAAACTTTGCATAACAGGGAATAA
AGGTACTACTTGGTTTCAAAGCAAAACAGGCAGTTTGTTTGCTTTCAGCCAGAGGAGGGCAGAAGAGGTTC
AGTTTTGCTGACTAAACCTTGTGTGCTTAAGGCGAGCATGTAGCTTCTTCTCTGCCTGAAAGAGAAAGCAA
GAGCTTGTTTGTCTAAGTCGGGATGAAGGGAGAAGGATTCACTTACTGTGTTTGAATATCCCTCTCTGTTAC
ATGCCTAAGAAAGTTGCTTGTGCATTTTCTACCTGGATAAAGTGTATGACTTTTCTCCCTTTCAAGTAGTTGA
AGTCGTTCAGGTTTGACAGCCTTCCGGGAGGCATTCTGTGCTTCACGGCTGTGAAACCGCTTTACAGATCGT
ATCCCTAAAGGAGAAGAGAAACTGGTTTTTCTCCCGTTCTGGAACATGAATGAGGCAAAACCGTAGTTGTG
CTGAGTGAAAACGTTGTTAGCAGCAAGTGTAAGTTCCTA
>NW004444013.1_4
ATTCACCCCATGAAAGAGAAAGGAAAATACAGCGTTCTCCTTCAAGCTGCTTAACTGAATCTACCGAAATT
TTCAGTTTTGTTAGAAGAAACAGTCCGCCCACTACAAGCTGCTTTCTCAAATATTCTCTCAGTGGGAGAAAA
CAGGAGAGTGCCGGTCTAGCAGGTATTTCAATCCCCTGGTTTTCAAAGGGAACTTTGCATAACAGGGAATG
AAGGGTCTACAGGCTTTCAAAGCAAAACAGGCAGATTGTTTGCCTTCATCCAGAGGAGGGCAGAAGAGAT
TCAGTTTTGGTGACTAAACCTTGTGTGCTTAACACGAGCATGTAGCTTCTTCTCTGCCTGAAAGAGAAAGCA
AGAGCTTGTTGCTCTCAGTCTGGGGATGAAGGGAGAACGATTCACTTACTGTATTTGAAAATCCCTATGTGT
TATATGCCTAAGAAAGTTGCCTGTGTATCGTATACCTGGATAAAGTGTATGACATTTCTCCCTTTCAAGTAG
TTGAAGCCGTTCAGGGTTTCCAGCATTCTGGGAGGCATTCTGTGCTGCGCCGCTGAGAAGACCGCTTGACA
GACGGTATCCCTAAAGGAGAAGAGAAACTGCGTTTCTCCCGTTCTGGAACACGAATGAGGGAAAACCGTA
GTTGTGCTGAGTAAAAACTTTGTTAGCAGCAAGT
>NW004444013.1_5
ATTCACTGTATCGGAGAGAGAAAGCAAAATACAGCGTTCTCCTTCAAGCTGCTTAACTGAATCTACTGAAGTTT
TCACTTTTGGTAGAGAAACAGTCCGCCAAACACCAGCTGCTTTCTCAGAAGTTCTCTCAGTGGGAGAAGAC
TGGGAGTGCCTGCCTTGCAAGTATTTGAATCCCCTGGTTTTCAAAGGGAACGTTGCCTAACAGGGAATGAA
GGTAATTTTGGCATTCAAAGCAAAACAGACAGATTGTTTGCGTTCAGCTAGAGGAGGGCAGAAGATGTTCA
GTTTTGCTGACTAAACTTTGTGTGCTTAACACGAGTGTGTAGCTTCTCCAGTGCCAGAAGGAGAAAGCAAG
AGCCAGTTGCTCTCAGTCTGGGGAAGAAGTGAGAAGGATTCACTTACTTTGTTTGAAAATTTCTCTGTGTTA
CTTGCCTAAGAAAGTTGTCTGTGCATTGTCTACCTGGATAAAGTGTATGACTTTTCTCCCTTTCAAATAGTTG
AAGCCATTCGGGGTTTACAGCATTACGGGAGGCATTCTGTGCTTCGCGGCTGAGAATACCGCTTTACAGAC
GGTATCCCTATAGGAGAAGAGAAACCGCGTTTCTCCCGTTCTGGAACATGAACCAGGCAGAGGCGCAGTTT
TGCTGAGTAAAGACTGTGTTAGCAGCAAGTGTATGTTCCCA
>NW004444013.1_6
ATTCGTCGCATGAAAGAGAAAGCAACCTGCAGCATTCTCCTTCCAGCTGCTTAACTGAATCTACTGATGATT
TCAGTTTTGTTAGATGAAACACTCCGCCGACCACCAGCAGCTTTCTGAATCGTTCTCTCAGTGGGTAAGACT
GGATAGTGCCTGCTTTGCCAGTATTTGAATCCCCTGGTTTTCAAAGGGAACTTTGCTTTACAGGGAAAGGGG
TAACATTCGCATTCCAAGCAAAACAGGCAGATTGTTTGCTTTCAGCTAGAGGACTGCAGAAGTCGTTCAGT

TTTGCTGACTAAAGCTTGTGTGTTTAACACAAGCATGTATCCGATTCCCTGCAAGAAAGAGAAACCCAACG
ACAGTTTATCTCCCTCGGGGGGATGAAGTGAGTATGATTTACTTACTGTGTTTGAAAATCGCCTGTGTTACA
TCCTCAAGAATGTTGCCTGTGCATTGTTTAGCTGGGTAAAGACTTTGTCTTTTCTCCCTTTCGAGTACTTGAA
GCCCTGCTGTGTTTACAGCTTTCCAGCAGGCACTCTGTGCTGCTTGGCCTGCTGCCTTACAGAAGGTATTCC
TGAAGGAGGAGAGAAAGCAGGTTTCTCCTCTTGTGCTATAGGACAGCGGCAGGGCTGTAGTTTTGCTGACC
AAACTGTAT
>NW004444013.1_7
GTTCATCTAGGTAAGTTTTCAGTTTTGGTAGAACAAACAGTCCTCACACCATCAGCTGCTTTCTCAAACATT
CTCTCTATGGGAAAAGACTGGGAAGTGTCTGAATTTCAAGTTCTTGAATCCCCTGGTTTTCACAGGGAACTT
TGCCTTAAAGGGAATGAAGGTGACATTCGCATTTTAAGAAAAAAGGGCAGATTGTTTGCCTTCAGCTAGTG
GATGGCAGAAGCGGTTCAGTTTTGCTGACTAAATCTTGTGTGTTTGACCCGAGCATGTATCCGATTTCCTGC
AAGAAAGAGAAACCCAATGACAGTTGCTCTCCCTCTGGGGGATGAAGTGAGTAGGATAGACTTACTCTGTT
TGAAAATCCCCCGTGTTACATGCTCAAGAATGTTGCCTGAGCTTTGTTCACCTGGGTAAAGACTTTAACTTT
TCTGTCTTTAGAGTGCGTGAGGCCCTGCTATGTTTACAGCTTTCCGGGAGGTACTCTGTGCTGCGCGGCCGA
GAATGCCGCGTTACAGACGCTATCCCTGAAGGAGGAGAGAAAGGGCCTTTCTCCCCTTCTGCTACATGGCA
GTGGCAGTGCTGTCGTTTTGCTAACTAAACACTATGATAGCAACACATATATGTTCCCA
>NW004443969.1_1
ATTCAGCACATGAAAGAGAAAGCAAAATACAGCGTTCTCCTTCAAGCTGCTTAACTGAATCTACTAAAGTT
TTCAGTTTTGTTAGAAGAAACCGTCCTCCCACCACCAGCTGCTTTCTGAAACGTTCCCTCAGTGGGAGAAGA
CTGGAGAGTGCCTCCCTTGCAAGTATTTGAATCCCCTGGTTATCAAAGGGAACTTTGCATAAGAGGGAATG
AAGGTACCTTTGACATTCAAAGCCAAACAGTCAGATTATTTGCCTTCAGCTAGAAGATGGCAGAAGAGGTT
CAGTTTTGCTGACTAAACTTTGTGTGTGTAACGCGAGCATGAAGCTTCTTCCCTGCCAGAAAGAGAAAGCA
AGAGCCTGTTTCTCTCTTTCTGGGGATGAAGAGAGAAGGATTCAGGTACTGTGTTTGAAAATCCCTCTGTGT
TACATGCCAGAGAAATTTGCCTCTAAATTGTCTACCTGGATAAAGTGTATGACTTTTCTCCCTTTCAAGTAG
TTGAAGCCATTCAGGGTTTAAAGCATTCCGGGAGGCATTCTGTGCTGCCCAGCTGAGAACACCTCCTTACA
GCCGTTATCTCTAAAGGAGAAGAGAAACTGCGTTTCTCCCGTTCTGGAACAGAATGAGGCAGAGCCGCAGT
TGTCCTGAATAAAGGCTATGTTAACAGCAAGTGTAAGTTCGTA
>NW004443969.1_2
ATTCAGCGCATGAGGGAGAAAGGAATATACAGCGTTCTCCTTCAAGCTGCTTAACTGAATCTACTGAAGTT
TTCAGTTTTGTTAGAAGAAACTGTCCACCCACCACCAGCTGCTTTCTCAAACATTCTCTCAGTGGGAGAAGA
CTGCAGAGTGCCTGTCTTGCAAGTATTTGCATCCCTTGCTTTTCAAAGGGCACTTTGCATAAGAGGGAAAGA
AGGTAGTTTTCCCATTCAAAGACACAGGCAGATTGTTTGCCTTCAGCTAGAAGATGGCAGAAGAGGTTCTG
TTTTGCTGACTAAACCTTGTGTGTGTAACGCGAGCATGTAGCTTCTTCCCTGCCAGAAAGAGAAAGCAAGA
GCCAGTTGCTCTCAGTCTGGTGATGAATTGAGAAGGATTCACTTACTGTCTTTGAAAATCCCTCTGTGTTAC
ATGCCTAAGAAAGTTGCCTGTGCATTGTCTACCTGTATAAAGTGTATGACTTTTCTCCCTTTCAAGTAGTTG
AAGCTGTTCAGGGTTTGCAGCATTCTGGGAGGCATTCTGGGCTGCGCGGCTGAGAATACCGCTTTACAGAC
GGTATCACTAAAGGAGAAGAGAACTGCGTTTCTCCCATTCTGGAACACGAATGAGGCAAAAGCGTAGTTG
GGCTGAGTAAAAACTTTGTTAACAGCAAGTGTAAGTTCCCA
>NW004443969.1_3
ATTCACCACATGAAAGAGAAAGCAAAATACAGCGTTCTCCTTCAAGCTGCTTAACTGAATCTACTGAAGTT
TTCAGTTTTGTTAGAAGAAACTGTCCGCCCACCACCAGCTGCATTCTCAGATCAGGAAGAAAGCAGAGTTC
CTGCCTTGCAAGTATTTGAATCCCCTGGTTTTCAAAGAGAACTTTGTATAAGAGAGAATGAAGGTACTTTTG
CCATTCAAAGACAAACATTCAGATTGTTCCCCTTCAGCTAGAGGATGGCAGAAGAGGTTCAGTTTTGCTGA
CTAAACCTTTTGTGCTTAATGTGAGCATGTAGCTTCTTCTCTGCCAGAAAGAGAAAGCAATAGCCAGTTGCT
CTCACTCTGGGGATGAAGTGAGAAGGATTCACTTACTGTGTTTGAAAATCCCATCTGTGTTACTTGCCTAAG
AAAGATGCCTGTGCATTGTCTACCTGGAGAAAGTGTATGACTTTTGTCCCTTTCAAGTACTTGAAGCCATTC
AGGGTTTACAGCATTCCTGGACGCATTCTGTACTGCGTGGCTGAGAATACCGCTTTATAGACGTTATCGCTA
AAGGAGAAGAGAAAACGCGTTTCTCCCGTTTGGGAATATGAATCAGGCACAGCCGCTGTTTTCCTGAGTAA
AGGCTATGTTAGCAATAAGTGGAAGTTCC
>NW004443969.1_4
ATTCAGCACATGAAAGAAAGCAAAATACAGTTTTCTCCTTCAAGCTGCTTAACTGAATCTACTGAAGTTTTC
AGTTTGGTTAGAAGAAACTGCCCACCACCACCTGCTTTCTAAAAGGTCGTCTCAGTGACAGAAGTCTGCAG
AGTGCCTGCCTTGCAAGTATTTGAATCCCCTGGTTTTCAAAGCGAACTTTGCATACCAGGGAATGAAGGCTT
TCAAAGCCAAACAGGCAGATTGTCTGCCTTCAGCTAGAGGACAGCAGAAGAAGTTCAGTTTTGCTGACTAA
ACCTGTGTGCTTAATGTGAGCATATAGCTTCTTGCCTGGCAGAAAGAGAAAGCAAGAGCCAGTTACTCTCA
GTCTGGGGATGAAGTGAGTAGGATTCACTTACTGTGTTTGAAAATCCCTCTGTGTTACTTGCCTAAGAAAGT
TGCCTGTGCATTGTCTACCTGGATAAAGTGTATGACTTTTCTCCCTTTCAAGTAGTTGAAGCCATTCAGGGTT
TACAGCATTCCGTGAGGCATTCGGTACTACGTGGCTGAGTAGACCGCTTTACAGAAGGTATAGCTAAAGGA

GAAGAGAAACCACGTTTTTCCCGTTCGGAAATATGAATGGGGTAGAGGCGCCGTTTTCCTGAGTAAAGGCT
GTGTTAGCAGCAAGTTTAAGTTCCCA
>NW004443969.1_5
ATTCACCGCATGAAAGAGAAAGCAAAATACAGCGTTCTCCTTCAAGCTGCTTAACATAGTCCATTGAACTT
TTCAGTTACATTAGAAGAAACAGTCCGCCCACCACCAGCTGCTTACTCATAGGTTCTCTGTGATGGATAAG
ACTGGGGAGTGCTTGCCTTGGAAGTATTTAAATCCCCTGATTTTTGAAGGGAACTTTGCAAAACAGGAAAT
GAAAGTACATTTGGTATTGAAAGCAAAACAGATTGTTTGCCTTCAGCTAAAGGATGGCAGAAGAGGTTCAG
TTTTGCTGACTAAATCTTGTGTGCTTAATGTGAGCCTGTAGCCTCTTCCCTGCCAGAAAGAGGAAGCAAAGC
CAGTTGCTCTCAGTCTGGGGGACGAAGTGAGAAGGATTCACTTACTGTGTTTGAAAATCGCTCTGTGTTACA
TGCCTAAGAAAGTTGCCTGGGCATTGTCTACCTGGGTAAAGGGATCCACTTTTCTCCCTTTCAAGTCGGTGA
AACCATTCAGTGTTTACAACATTCCGGGAGGCATTCTGTGTTGCGCGGCCGAGAAAGCCGCTCTGCAGATG
GTATCTCTAAGGGAGAAGAGAAACCGCTTTTCTCCAGTTCTGGTATAGGACAGTGGCAGAGCAGCAGTTTT
GAGTGTGTTAGCAGCAAGTGTAGGTTCCCA
>NW004443969.1_6
CTTCACTGCATGAAAGAGAAAACAAAATACAGTGTTTTCCTTCAAGCTGCTTAACTCAATCTACTGAAGTTT
TCAGTTTTGTTAGAAGAAACAGTCCGCCCGCCACCAGCAGCTTTCTCAAAGGTTGTCTCAGTGGGATAAGA
CTGGAGAGTGCCTGCCTTGCACATATTTGAATCCCTGGTTTTCAAAGGGAACTTTGCATAACAGACAATGA
AATTACCTTTGGCATTCAAAGCAAAACAGGCACATTGTTTGTCTTCAGCGGGAGGATGGTGGACGAGGTTC
AGTTTTCCTGACTAAACCTTGTGTGCTTAACGCGAGCATGTAGCTTCTTCCGTGCCAGAAAGAGAAAGCAA
GAGTCAGTTGCTCTCATTCTGGGGGATGCAGTGAGAAGGATTCACTTACTGTGTTTGAAAATCGCTCTGTGT
TATATGCCTAAGAAAGTTGCCTGGGGATTTTCTACCTGGGTAAAGGGATCCAGTTTTCTCCCTTTCAAGTAG
GTGAAGCTGTTCAGCATTTACAGCTTTCCGGGAGGCATTCTGTGCTGCGTGGCCAGGAATGCCGCCTTGCA
GACATTATCCCTGAAGGAGAAGAAAAACTGCATTTCTCAGGTTCTGGTATATGACAGCGGCACAGCAGCAG
TTTTGCTGAGTAAAGACTATGTTAGCAGCAAGCGTAAGTTCCCA
>NW004443969.1_7
CTTCACCGCATGAAAGAGAAAGCAAACTACAGCATTCTCCTTAAGCTGCTTAACTTCATCCACTGAAGTTTT
CAGTTTTGTTAGAAGAAACAATCCGCTCAGCACCAGCTGCTTACTCAAAGTTTCTCTCAGTGGTATAAGACT
AGAGAGTGCCTGCCCTGCAATTATTTGAATCCCCTGGTTTTCAAAGGGAACTTTGCCTAACAGGGAATGAA
GGTACCTTTGGCATTCAAAGCAAAACAGGCAGATTGTTTGCCTTCAGCTAGAGGATGGAAGAAAAGGTTCA
GTTTTGCTGAGTAAAGCTTGTGTGCTTAACCTGAGCCTGTAGCCTCTTCCCTGCCAGAAAGAGGAAGCAAA
GCCAGTTGCTCTCAGTCTGAGGGATGAAGTGAGAGGGATTCACTTACTGTGTTTGAGAATCGCTCTGTGTTA
CATGCCCAGGAATGCTGCTTGTGCATTCTCTTCCTGGAGAAAGATTTTGAGTTTTCTCCCTTGGACTTCTTGA
ATCCCTGTGGCTTTTGCAGCTTTCTGGGAGGCGCTCTTCGCTGCATGGGCAAGAAAGCAGACTTTTCAGGCG
GCATCTCTGAAGGAGAAAACAAAGTGCCTTTCTCCCGTTCTGGTATACGACAGCAGTAGAGATATAGTATC
TCTAAGCAAACTCTATGTTAGCAGGACTTGTATGTTCCCA
>NW004443969.1_11
ATTCACTGCATAAAAGAGAAAGCAGAATACCGTTTCTCTCACTCGGCTGAAGTCAGTCAGAAGGAATAACA
TTCTGTGTTTGAAAATCCCTCTGTGTTACATGCAAGAGAGTGCTGCCTAAACTTTCTCTATGGTTTTAAAGTG
TTGAACTTTTCTCCCATTCAAATATGTGAAGTCATATGGGGTATAGGTGTGTTGAAGCCGTACTCTGTGCTG
TCTGTTTGTGAATGTGCCTTACAGACTGAATCTTTGAAGGAGAAAACAATGCTTCTTTTCCCATTCAAGTAC
AGGACGACAATAGAGATGTAGTTTTGCTAAGCAAACACTATGTTACCAGAACCTGCATATTGTTCACAATT
CACTGCATTAAAGAGAAAGTAAAATAGAGTTTCCATCACTTGGCTTGAGTAAGTCAGAAGGAATAACTAAC
TGTGTTTGAATACCCCTCTGCGTTACATGCAAAAGATGATTGCATATACATTCTCTACGGGGGTAAAGTTTT
GAAGTTTTCTTCCATTCGAATACATGAAGCCATATGGAGTATAGATGTATTGAAGCAGTACTCTATGATGTT
CTTTCCTGAATGTGACTTACACACCGAATCTCTGAAGGAGAAAACAATGCGTGTTTCCTCGTTCCAGTACAG
GACTGCAACAGAGATGTAGATTCGCTAAGGAAACACTGTGTTACAAGAACGTGCATGTTC
>NW004443969.1_12
ATTCACTGCTTGAAAGAGAAGCAAAAATACTGTTTCTTTAATAGTGTTTGAGTAAGTCAGAAGGATGAACT
TACTGTGTTTGAAAACCCCTCTGTGTTACACGCACGACAGTGTTGCCTATACGTTCTCTATCAGGGTAAAGT
GTTCAAATTTTCTCCCATTCAAATACATGAAGCTGTAAAGGGTTTAGATGTTCTCAAGCAGTACTCTGTGCT
GCCCATTCCTGAATGTGCCTTAAAGACAGAATCTCTGAAGGAGAAAACAAGGTGTGTTTCTCTGGTTCAAG
TACAGGACGGCAATAGAGATGTAGTTTCCCTAAGTAAACACTGTGTTACCAGAACATGCATGTTCCCAATT
AACTGCATGAAAGAGAAAGCAAAATCCAGTTTCTTTCACTTGGCTTGACTAAGTCAGAAGGATTAACTTAC
TGTGTTTGAAAACCCCTCTGTGTTACATACACGAGAGTGTTGCCTACACATTCTGTACCAGGTAAAGTGTTA
AACTTTTCTCCCATTCAAATATATGAAAGTCTTTGGGGTTTTTGATGTTTTCCAGCAGTACTCCATGCTGCCC
ATTCATTGATGTGCCTTACAGACAGACTCTCTGAAGGGGAACACAATGCGTGTTTCTCTTATTCAAGTACAG
GACGGTAATAGAGATGTGGTTTTGTTAAGACTGTGTTACCAGAACCTGCATGTTCCCA

>NW004443969.1_13
ATTCACCGTATGAAGGAAAACCTAAAATACAGGTTTCTCAGTCTAGTTGTTTAACTTAATCAAGTTGATTAA
GGTATCTGTTTGGTAAAACACAGTAGACACAGGATTGAAAACTTTTCTTACACATTCTCTGAATGGTAAAA
GAATGTCAAGTTTCTCCCTTTCTTGTTCTTGAAGCCTCAGCAGTTTCAGTTGTTTTCCATAGAAACTCTGTGT
TACTGTGACTGAACGTGCCTTTCTCATTCTAGGAAAAACAAGGCAGGTTGTTTCCCGTTCTACTACAAGATG
GCAAAAGTCTTGTAGTTTTGTTGAGTATACCTTCTGTTGTAAGCACGTGCATGTTTCCAGTTTACTGCACAA
AAAAGAAAGCAAAATACAGTTTCTCTCATTCTGCTTGACTAAGTCAGAAGGATTAACTTATTGTGTTTGAAA
ACCATCTGTGTTACATGCAAGAGAGTGTTGCTTATACATTCTCTACCAGGGTAAAGTGTCTAACTTTTCTCC
CATTCAAGTAGGTGACGCCATGCGGGGTTTAGATGTCTTCAAGATGCACTTTGTGCTGCCCGGCCTTGAATA
TGCTGAATGAGAAAACAATGCAAGTTTCTCCCAGTCAAGCATAGGACAGCAACAGAGATATACTTTTGCTA
AGTTAACACTGTGTTACCACAACACACGTTCCCA
>NW004443969.1_14
TTTCACTGCATTGAAGAGAAACCAAAATACAGGTTTCTTATTCTAGCTGATTAACTTAATCAAGCTGGTTAT
CTGTTTTTATAAAACATTGTTACCACACGATGGGAAACCTTTCTTGCACATTCTCTGAATGGGAAAAAAAAT
ATTAAGTTTCTCCCTTTCTGATACGTGAAGCCCCCGGGCTTTCAGATGTTTTCTATGGGAGCTCTGGTTTACT
GGGAAAGAATGTGCCTTTCACATTCTAGAAAGAACAAGGCTGATTGTTTCCCATTCAACTACAAAACGGCA
AAAGTGATGTGGTTTTGCTGACTAAGCCTTGTGTTGTAAGCACGTGCATGTTTCCAATTCACTCCACGAAAG
AGAAAGCAAAACACAGCTTCTCTCATTCTGCTTGAGTAAGTCAGAGGAATTAACTTACTGTGTTTGAAAAC
CCCTCTATGTTACATGCATGAGAGTGTTGCCTATACATTCTCAGCATAGTAAAGGGTATAACTTTAATCTCA
TTCAAATACATGAAGTCATACTGGATTTAGATGTTATCAAGCAGTACTATGTGCTACCCTTTTGTGAATGTG
CCTTACAGACAAAATCTCTGAAGGAGAAAACAAAGCGAGTTTCTCCCCGTGAAGTATAGGACAACAATAG
AGATGTAGTTTCCCTAGGTAAACACTGTGTTACCAGAACGTGCATATTCCCA
>NW004443969.1_15
ATTCACTGCATCAAAGAAAAACCAAAAGAAAGGTTCTTCGGTCTAGTTGATTAACTTAATCAAGTTGATTA
AGTTACCTGTTTTGATAAAACACTGTTGCCACACGTTTAAAAACACTTCTTACACATTCTCTGAATGGGAAA
AGAATGTCAAGCTTCTCCCTTTCCATTACCTGAAGCCCCCAGGCTTTCAGATGTTTTTATGGGATCTCTGGT
TTACCAGTGATGAATGTGCCTTTCATATTCTAGGAAAAACAAGGCTGATTGTTTCCCATTCAACTACAGAAT
GGCAAAAGCTATGTGGTTTTGCTGACTAAGCCTTGTGTTTTATGCACATGCATGTTTCTAATTCATTGCTCAA
AAGAGAAACCAAAATACTATTTCTTTCACAGTCACTGAGTAAGTCATAAGGATTAAGTTACCGTGTTTGAA
AACCCCTCTGTGTTACATGCACAAGAATGTTGCCTATACTTTCTGTACCAGAGTAACGGGTATAATTTTAAT
CCCATTAAATATGTGAAGTCATACGGGATTTAGACATTTTCAAGCAGTATTCTGTGCTGCCCATTCTTGAAT
GTGCCTTACAGACAGTATCTCTGAAGGAGAAAACAATGCGAGTTTCTCCTCGTGAAGTACAGAACAATACA
GATGCAGTTTCCCTAAGTAAACACTGTGTTACCAGAATGTGCATATTCCCA
>NW004443980.1_1
ATTCACTGGATGAAAGAGAAAGCAAAATACAGTGTTTTCATTCAAGCTGGTTAAGTTAATCAACTTAATTA
AGTTATCAGTTTGATAAAACACAGTCCTTACACCATCTACAAGATCTCTCAAACATTCTTTCAATGACATAA
GACTGGAGAGTTTCTCCCTTTCAGGTACTTGAATCCGCCGGTTTTCAAATGGAACATTGCACTACAGAGAAT
GACGGTTCCTTTCACATTCTAGGAAGAAAAAGGGCAGATTGGTTCCCTTTCAGCTAAAGAGCAGCAGAAGC
TGTTCAATTTTGCTGACTACACCTTGTGTTGCAACCACGTTTATCTTACTTATTCAGTGGAAAAAGGCAATTC
AAAAGACACTTGCTCTCAGTCTGACGGATGGAGAAGGATTTACTTACTGTGTTTGAAAATCCCTCTGTGTTA
CATCGCCAGGAAGGTTGACTATGAATTCTCCACCTGGGTAAAGAGTTTTTTTTCTCCCTTCCGAGTACATGA
AGCCTTGCGCTGTTTACAGCTTTCTGGGAGGCACTCTGTGCTGCGAGGCTGAGAATGCTGCCTTACAGAAG
GTATGACTGAAGACAAAAACAAAGCGCGTTTCTTCCGTTTTGGTACACTACAATAGTGGAAATGTAGTTTTT
CTAAGGAAACACTATGTTAGCAGGCCCTGTATGTTCCCA
>NW004443980.1_2
GTTCACTGCATGAAATAGAAAGCAAAATACAGTGTTCTCATTCAAGCTGGTTAACTTGATTAAGTGATCAA
TTTTGATAAAACACAGTCCTCACAGCATCTACAAGCTCTCTCAAACATTCTCTCAATGACATAAGACAGGTG
AATTTCTCCCTTTCAAGTATTTGAATCCCCTGGTTTTCAAATGGAACTTTTCATTACAGAGAAGGAAGGTGT
TTTCTGCATTCTAGAAATAAAAAGGGCCGATTGCTGCCCCTTAGCTAAAGAGCCGCAAAAGTGGTTCAGAT
TTGCTGACTAAACCTTGGGTTGTAACCATGTGCATGTTCCCTTTTCAATGCGAGAAAGAGAATCCAAAGGA
CAGTTGCTGTCAGTCTGGTGGATGAAGTGAGAGTGATTTACTCACTGTTTGAAATCTGTCTGTGTTACATGC
CCAGGAACATCATCTATGCATTCTCTACATGGGAAAAGAGTTTGACTTTTCTCCTTTCCAAGAACGTGAAGC
CAAGCGCAGTTTATAACCTTCTGGGAGGCACTCTGTGCTGTGAAGGCCACAATGCTGCCTTATAGACAGTA
TGTCTGAAGACGAAAACAGAGCGTGTTTCTCCCATTCTGGTATATGACACCACTAGAGAGGTAGTTTTGCT
AACGGAACACTATGGTAGCAGGCCCTGCAAGTTCCCA
>NW004443980.1_3
ATTCACTGCATGAAAAAGAAAGCAAAATACAGTGTTCTCATTCAAGCTGGCTAACTTAATCCACTTAAACT
ATCAATTTTGATAAAAGATAGACCTCAAACCATCTACAAGCTCTCTCAAACATTCCTCAATGGGATAAGAC

TGGTGAGTTGCTCCCTTTCAAATGCTTCAATCTCCCGCTTTTCAAAGGGAACTTTGCATTATATGAACTGAA
GGTACTTTTCACATTCAAAGAAAACAAGGCAGATTGGTTCATCTTCAGATTAAAGACAACAGAAGCGGTTC
AGTTTTGCTGATTAAACCGTATGTTGTAACCATGTGCATATTTCCTATGCACTGAAAGAAAGAGAAACCTAA
AGAACCTTGCTCTTCATCTGGTGGATGAAGTGAAAAGGATTCATTTACTGTGGTTGAAACTCCCTCTGTGTT
ACATGCCCAAGAATGCATGGTCTACCTGGGTAAGAGTTTGACTTTTCTCCCCTCAGAGATTGTGAAGCTCTG
CAGGGTTTACAGCTTTCCAGGAGTCGTTCTGTGCTGCACAGCCGAGAATGCAGCCTTACAGACGGTCTCCCT
AAAGGAGAAGAGAAAGTGTGTTTCTCCCGTTCTGGTATATGACAGTGGCAGTGCCGCAGTTTTGCAGAGTA
AAGACTATGTTAGCAGCAAGTGTAAGTTCCCA
>NW004443980.1_4
TTCACTGCCTGAAAGAGACAGCAAAAATGCAGCATTCTCCTTCAATCTACTAAACGTAATCCACTGAAGTT
TTCTGTTTTGGTAGAAGAAACAGTCCACACAGCACTAGCTGACTTCTCAAACGTTCTCTCAGTGGGATAAG
ACTGGAGAGTGCCTGCCTTGCAAGTATTTGAATCCTCTGCTTTTCAAAGGGAACTTCGGATAACAGGGAAT
GAATATACCTTTGGCACTCAAAGCAAACATACAGATTGTTTGCCTACAGGTAGAGGATACCAGAAGAGGTT
CAGTTTTGCTGACTAAACCTTGTGTGCTTAACGCAAGCATGTAGCCTCTTCTCTACCAGAAAGAGGAAGCAT
AACCCAGTTGCTGTCAGTGTGGGGGATGAAGTGAGAAGGAGTCAATTACTGTGTTTGAAAATCCCTCTGTG
TTATACACCTAAGAAAGTTGCCTGTACATTGTCTACCTGGATAAAGTGTTTGACTTTTCTCCCGTTCGAGTA
GCTGAAGCCCTGTGGGGTTTACAGCTTTCAGGGAAGATTTCGGTGCTACGCGGCCGAGAATGCCACCTTAC
AGGCGTTATTGCTGATGGAGAAGAGAAACCGCGTTTGTCCCCTTCTGGTATAGGACCGAGGCAGAGTCGCA
GTTTTGCTGAGTAAAGACTATGTTAGCAGCAAGTGTAAGTTCCCA
>NW004444509.1_1
ATTCACCACATGCAAGAGAAAGCAAAATACAGAGTTCTCCTTCAAGCTGCTTAACTCATTCTACTGAAGTTT
TTAGTTCTGTTACCAGAAACAGTCCGCGCACCACAAGCTGCTTTCTCAAAAGTTCTCTCAGAGGGAGAAGA
CTGGAGAGTGCGGGCCTAGCAAGTATTTGAATCTCCTCCTTTTCAAAGGAAACTTTGCATAACAGGGAATG
AAAGTACTACTGGCTTTCAAGGCAAAACAGGCAGATTGTTTGCCTTCAGCCGGAGGAGGGCAGAAGAGGT
TCAGTCTCGCTGACTAAACCTTGTGTGCTTAACGCTAGCATGTAGCTTCCTTCTTGCCTGAGGGAGAAAGCA
AGATCTTGTTGCTCTCAGGCTGGGGATGAAGGGAGAACGATTCACTTACTGTGTTTGAAAATCCCTTGGTGT
TACATGCCTAAGAATGCTGCCTGTGCATTTTCTACCTGGATAAAGTGTATGACTTTTCTCCCTTTCAAGTAGT
TGAAGCCGTTCAGGGTTTCCAGGATTTGGTAGGCATTCTTTGCTGCACGGCTGAGAATACCGCTTTACAGAA
GGTATCACTAAAGAAGAAGAGAAACTGCATTTCTCCCATTCTGGAACACGAATGAGGCGAAACCGTAGTTG
TGCTGAGTAAAAACTTTGTTAGCAGCAAGTGTAAGTTGCCA
>NW004444509.1_2
ATTCACTGCCTGAAAGAGAAAGCAAAATACGGCGTTCTCCTTCAGCTGCTTAACTGAATCTACTGAAGTTTT
CAGTTTTGGTAGAAGAAACACTCTGCCCACCACCAGTTGCTTTCTCAAAGATCTCTCAGTGGGAGAAGACT
GGAGAGTGCCTGCCTTGCTGGTATTTGAATCCCCTGGTTTTCAAAGGGAACTTTGCATTACAGGGAATGAA
GGTACTTTTGGCATTCAAAGCTAAACAGGTACATTGTTTCCCTTCTGCTAGAGGACAGCAGAAGAAGTTCA
GTTTTAATGAATAAAATATGTGTGGTTAACGGGAGCATGTAGTTTCTTCCATATCAGAAACAGAAAGCTAG
AGCCAGTTTCTCTCGGTGTGGGGATGAAGTGAAAAGGATTCACTTATTGAGTTTGAAAATCCCTCTGTGTTA
CTTGACTAAGAAAGTTGCCCGTGCATTGTCTACCTGGAGAAAGTGTATGACCTTTCTCCCTTTCAAGCAGTT
GTAGCCTTTCAGGGTTCATAGCCTTCCGGGAGGCATTCTGTGCTGCGCGGCTGAGAATACCGCTTTACAGAC
GTTATCCCTAAAGGAGAAGAGAAACCGAGTTTCTCCCTTTCAGGAACATGAATGAGGCAGAGCCGCAGTTT
TGCTGGGTAAACACTATGTTAGCCGCAAGTGTTTAAGTTCCCA
>NW004444509.1_3
ATTCACTGCATGAAAGAGAAAGCAAAATACAGCGTTCTCCTTCAAGCTGCTTAACTGAATCTACTGAAGTT
TTCAGTTTTGTTGGAAGATAGAGCCCCCCCACCACAAGCTGCTTTCTCAAATGTTCTCTCAGTGGGAGAAGG
CAGTAGAGTCAAGGCCTAGCAAGAATTTGAATCCCCTGGTTTTCAAAGGGAACTTTGCATAACAGGGAATG
AAGGTACTCTTGGCATTCAAAGCAAAACAGGCACATTTTTTCCCTTCTGCTAGAGGATGGAAGAAGCTGTT
TAGTTTTCCTGATGAATTTTCAGTGCTTAACACAAGCATGGAGTTTCTTCCATATCAGAAAGAGAAAGCGAG
AGCCAGTTTCTCTTGGTGTGGGGATGAAGTGAAAAGGATTCACTTATTGAGTTTGAAAATCCTTCTGTGTTA
AATGCCTAAGAAAGTTGCCTGTGCATTGTCTACGTGGACAAAGTGTATGACTTTTCTCCCTTTCAAGTAGTT
GAAGCCATTCAGGGTTCATAGCCTTCCGGGAGGCATTCTGTGCTGCGCAGCTGAGAATGCCCCTTTACAGA
CGTTATCCCTAAAGGAGAAGAGAAACCGAGTTTCTCCCATTCAGGAACATGAATGAGGCAGAGCCGCAGTT
TTGCTGGGTAAACACTATGTTAGCAGCAGGTGTAAGTTCCCA
>NW004444509.1_4
ATTCACTGCATGAAAGAGAAAGCAAAATACAGCGTTCTCCTTCAAGCTGCTTAACTGAATCTACTGAAGTT
TTCAGTTTTGGTAGAAGAAACACTCCGCCCACCACCAGCTGCTTTCTCAAAGGTTCTCTCAGTGGAAGGAG
ACTGGAGAGTGCCTGCCTTGCCAGTATTTGAATCCCCTGGTTTTCAAAGGGAAAGTTCAATTACAGGGAAT
GAAGGTACTCTTGGCATTCAAAGCAAAACAGGCACATTGTTTCCCTTCTGCTAGATGATGGCAGAAGAGTT

TCAGGCGTGCTGAATAAAGTTTGTGTGCTTAACGCGAGTATGTAGCTTCTTCCATGTCAGAGAGAGAAAGC
GGAGCGTTTCTTTCGGTCTTGGGATGAAGTGAGAAGGTTTCACTTACTGTGTCTGAGAATCCCTCTGTGTTA
CTTGCCTAAGAAAGTTGCCTGTGCATTGTCTACCTGGATAAAGTGTATGTCTTTCTTCCTTTCAAGTAGTTGT
AGCCATTCAGGGTTCATAGCCTTCCGAGAGGCATTCTATGCTGCGCGGATGAGAATACCGCTTTACAGAAG
GTATCCCTAAAGGAGAAGAGAAACCACGTTTCTCTTGTTCTGGAACACGAATGAGGCAGAGCCGCAGTTTT
TCTGAGTAAAGACTATGTTAGCAGCAAGTGCAAGTTCCCA
>NW004444509.1_5
ATTCACTGCATGAAAGAGAAAGCAAAATACAGCGTTCTCCTTCAAGCTGCTTAACTGAATCTACTGACGTT
TTCGGTTTTGGTAGAAGAAACACACCGCCCACTACCAGCTGCTTTCTCAAAGGTTTTCTAAGTGGAAGTAGC
TTCTAGAGTGCCTGCCTTGCCAGTATTTGAATCCCCTGGTTTTCAAAGGGAACGTAGCATAACAGGGAATG
AAGGTACTTTTGGCATTCAAAGAAAAACAAGCAGATTGTTTCCCTTCTGCTAGGGGATGGCAGAAGAGGTT
CATTTTTGATGAATAAAGTTTGAGTGCTTAACGCGAGCATGTAGCTTCTTCCATGTCAGAAAGAGAAAGCG
AGAGCCAGCTTGTCTCGGTCTGGGTATGATGTGAAAAGGATTAACTTACTGTGTTTGACAATCCCTCTGTGT
TACTTGCCTAAGAAAGTTGCCCGTGCATTGTCTACCTGGACAATGTGTATGACTTTTCTCCCTTTCAAGTAG
TTGTAGCCATTCAGGGTTCATAGCCTTCCGAGAGGCATTCTATGCTGCGTGGAGGAGAATACTGCTTTACAG
AAGGTATCCCTAAAAGAGAAGAGAAACCACGTTTCTCCCGTTCTGGAACACGAATGAGGCAGAGCCGCAG
TTTTCCTGAGTAAAGACTATGTTAGCAGCAAGTGCAAGTTCCCA
>NW004444509.1_6
ATTCACTGCATGAAAGAGAAAGCAAAATACAGCGTTCTCCTTCATGCTGCTTGACTGAATCTACTCAAGTTT
TCAGTTTTGGTAGAAGAAACAGTCCTGACACCACCAGCTACTTTCTGAAACATTCTCTCAATAGGATAAAA
CTGACGAGTGTCTGCCTTCCAAGTACTTGAATCTCCTGGTTTTCAAATGGAGATCTAAATTACAGGGAGTGA
AGGAACATTTCTCATTCTAAGGAAAGGAGGCAGATTGTTTGCCTTCACCTACAGGACAGCAGAAGCAGTTC
AGTTTTACTCACTGAAGCTTGGGTGCTTAACATGAGCATGTATCCTATTTCCTTCAAGAAAGAGAAAGCAA
AAGACAGTTGCTGTTAGTATGGGAAATGAAGTTAGAAAGATTCACTTACTGTGTTTCAAAATCCCTCTGTTT
TATGTGCCCAAGTAAGTGGCCTGTGCATTGTCTACCTGGGTGAAGAGTTTTCCTTTTCTCCCGTTCGAGTAT
GTGAAGCCCTGCATGGTTTACAGCTTTCCCAGAGGCTCTTTGTGCTGCGGTGCCGAGAATACCCCCTTTGAA
AGAGAAGAGAAAGCGTGTTTCTCCAGTTCTGGTATATGACAGCAGCTGCACTGTAGTTCTGCTAACCAAAC
ACTATGTTAGCAGCACGTGCATGTTCCCA
>NW004444509.1_7
GTTCACTGCATAAAAGTGAAAGCAAAATACAGTGTTCTCATTCATGCTGGTTAAATTAATCAAGTTGATTAA
TTTATCTGTTTTGAAAAACGGAGTTCCTACTTGGTGAAAACCTCTCTTACACGTTTTCTGAATGGAAAACAA
TGTCAGGCTTCTCCCTTTCAAGTACTTTAAGTCCCTGTGTTTTCACTTGTTTTGTTTCTAAATTTTAGGTCACT
CGCAATGAATGTGCCTTTCAAATTCTATGAAGAACAAGGCGGTGTGTTTCCCTTTGAACTACAGCAAGGCA
AAAATGATGTAGTTTTCCTGAATAAACCTTGTGGTGCAAGCACTGGGTAACTTTGCACTTCTCTGAACTAAA
GAGAAACCAAAAGACAGTTTCTGTCATTGTGCTTGAGAAAGTCTGAAGGATTTACTTACTGTGTTTGAACA
CCCCTCTCTGCTACATGCAGGGGAAATTGCCTTTACCTTCTCAACCAGAAAAAAGTATTTTGCTTTTCTGCC
ATCCAAATACTTGAAGCCATACGCGGTTTAGCTGTTTTTAAGATGCACTTGAGCTGCCCGACCGTGAATGG
GCTTTACAGACAGAATCTCTGAAAGAGAATGCACTGCGAGGTTCCCCCAGTCAAGTATAGGATGGCTATAG
AGATGTAGTTTTCGAAGCAAACACTGTGTTAGTGCAACTTGCATGTTCTC
>NW004444509.1_8
ACTCACTGCCTGGAAGAGATGCAAAATACAGTGTAGTCACTCTAGTTGGTTAACTTAATCACATTGATTAA
GTTATCTGTTTTGAAAAACACAGTTCCTACATGGTGAAAACCTTTCTTACACATTGTCTGAATGGGAAAACA
ATGTCAAGCTTCTCTTTTTCAAGTACTTGAAGCCACCGGGTTTTCACATGTTTTCTGTCTAAATTTTAGGTTA
CTGGCCATGAATTTGCCTTTCAAATTCTAGAAAGAACAAAGCAGTTTGTTTCTCCTTCAACTACAGCACGGC
ATAAGGGATATAGTTTTGCTGAATAAACCTTGTGCTGCAAGCACGCGTTCGTTTGCACTTCACTGCACTAAA
GAGAAACAAAAAGACAGTTTCTCTCATTGTGCTTGAGAAAGTCTGAAGGATTTACTTACTGTGTTTGAATAC
CCTTCTGTTCTACATGCAGGAGAACCTAGTCTTTATGTTATCAACCAGAAAAAAGTGTTTTGCTTTTCTGCC
ATCCAGATACGTGATACCGTAAGAGGTTTAAATGTTTTAAAATGCACTTGAGCTGCACAACCTCGACTATG
CTTTGCAGACAGAATCTCTGAAGGAGAAGGCATTTCGAGGTTCTCCCAGTCAAGTATAGGATGGCTATAGA
GATGTAGTTTTTGAAGCAAACACTGTTAGCACAACGTGCAACTTCTC
>NW004444509.1_9
ACTCACTGCCTGAAACAGAAGCAAAATAAAGTGTATTCACTCTAGTTGGTTAACTTAATTAAGTTGACGTTA
TCTGCTTTGAAAAACACAGTTCCTACATGGTGAAAACCTTTCTTACACGTAGTCTGAATGGGAAAACAATG
TCAAACTTCTTCCTTTCAAGTACTTGAGGCCCCTGGGTTTTCACATGTTTTCTAAATTTTAGGTTACCGGCAA
TGAATGTGCCTTTCAAATTCTAAGAAGAACAAGGCAGTTTGTTTCCCTTTCAACTACAGCATGGCAAAAGG
GATGTAGTTTTGCTGAATAAACCTTGTTCTGCAAGAACGGGCACGTTCACACTTCACTGCACGAAAGAGAA
ACCATAAGACAGTTTCTCTCATTGTGCTTAAGGAAGTCTGCAGGATTTACTTACTGTGTTCGAACACCTGTC
TGTGCTACATGTAGGAGAACCTTGCATTTTCTTTCTATAACAAGGAAAAGTGTCTCATTTTTCTGCCATCCA

AATCCGTGAATCCATACGGTGTTTAGATGCTTTTAAGATGCGCTTGAGCTGCGCAACCGTGAATGTGCTTTG
CAGACAGTATTTCTGAAGGAGAATGCAGTGCGAGCTTCTCCCGTTCAAGTATAGGATGGCTATCGAGATGT
AGTTTTGTGAAGTAAACACTGTGTTAGTGCAACGTGCATGTTCTT
>NW004444309.1_1
ATTCACCACATGAAAGAGAAAGCCAAATACAGCGTTCTCCTTCATGCTGCTTACCTGAATCTACTGAAGTTT
TCCGTTTTGTTAGAAGAAAGAGTCTGCCCACCTCAACTGCTTTCTCAAATGCTCTCTCAGTGGGAGAAGGCA
GTAGAGTGAGGGCCTGGCAAGAATTTGAATCCCTGGTTTTGAAAGGGAACTTTGCATAACAGGAATGAAGG
TATTGCTAGCTTACAACGCCAAACCGGCAGATAGTTTGCCTTCAGCTAGAGGACAGCAGAAGAGGTTCAGT
TTAGCTAACTAAACCTTGTGTGCTTAACACGAGCATGTAGCTTCTTCCCTGCCTGAAATAGAAAGCAAGAG
CTTGCTGCTCTCAGTCTGGGGATGAAGGGAGAACGATTCACTTTCTGTGTTTGAAAATCCCTCTTTGTTACA
TGCCTGCAAATGCTGCCTGTGGATTGTCTACCTGGATAAAGTGTATGTCTTTTCTACCTTTCAAGTAGTTGA
AGACGTTCAAGGCTTCCAGCATTCCGGGAGACATTCTGTGCTGCGCGGCTGAGAATACCGCTTTACAGACG
CTATCCCGAAACGAGAAGAGAAACCGCGTTTCTGCCATTCTGGAACACGAATGAGGCAAAAGCGTAGTTGT
GCTGAGTAAGAACTTTGTTAGCAGCAAGTGTAAATTCCCC
>NW004444309.1_2
CTTCACCACATGAAAGAGAAAGCCAAATACAGCGTTCTCCTTCATGCTGCTTAACTGAATCTTCTGAAGTTT
GGTGTTTTGTTAGAAGAAAGTGTCCGCCCATCACAAGATGCCTTCTCAATGGTTCTCTCAGTGGGAGAAGA
CTGGAGAGTGCCTGCCTTGCAAGTATTTCAACCCCCTGGTTTTCAAAGGGAACTTTGTCTAACAGACAGTGA
AGGTACTACTGGCTTTCAAAACAAAACAGGCAGATTGTTTGCCTTCAGCTAGAAGATGGCAGAAGAGGTTC
AGTTTTGCTGACTAAACCTTGTCTGCTTAACGCGAGCATGTAGCTTCTTCCCAGCCTGAAACAGAAAGCAA
GAGCTTGTTGCTCTCGGTCTGGGAATGAATTGTGAAGGATTCACTCTCAGTGTTGGAAAATCCCTGTATGTT
CCATGCCTAAGAAAGTTGCCTGTGCAGTGTCTACTTGGCTAAAGTGTATGACTTTTCTCCCTTTTGAGAAAC
TGAAGCCATTCAGGTTTTACAGCTTTCCGGGAAGCCTTCTGTGCTGCGTGGCTGCGAATACCGCTTTACAGA
AGGTATCCCTAAAGGAGAAGAGAAAGGGCGTTTCTCCCGTACTAGAACACAGCACGGCAGAGCCGCCGTT
TTGCTGAGTAAGAACTATGTTAGCAGCAAGTTAACTTCCCA
>NW004444309.1_3
ATTCGCCGCATGAGAGAGAAAACGAAACACAGCGTTCTCCTTCAAGCTGCTTAACTGAATCTACTGAAGTT
TACAGTTTTGTGAGAGGAAACAATCAGCCCACCACCAGCTGCTTTCTCCAAGGTTCTCTCAGTGGGAGAAG
ACTGGAGAGTGCCTGCCTTGCAAGTATTTCAATCCCCTGGTTTTCAAAGGGAACATTACATGACAGGAATG
ACGGTATTACTAGCTTACAAAGCCAAAGAGGCAGATTGTTTGCCTTCAGGTAGAGGACAGCAGAAGAGGTT
TAGTTTTGCTGACTAAACCTTGTGTGCTTAACACGAGCATGTAGCTTCTTCCCTGCCTAAAAGAGAAAGCAA
GAGCTTGCTGCTCTCAGTCTGGGGATGAAGGGAGAACGATTCACTTCCTGTGTTTGAAAATCCCTCTGTGTT
CCATGCCTAAGAAAGTTGCCTGTGCATTGTCTACCTGGATAAAGTGTATGACTTTTCTCCCTTTCAAGTAGT
TGAAGCCGTTCAGTGTTTCCAGCATTCCGGGAGACATTCTGTGCTGCGCGGCTGAGAATACTGCTTTAAAG
AACGTATCCCTAAAGGAGAAGAGAAACCGCGTTTCTCCCGTACTAGAACACAGCACTGCAGAGCCGCCGTT
TTGATGACTAAGAACTATGTTAACAGCAAGTGTAAGTTCCCA
>NW004444309.1_4
ATTCGCCGCATGAGAGAGAAAGCGAAACACAGCTTTCTCGTTCAAGCTGCTTAACTGAATCTACTGAAGTT
TTCAGTTTTGTGAGAGGAGACAATCTGCCCACCACCAGCTGCTTTCTCAAAGGTTCTCTCAGTGGTTTTCAA
AGGGAACTTTGCATAACAGACAGTGAAGGTACTACTGGATTTCAAAGCAAAACAGGCAGATTGTTTGCCTT
CAGCTAGAGGATGGCAGAAGAGGTTCAGTTTTGCTGACTACACCTTATGTGGTTAACGTGAGCATGTAGAT
TCTTCACGGGCAGAAAGAGAAAGTAAGAGCCTGTTGTTCTCAGTCTGGGGATGAAGTGAGAAGGATTCAGT
CCCAGTGTTGGAAAATCCCTCTATGTTCCATGCCTAAGAAAGTTGCCTGTGCAGTGTCTACCTGGATAAAAT
GTATGACTTTTCTCCCTCTCAAGTAGTGGAAGCCGTGCAGGGTTTACAGCATTCCGGGAGGCCTTCTGTGCT
GCGCGGCTGAGAATACCGCTTTACTGACGCTATCCCTAAAGGAGAAGAGAAACCGCGTTTCTCCCTTTCTG
GAACACGGATGAGGCAAAACTGTAGTTGTGCTGAGTAAAAAGTTTGTTACCAGCAAGTGTAAGTTCCCAT
>NW004444309.1_5
ATTCACCACTTAAAAGAGAAAGCAATATACAGAGTTCTCCTTCAAGCTGCTTAATGGAACATACTGAAGTT
TAATCATTTCTTAGAAGAAACAGTCCGCCCAGGAGAAGCTGCTCTCTCAAAGGTTGTCTCAGTGGGAGAAG
ACTGGAGAATGCCTGCCGTGCAAGTATTTCAATCCCCTGGTTTTCAAAGGGAACTTTGCATAACAGACAGT
GAAGGTACTACTGGCTTTCAAACCAAAACAGGCAGATTGTTTGCCTTCAGCTAGAGGATGGCAGAAGACAT
TCATTTGCTGACTACACCTTTTGTGGTTAACGTGAGCATGTAGAATCTTCACGGGCAGAAAGAGAAAGCAA
GAGCCTGTTGCTCTCAGTCTGGGGATGAAGTGAGAAGGATTCACTTACTGTGGTTGAAAATCCCTCTGTGTT
CTATGCCTAAGAAAGTTGCCTGTACAGTGTCTACCTGGATAAAGTGTATGACTTTTCTCCCTTTCAAGTACT
CGAAGCAATTCAGGCTTTACACCATTCCGGAAAGCATCCCATGCTGCGCGTCTGCGTATACCACTTTACAG
AATGTATCCCTAAAGGAGAAGAGAAACCGCGTTTCTCCCGTAGTAGAACACAGCACGGCCGAGCCGCCGTT
TTGTTGAGTAAGAACTATGTTAGCAACAAGTGTAAGTTCGCA

>NW004444309.1_6
ATTGGCTGCATGAGAGAGAAAGCGAAACACAGCATTCTCCTTAAAGCTGCTTTACTGAATCTACTGAAGTT
TTCAGTTTTGTTAGAGGAAACAGTCCGCCCACCACCAGCTGCTTTCTCAAAGGTTCTCTCCGTGGGAGAAGA
CTGGAGAGTGCCTGCCTTGCAAGTATTTGAATCCCCTGGTTTTCAAAGGGAACTTTGCATAACAGGGAATG
AGGGTATTTCTGTCTCTCAAAGCAAAACAGGCAAATTGTTTTCCTTCAGCTAGAGGATGGCAGAAGACATT
CATTTGCTGACTACACCTTTTGTGGTTAAAGTGAGCATGTAGAATCTTCACGGGCAGAAAGAGAAAGCAAG
AGCCTGTTGCTCTCAGTCTGGGGATGAAGTGAGAAAGATTCACTTCCAGTGTTGGAAAATCCCTCTATGTTC
CATGCCTAAGAAAGTTGCCTGTGCAGTGTCTACTTGGATAAAGTGTATGACTTTTCTCCCTTTCGAGTAGTT
GAAGCCCTTCAGGGTTTATAGCATTCCGGGAAGCCTTCTCTGCTGCGCGGCTGCGAATACCGCTTTACAGA
AGGTATCCCTAAAGGAAAAGAGAAACTGCGTTTCTCCCTTTCTGGAACACAAACGAGGCAAAACCGTAGTT
GTGCTGGGTAAAAACTTTGTCAGCAGCAAGTATAAGTTCCCA
>NW004444309.1_7
ATACACTGCATGAAAGAGAAAGCAAAGTACAGAGTTCTGTTTGAAGCTGCTTAACTGAATCTACTGAAGTT
TTCAGTCTTGTTAGAAGAAACCTTCCCCCCACCAGAAGCTGCTTTCTCAAAGTTTGTCTCAGTGGGAGAAGC
CTGGAGAGTGCCTGCCTTGCAAGCTTTCGAATCCCCTGGTTTTCAAAGGAAAGTTTGCATAACAGAGAATG
AAGGTACTACTGGCTTTCAGAGCAAAACAGGCAGATTGTTTGCCTTCAGCTAGAGGATAGCAGAAGAGATT
CAGTATTGTTGAGTAAATATTGTGTGCTTAACTGGAGCATGTAGCTTCTTCCCTGCCAGAAAGAGAAAGAA
AGAGCGTGCTGCTCTCAGTCTGGGGATGAAGTGAGAATGATTCACTTCCAATGTTTGAAAATCCCTCTGTGT
TTCATGCCTAAGAAAGTTCCCTGTGTAGTGTCTACCTGGATAAAGTGTATGACTTTTCTCCCTTTCAAGTAGT
TGAAGTCATTCAGGCTTTACAGCATTTCGGGAAGCATTCGGTGCTGCGCGGTGAGAATACCTCTTTACACAC
GGTATCTGTAAATGAGAAGAATAACCGAGTTTCTCCAGTTCTGGAACACGAATCAGGCAAAAGCGTAGTTG
TGCTGAGTAAAAACTTTGCTAGCAGCAAGTGTAAGTTCCCA
>NW004444309.1_8
ATTCACCACTTAAAAGAGAAAGGAAAATACAGCGTTCTCCTTTAAGCTGCTGAACTGAATCTACTGAAATT
TTCAGTTTTGTTAGAAGAAACAGTCAGCCCAGCAGAAGCTGCTTTCTCAAAGGTTCTCTCAATGGGAGAAG
CCTGGAGAGGGCCTGCCTTGCAAGTATTTCAATCCCTTGGTTTTCAAAGGGAAGTTTTCATAACAGGGAAT
GAAGGTACTTCTGGCTTTCAAAGCAAAACAAGCAGATTGTTTGCCTTCAGCTAGAGGATGGCAGAAGAGGT
TCAATATCGCTGAATAGAACTTGTGTGCTTAAGGCGAGCATGTAGCTTCTTCCCTGCCTGAAAGAGAAAGC
AAGAGCTTTTTGCTCCCTGTGTGGGGATGGAGGATTCACTTACTGTGTTTGAAAATCTCTCTGAGTTTACAT
GCCCAAGAAAGTTGATTGTGTATTGTCTACCTCGTTAAAATGTATGACTTTTCTTCCTTTCAAGTAGTTGAA
GCCGTTCAGGGTTTAGATCATTCCGGGAGGCATTCTTTGCTGCGCGGCTGAGAATACCTCTTTACACACGGT
ATCCGTAAAAGAGAAGAATAACCGCGTTTCTCCCGTTCTGGAACATGAATGAGGCAAAACCGTAGTTGTGC
TGAGTAAAAACTTTGTTAGTTGCAAGTGTGAGTTCCCA
>NW004444309.1_9
TTTCACCACATGAAATAGAAAGGAAAATACCGCGTTCTCCTTGAACCTGCTTAACTGAATCTTCCGAAGTTT
TCAGTTTTGTTAGAAGAAACATTCCGCACACCTGAAGCTGCTTTCTCAAAGGTTCTCTCATTGGGAAAAGAC
TGGAGAGGGCTTTCCTTGCAAGTATTTGAATACCCTGGTTTTGAAAGGGAACTTTGCATAACAGGGAATGA
AAGTACTTCTGGCTTTCAAAGCAAAACAGGCAAATTGTTTGCCTTCAGCTAGAGGATGGCACAAGAGGTTC
AGTTTGGCTGACTAATCCTTGTGTGCTTCAAGCGAGCATGTAGCCTCTTTCCTGGAAGAAGGAGAAAGCAA
AGCATTTTGCTGTCTGTCCTTGGGAAGAAGTGAGAAGGATTCACTAACTGTGTTTGAAAATCCCTCTGTGTT
CCATGCCTAAGAAAGTTGCCTGTGCATTCTCTACCTCCGTAAAGGGATCCACTTTACTCCTTTTCAATTAGG
TGAAGTCATTCAGCGTTTACAACTTTCCAGGAGGCATTCTGTGCTCTGCGCTGAGAAAGCCGTCTTACAAAC
GGTATCGCTGAAGTAGAAGAGAAACCGCATTGCCCCCGTTCTGCTATACGACCACGGCAGAGACGCAGTTT
TGCTGAGTAAAGACTATGTTAGCAGCAAGTGTAAGTTCCCA
>NW004444309.1_10
ATTCACCGCATGAAACAGAGAGGAAATTACAGCGTTCTCCTTCAAGCTCCTTAACTGAATCTACTGAAGTTT
CCAGTTTTGTTAGAAGAAAGACTCAGACCAGCTGGAGCTGCTTTGTCAAATGCTCTCTCAGTGGGAGAAGA
CTGGAGAGTGCCTGCCTTGCAAGTATTTGAATCCCCTGGTTTTCAAAGGGAATTTTGCATATCAGGGAATGA
ATTTACCTTTAGCATTCAAAGCAAAACAGGCAGATTTGCCTTCAGCCAGAGGATGGCAGAAGAGGTTCAGT
TTTGCTGACTAAACCTTGTGTGTTTAAGGCGAGCACGTACCCTCGTCCCTGCCAGAAAGAGGAAGCAAATC
CTGTCGCTGTCAGTCTGGGGGATGAAGTGAGAAAGATTCAGTTACTGTGTTTCAAAATCCGTCTGTGTTCCA
TGCCTAAGAAAGTTGTCTCTGCATTGTCTACCTGGGTAAATGGATCCACTTTTCTCCTTTTCAAGTGGGTGA
AGCCCTTCAGCATTTACAGCTTTCCCGGAGGCATTCTGTGATGCACGCCGGAGAATGCCGCCTTACCGATGT
AATCGCTGAAGGAGAAGAGAAAGGGCCTTTCTCCCGTTCTGGTATACCACCGCGGCACAGCCGCAGTTTTG
CTGAGTAAAGACTATGTTAGCAGGAAATGTAAGTTCCA
>NW004444309.1_11
ATTCACCGCACAAAACAGAAAAATACAGCGTTCTCCTTCAAGCTGCTTAACTGAATCTTCAGAAGTTTCCA
GTTTTGTTAGAAGAAACAGTCCGACCAGCACGAGGTGCTTTCTAAAACATTGTCTCAGTGGGAGAAGACTG

GAGAGTGCTTGCCTTGCAAGTATTTGAATCCCCTGGTTTTCAAAGGGAACTTCGCATATCAGGGAATGAAG
GTACATTGGGCATTCAAAGCCAAACAGGCAGCTTGTTTGCCTTCAGCGAGAGGATGACAGAAGAGGTTCAG
TTTTGCTAACTAAACCTTGTGTGCTTAACGCCAGCATGTAGCTTCTTCCCCGATAGAAGGAGAAAGCAAAC
AAGCTGCTGTCAGTCTTGGGGAAGAAGTGAGAAGGATTCACTTAGTGTGTTTGAAAATCCGTCTGTATTCC
ATGCCTAAGAAAGTTGCCTGTGCATTGTCTACCTGGATGAAGGGACCCACTTTTCTCCCTTTCAAGTAGGTG
AGGCCATTCAGCGTTTACAGCTTTCCAGGAGGCATTCTGTGCTGTGCGCCCAAGAATGCCGCCTTACAGAG
GGTATCCCTAAAGGAGAAGAGAAAGCGCGTTTCTCTGGTTCTGGTAAACAACGGCGTCAGCGCCGCAGTTT
TGCTGAGTAAAGAACATTTTAGCAGCAAGTGTAGGTTCCCAA
>NW004444309.1_12
ATTGACTGCATGAAAGAGAAAGCAAAATACAGCTTTTCTCTTTCAAGCTGCTTCACTTAATCCACTGAAAGT
TTTCAGTTTTGGTAGAAGAAACAGTCCGCACACAACCAGCTGCTTTCTCAACCTTCTCTCCTTGGGATAAGA
GGGAGAGTATCTGCTTTGCAAGTACTTGAATCCCCTGGTTTTCAAACTGAACTTTGCATAACAGGAAAAGC
AGGTACCTTTGGCATTCAAAGGGAAACAGGCAGATTGTTTGCCATCAGCTAGATGGTGGCGGAAGAGGTTC
AGTTTTGATCTCTGAACCTTGTGTGCTTAACGAGAGCATGTAGCCTCTTCCGTGCCAGAAAGACAAAGCAA
GAGCCTGTTGCTCTCAGTCTGTTGGTGAAGTGAGAAGGATTCACTCACTGTGTTTGAAAATCCCTCTGTGTT
ACATGCCTGATAAGAGTTGCCTGTGCATTGTCTACCTGGGTAAACGGTTTGACTTTCATCCCGTTAGAGTTG
GTGAAGCCCTGTGCGGTTTACAGCTCTCTGGGAAGCGTTTTGTGCTGCATGGCCAAGAATGCTGCCTTCCAG
ACGGTATCCAGAAAGAAGAAGAGAAAGCGCCTTTCTCCCGTTCTGGTATACGAGTGTTTCAGTGCGGCTCG
TTTGCTGAGTAAAGACCATGTTAGCAGCAAGTGTAAGTTCCCG
>NW004444309.1_13
ATTCATTACATGAAATAGAAAGCAAACTACATCTTTCTCCCTCAAGCTGCTTCACTTAATCCACTGATTTTT
CAGTTTCGGTAGAAGAAAAATCCGCACGCCACCAGCTGCTTTCTCAAAAGTGCTCTCAGTGGGATTTAGAC
TGGAGAGTGTCTGCTTTGCAAGAACTTGAATCCCCTGGTTTTCAAGGGGAACTTTGCATTACAGGGAAGAA
GATAACATTAGTATCTGAAAGATAACAGGGAGATTGATTGCATTCAGCTAGAGGAAGGCAGAAGCCGTTC
AGTTTTGCTGACTAAAGCTTGTGTGTTTCACATGAGCAGGTAACCTATTCTCTGCAAGAGAGAGAAACCCA
AAGACCGTTGCTCTCACTCTGGGGGTTGAAGTGAGAAAGATTGACTTACTGTGTTTGAAAATCTCTCTGTGT
TACATACCTAAGAATCTTGCATTTGCATTGTTTATCTGAGTAAAGACTTCGACTTATATCCCGTTGGAGTAC
GTGAAGCCCTGTGGGGTTTAAATCTTTCTGGGAGGCACTCTTTGCTGTGTGGCTGAGAATGCCTCCTTAAAG
ACGGTATCCCTGAAGGAGGAGAGAAAGTGCATTTCTCCCCTTCTGCTCTACGGGCGCCGGCAGAGCTGTAG
TTTTTCTAACTAAACTCTATGTTAGCAGTTCGTACATGTTCCCAG
>NW004444309.1_14
ATTCACTGCATGAAGGAGAAAGAAAACTACAATCTTCTCATTCAAGCTGGTTAACTTAACTGAATTTTTCAG
TTTTGGTAGAACAAACTGTCTTCACACCACTTGCTGCTCTCTCAAACATTCTCTCAATAGGATAAGACTAGC
AAGTGTCAGCCTTTCAAGTACTTTACTCCCCTGGTTTTCAAAGGGAACTTTGCATTACAGGGAATGAAGGTA
CCTTTCGCATTCTATGAGAAACAAGCAGATTGCTTCCCTTCCAGTGGAAGATGGTAGAAGAGGTTCCGTTTT
GCAAACGGAAGCTTGTATGCTTTACACGATCGTGTTTCCTATTCCCTGCAAGAAAGAGAAACCAAAACACA
GTTGCTCTCCGTCTGGGGTATGAAGTGGAAGGATTCACTTCCTGTGTTTGAAAATCCATCTGTGTCAAGTG
GCTAAGAGAGTTGCCTGTGCATTGTCTACCTGGGTAAAGAGTTTGAATTCTCTCCAGTTCGAGTAGGTGAAG
ACCTGTGGGGTTACAGCTTTCTGGGAGGCAATCTGTGCCGCCCAGCAGAGAATGCCACCTTACAGACACTA
TCACTGAAGGAGAAGAGAAAGCACTTCTCTCCCTTTCAGGTATGTGACAGAGGCAGCGCTGTAGTTTTGTT
AACCAAACACTACATTAGCAGCACTTGCAAGTTCCCA
>NW004444309.1_15
ATTCACTGCATGAAAGAGAAAGCCAATTACAGTGTTCTCCTTCAAGTTGGTTAACTTAATCAACTGATGTTT
TGAGTTTTTATAGAACAAAGAGTCCTCACACCACAAGCTGCTTCCCAAACATTCCCTCAATGCGATAATACT
GGTAAGCGACTGCCTTTCAAGTTCTTGAAACCCCTGGTTTTCAAAGGGAACTTTGTATTACAGGGGATGAA
GGTACCTTTCACATTTTAACAGGCAGATTGATTGCCTTCAGCTGGAGGACTGCGGAAGCTGTTCAGTTTTGC
TGACTAAAGCTTGTATGCTTAACAGGAGCATTATCTGTTCCCTGCAAGAAAGAGAAACCAAAAGACAGTTG
CTCTCAGTCTACGGGATGAAGTGAGAAAGATTCACTTACTGTGTTTGAAAATCCGTCTGTGTTACATGCCCA
AGAGAATTGCCGATGCCTTGTCTAAGTGGATAAAGAGTTTGACTTTTCTCCCCTTCGAGTATGTGAAGGCCT
GTTGGGTTTACAGCTTTCCAGGAGGCACTCTGTGCTGTGCCGCTGAGAATGCCGCCTTACAGTCGCTATCTC
TGAAGGAGAAGGGAAAACATCTTTCTCCTATTCTAGTATACGACAGCAGCAACGCTGTAGTTTTGCTAACT
AAACCCTATGGTAGCAGCAAGTGTGTGTTCCCAC
>NW004444309.1_16
GTTTACTGCATGAAAGAGAAAGCAACAGACAGTGGTGACACTCAAGCTGGTTAATTTAATCAACTGAAGGT
TTCGGTTTTGGTAGAACAAAAAGTCCTCACACCACCAGCTGCTTTCTCAAACATTATGTCAATGGGATAAGC
CTGGCGAGTGTCTCCCAAGTACTTGAATTCCCTGGTTTTGAAAGGGAAGATTGCATTACAGGGAATGATTA
CCTTTCACATTCTATGAAAAACAGGCTGTTTGTTCACCTTCAGCTAGAGGACCGCAGAAGCGGTTCAGTTTT

GCTGACTAAAGCTTGTGTGCCTCACATGAGGATGTATCATATTCCCTGCAAGAAAGCGAAACCAAAAGACA
GTTGCTGTCATTCTGGCGGATTAAGTGAGAAGGATTCACTTACTGTGTTTGAAAATCCATCTGTGTTACGTG
CCCATGACTGTTATCTTTGCATGGTCTGCCTGGGTGAAGAGTTTGACTTTTCTCCCCTTCGAGTCCGTGAAG
CCTTACGGGGTTTACACCTTCCTGGAGGCACTCCGTGCTGCGCAGCAGAGAATGCTGCCTTACAGACGGTA
TCCCTGAAAGAGGAGAGAAAGCGCATTTCTCCCATTCTGCTACAGGACAGGGGCGGCACTGTAGTTTTATT
AACTAAACCCTATGGTAGCAGCACGTGCATATTCCCAC
>NW004444309.1_17
GTTCACTGCATGAAAGAGAAAGCCAACTACAGTCTTCTCATTCAAGCTGGTTAACTTAATCAACTTAAGTTT
TCAAATTTGGTAGAACAAAGAGTCCTCACACCACCAGCTGCTTTCTCAAACATTCTCTCAATGGGATAAGA
TTGACGAGTGTCTGCTTTACAAGTACTTGAATCCCCTCTGTTTCAAAGGGAACTTTGCGTTACAGGGAATGA
AGGTACATTTTGCATTCTAAGAGAAACAGGCAGATTGTTTGCCTCCAGCTAGATGATGGCAGAAGCAGTTC
CGTTTCCCTGAGTAAATCTTGTCTTGTCAGCACGTGAATGTTTCCTATTCACTGCAGAAATGAGAAAACAAA
AGATAGTTGCCCTCAGTCCAGTGCATGAACTGAGAAGGATTCACTTACTGTCTTTGGAAATCCCTCTGTGTT
ACTTGCCCAGGACTGTTCCGTGTGCACTCTTTACCTGGGTAAAGACTTCCACTTTTCTCCATTTCGACTACAT
GAAGCTCTGCGGGTTTCCAGCTCTCTGGGAGACACTCTATGCTGTGCAGCCAAGAATGCAGCCTTACAGCC
GGTATCGCTGAGGGAGAAAACAAAGCGCATTTCTCCCATTCTGGCATACGACAGCAGTAGAGATGTAGTTT
TGTAAAGTAAACACTATGTTAACAGGATGTGCATGTTCCCA
>NW004444309.1_18
ATTTACCGTATGGAAGAGACAGCAAAATAGAGTGTTCTCATTTAATCTGATTAACTTAATCAAGCTAAGCT
ATCAGTTTTGATAAAACACAGTCATCACACTATCTACAAGCTTTCTCAAACATTCTGTCAGTGGAATAGTAC
TGGCGAGTCTCTGCCTTTCAAGTACCTGAATCCTGAGGTTTTCAAAGGGAAATTTTCATTACAGTTAATGAA
GTAACTTTCTCATTCTATGAAGAACAAGCAGATTATTTTCCCTTCAGCTATAGGATGGCGAAAGTGGTTCAG
TTTTGCTGACTAAACTTTATGTTGTAAGCACGTGCATGTTTCCTATTCACTGCAAGAAAGGGAAACCAAAAT
ACAGTTGCTCTCATTCTCGAGGACGAATTGAGAAGGATTCACTTACTGTGTTTGAAAATCCCTTTGTGTTAC
ATGTTCAGGAATGTTGCCTGTACATTCTCTACCTGGGTAAATCGTTAGACCTTTCTTCCCTTCGACTACGTGA
AGCCCTGCAGGGTTTATAGCTTTCCACATTGTGCTGTGTGGTTGAGAATGCAACCTTACAGCCTGTATCCCT
GAGAGAGAAAAAAAAAACGAGTTTCTCCCTTTTTGGCATAGGATAGTAGTAGGAATGTATTTTTGCTAAGT
AAACACTATGTTAGCAGGATGTGTATG
>NW004444309.1_19
GTTCAGTGCATGAAAGAGAAAGCAAGATGCAGTGTTCTCACTCAAATTGGTTAACTTAAACAATTTAAGTT
ATCAGTTTTGATAAAATACAGTCCTCACACCATCTATAAGCTTTCTCAAACATTCTGTCAATGGGATAAGAC
TGGTGAGTCTCTGCCTGCAAGTACCTGAATCCCTTGGTTTTCTAAGGGAACATTGCTTTACAGGGAATGAAG
GTATTTTTCGCAGTATACAAAGAAGATTGTTTCATCTTCAGCTAAAGCATGGCAGAAGGGGGTCAGTTTTTC
TGACTAAACCTTGTGTTGTAACCACGTGCATGTGTTCTATTCACTGGAAGAACGAGAAACCAAAAGACAGT
TGCTCTCAGTCTGGTGAATGAAGTGAGAAGGATTCACTTACTGTGTTTGAAAGTGCCTGTGTTACATGCCCA
AGAATGTTGCCTATGCATTCTCCTCCTGGGTAAAGAGTTTGACTTTTCTCCTCTTGGAGTACTTGAAGCCCT
GCAGGGTTTACAGCTTTCCGGGAGGCTCTCTGTGCTGTCTGGCAGAGAATGAGGCCTTACAATCGGTATCC
CTGAGGGAGAAAACAAATCGCGGTTTCCCCCGTTGTGGCATATGACAGTATTACAGATGTAGTCTCGGTAA
ATAAACACTATGTTAGCAGGAAGTTCATGCTTCCA

Supplementary Sequence File 2. TMAsat monomeric sequences (in fasta) manually isolated from the *Dugong dugon* assembled reference genome (accession GCA_015147995.1).

>BMBL01009774.1_1
ATTCACCACATGAAAGAGAAAGCAAAATACAGCGTTCTCCTCAAGCTGCTTAACTGAATCTACTCAAGTTT
TCAGTTTTGTTAGAAGAAAGTGTCTGCCCAGCACAAGCTGCTTTCTCTAATGTTCCCTGCGTGGGAGAAGGC
TGGAGAGTGCAGGCCTAGCAAGTATTTGAATCCCCTGCTTTTGAAAGGGAATTTTTCCTACCAGGAATGAA
GGTACTAATGGCTTTCCAAGCCAAACAGGTAGATTGTCTGCCTTCAGCTAGAGGACAGCAGAAGAGGTTCA
GTTTTGCTGACTAAAGCTTGTTTGCTTAACGGGAGCATGTAGCTTCTTCCCTGCCTGAAAGAGAAAGCAAG
AGCTTGTTGCTCTCAGTCTGGGGATGAACGGAGCACGATTCACTTTCTGTTTTTCAAAGTCCCTCTGTGTTA
CATGCCTAAGAAAGTTGCCTGTGCATTGTCTACCTGGATAAAGCGTATGACTTTTCTCCCTTTCAACTACTT
GAAGCCGTTCAGGCTTTCCAGCATTCCGGGAGGCATTCTGTGCTGCGTAGCTGAGAATAGGGCTTTACAGA
CGCTATCGCTAAAGGAGAAGAGAAACCGCATCTCTCCCATTCTGGAACGCGAAGGAGGCAAAACCGGAGT
TCTCCTGAGTAAAAACCTTGTTAGCAGTAAGTTTAAGTTCCCT
>BMBL01009774.1_2
ATTCACCATATGAAAGAGAAAGCAAAAGACAGGCTTCCCTTCAAGCTGCTTAACTGAATCTACTTAAGTTT
TCAGTTTTATTAGAAGAAAGAGTCCGCCCAGCACAAGCTGCTTTCTCCAATGTTCTCTGAGTGGGAGAAGG

CTGGAGAGTGCAGGCCTAGCAAGTATTTGAATCCCCTGGTTTTCAAAGGGAATTTTGCATACCAGGGCATG
AAGGTACTATTGGCTTTCAAAGCCAAACAGGTAGATTGTCTGCCTTCAGCTAGAGGAAGGCAGTAGAGGTT
CAGGTTTGCTGACTAAAGCTTGTGTGCTTAACAGGAGCATGTAGCTTCTTCCCTGCCTGAAAGAGAAAGCA
AGAGCTTGATGTTCTCAGTCTCGGGACGAAGGGAGAATTATTCACTTTCTGTGTTTGAAAATCCCTGTGTGT
TACATGACTAAGAAAGCTGCCTGGGCATTTTCTACCTGGATAAAGCGTATGACTTTTCTCAATTTCAAGCAG
TTGAAGCCGTTCAGGTTTTCCAGCATTCCGGGAGTCATTCTGGTCTGCGCGGTTGAGAATACCGCTTCACAG
GCGGTATCCGTAAAGGAGAAGAGAAACAGCGTTTCTCCCATTCTGGAACGCGAGGGAGTCAAAACTGTAG
TTGTGCTGAGTAAAACCTTTGTTAGGAGCAAGTGGAAGTTCCCC
>BMBL01009774.1_3
ATTCACCGCATGAAACAGAAAGCGCACTACAGCGTTCTTCTTCAAGCTGCTTAAGTGAATCGACTGAAGTT
TTCAGTTTTGTTAAAAGGAACCGTCCGAAGAGCACCAGCTTCTTCCTCAAACGTTGCCTCACTGGGAGAAG
TCTGGAGAGTGCCTGCCTTGCAAGTATTTGAATCCGGTGGTTTTCCAAGGGAACTTTGCATATTAAGGAATG
AATGGACTACTGGCTCTCAGAGCCAAACAGGCAGATTGCCTGCCTTCAGCTAGAGGAAGGCAGAAGCGGT
TCAGTTTTGCTGACTAAACCGTGTGTGCTTAACACGAGCATGTAGCTTCTTCCCTGCCTGAAAGAGGAAGCA
AGAGCTTTTTGCTCTCAGTCTGGGGAGGAAGGGAGAACAATTCCCTTTCTGTGTTTGAAAATCCCTCTGTGT
TACATGCCTAAGAAAGTTGCCTGTGCATTGTCTACCTGGATGAAGTGTATGACTTTTCTCCCTTTCAAGTAG
TTGAAGCTGTTCAGGGTTTCCAGCATTCCGGGAGGCATTCTGTGCTGCGCGGCTGAGAATACCGCTTTACAG
ACGGTATCTCTAAAGGAGAAGAGAAACCGCGTTTCTCCCATTCTGGAACCCGAGGGAGGCAAAACCATAG
CTGTGCTGAGTAAAAGCTTTGTTAGGAGCAAGTGGAAGTTCCCC
>BMBL01009774.1_4
ATTCACCACATGAAAGAGAAAGCAGAAGACAGCACTGTCCTTCAACCTGGTTAACTAAATCTACTGAAGTT
TTCAGGTTTGTTAGAAGGAAGTGTCCGCCCAGAACAAGCTGCATTCTTCAAATGTTCTCTCAGTGGGAGAA
GACTGGAGAGTGCGGGGCTAGCAATTATTTGAATCCCCTTTTTTTCCAAGGGAACTTTGCATACCAGGGAA
TGAAGGTACTACTGGCTTTCAGATCCAAACAGCCAGATTGTTGCCTTCTGCTAGAGGACAGCAGAAGAGGT
TCAGTTTTGCTGACTAAACCTTGTGTGGTTAATAGGATCCTGTATCGTCTTCCCTGCATGAAAGTGAAAGCA
AGATCTTGTTGCTCTCAGTCTGGGTAGGGAGAACAGTTCACTTTCTGGGTTTGAAAAACCCTCTGTGTTACA
TGCCTAAGAAACTTGCCTGTGCATTGTCTACCTGGATAAAGTGTATGACTTTTCTCCCTTTCAAGAGGTTGA
AGCCATTCAGGGTTTCCAGCATTCCGGGAGGAATTCTTTGCTGCGCGGCTGAAAATACCGCCATACAGGCA
GTATCGCTAAAGGAGAAGAGAAACCCCGTTTCTCCCATTCTGGAACGCGAGGGAGTCAAAACCGTTGTTGT
GCTGAGTAATAACTTTGTTAGCAGGAAGTGGAAGTTCCCC
>BMBL01009774.1_5
ATTTAGCACATGAAAGAGAAAGCAAAATACAGCGTTCTCCTTGAAGCTGCTTAACTGAATTTACTGATGTTT
TCAGTTTTGTTAGAAGGAAGAGTCCATCCAGCACAAGCTGCTTTCTCCAATGTTCTCTGAGAGGGAGAAGG
ATGGAGAGTGCGGGCCTAGCAAGTGTTTGAATACCCTGGTTTACAAAGGGAACTTTGCATATCAGGGAATA
AATGTACTGCTGGCTTTCAGAGCTGAACAGGCAGATTGTCTGTCTTCAGCTAGAGGACAGCAGAAGAGACT
CAGTTTTGCTGACTAAATCTTGTGTGCTTAACACTAGCCTGTAGCTTCTTCGCTGCCTGAAAGTGAAAGCAA
GAGCTTGTTGCTCTCAGTCTGGGGACGAAGGGAGAAGGATTCACTTTCTGTGTTTGAAAATCCCTCTGTGTT
ACATGCCTAAGAAAGATGCATGTGCTTTGTCTACCTGGATAAAGTGTATGACTTTTCTCCCTTTCAAGAGGT
TGAAGCCGTTCAGGTTTTCCAGCATTCTGGGAGGCATTCTGTGCTGTGTAGATGAGAATAGGGCTTTACAAG
CGGTATCGCTAAAGGGGAAGAGAAACCGCGTTTCTCCCATTCTGGAACGCGAGGGAGTCGAAACTGTATTT
GTGCTGAGTAAAAACTTTGTTAGGAGCAAGTGGAAGTTTCCC
>BMBL01009774.1_6
ATTCACCACATGAAAGAGAAAGCAAGATACAGCGTTCTCCTTCAAGCTGCTTAACTGAATCTACTGACGTT
TTCGGTTTTCTTAGAAGAAAGGGTCTGCCCAGCACAAGCTGCTTTGTCAAACGTTCTCTGAGTGGGAGAAG
ACTGGAGAGTGAGGGCCTAGCAAGTGTTTGAATCCCCAGGTTTACAAAGGGAACTTTGCATAGCAGGGAAT
GAATGTACTACTGGTTTCAGAGCCAAACAGGCAGATTGTCTGCCTTCAGCTGGAGGAAGGCTGAATACGTT
CAGTTTTGTTGAGTAAACCTCTTGTTCTTAACATGCGTCTGTAGCTTCTTCCCTGTTTGACAGAGAAAGCAA
GACCTTGTTGCTATGACTCTGGGGATGAAGGGAGAAGGATTCACTTTCTGTGTTTGAAAATCCCTCTGTGTT
ACATGCCTAAGAAAGTTGCCTGTGCATTGTCTACCTGGATAAAGCGTATGACTTTTCTCCCTTTCAAGTAGT
TGAAGCCGTTCAGGGTTTCCAGCATTCCGGGAGGCATTCTGTGCTGCGCGGCTGAGAATACCCTTTACAGA
CGGTATCGCTAAAAGAGAAGAGAAACCGCGTTTCTCCCTTTCTGGAACGCGAAAGAGGCTTAACCGCAGTT
GTGCGGAGTAAAGACTGTTAGCAGCAAGTGCCAGTTGCCC
>BMBL01009774.1_7
ATTCACCACATGGAAGAGAAAGAAAACTACAGCGTTCTCCTTCAAGCTGCTTAACTGAATCTACTGCAGTT
TTCAGTTTCTTTGGAAGAAAGTGTCGGCCCAACACAAGCTGCTTTCTCCAATGTTCTCTCAGTGGGATTAGG
CTGCAGAGTCCGGGCCTAGCAAGGATTTAAATCCCTTGGTTTTCAAAAGGAACTTTGCATACCAGGGAATG
AGGGGACTACTGGCTTTCAACGCCAAATTGGCAAATTGTCTGCCTTCAGTTAGAGGACGGCAGAAGAGGTT

CAGGTTTGCTGACAAAACCGTGTCTGCTTAACAGGAGCATGTAGCTTCTTCCCTGCCTGAAAGAGAAAGCG
AGAGCTTGCTGCTCTCAGTCTGTGGACAAAGGGAGAATGATTCACTTTCTGTATTTGAAAATCCCTCGGTGT
TACATGCCTAGGAAAGTTGCCTGTGCATGGTCTACCTGGATAAAGGGTATGACGTTTCTCCCTTTCAAGTAG
TTGAAGCCGTTCAGTGTTCCTACCATTCCTTGAGGCTTTCTGGGCTGCGCGGCTGAGAATACCGCTTTACAG
ACGGTATCGCTAAAGGAGAAGAGAAACCACGTTTCTCCCACCTGGAACTTGAAGGAGGCAAAATCGTAGT
TGTGCTGAGTAGAAACTTTGTTAGCAGCAAGTGTAAGTTCCCC
>BMBL01002074.1_1
ATTCACCACATGAAAGAGAAAGCAAAACACAGCGTCTTCCTTCAAGCTGCTTAAGGGAATGAACTGAAGTT
TTCAGTTTGGATAGAAGAAAGTTTCTGCCCAGCACAAGCTGCTTTCTCAAATGTTCTCTGAGTGGGAGAAG
GCTGGAGAGTGCGGGCCTAGCAAGTATTTGAATCCCCTGGTTTTCAAAGGGAACTTTGTATACCAGGGAAT
GAATGGAGTACTGGCTTTCAAAGCCAAACAGGCAGATCGTCTGCCTTCAGCTAGAGCACGGCAGAAGAGG
TTCGTTTTTGTTGACTAAACCGTCTGTGCTGAACACGAGCATGTAACTTCTCCCCTGCCTGAAAGAGAAAGC
AAGAGCTTGTTGCTCTCAGGATGGGGATCTATGGAGAATGATTCAATTTCTCTGTTTGAAAATCCCTGAGTG
TTACATGCCTAAGAAAGTTGCCTGTGCATTGTCTACCTGGGTAATGTGTATGACGTTTCTCCCTTTCACGTA
GTTGAAGCCGTTCAGGGTTTCCAGCATTCCATAAGGCATTCTGTGCTGCGTGGCCCAGAATTCGGCTTTACA
GACGGTAACGCTAAAGGAGAAGAGAAAACGCGTTTCTCCCATTCTGGAACGCGAAGGGGGAAAACCGTAG
TTGTGCTGAGTAAAAACTTTGTTAGCAGCAAGTGCAAGTTGCCC
>BMBL01002074.1_2
ATTCACTACATGAAAGGGAAAGCAAAATACGGCGTTCTCCTTCAAGCTTCTTAACAGAATCTACTGAAGTT
TTCAGTTTTGTTGGAAGAAAGAGTCCGCCCAACACAAGCTGCTTTCTCAAGAGTTCTCTGAGTGGGAGAAG
GCTGGAGAGTGCTGGCCTAGCAGGTATTTGAATCCTCTGGTTTTCAAAGCGAACTTTGCATCCAGGGAATTC
AGGTACTACTGGCTTTCAACGCCAAACTGGCAGAGTTTCTGCTTTGAGCTAGAGGACGGCAGAAGAGGTTC
AGTTTTGTTGACTAAATCTTGTGTGCTTAACACGAGCATGTAGCTTCTTCCCTGCCTGATAGAGAATGCAAC
TGCTTGTTGCCTTCAGTCTGAGGATGAAGGCTGAAAGATTCACTTTCTGTGTGTGACAATCCCTCTGTGTTA
CATGCCTAAGAAAGTTGCCTGTGCATTGTCTACCTGGATAAAGTGTATGACGTTTCTCCCTTTCAAGTAGTT
GAAGCCGTTCAGGGTTTCCAGCATTCCGGGAGGCATTCTGTGCTGCGCGGCTGAGACTACCCGTATAACCA
CGAAAGAACTGGTCTGCCAATCTGCTTCCTTTAAGATTACAACCAAGAAAACTCTATGGAGCAC
>BMBL01012666.1_1
ATTCACCGCGTGAAAGAGAAAGAAAAATACAGCATTCTCCTTCAAGCTGCTTAACAGAAGCTACTGAAGTT
TTCAGGTATGTTAGAAGAAACAGTCCTCCCACAACCAGCTGCTTTCTCAAACTTTCTCTCAGTGGGCTAAGA
CTGTAGAGTGCTTGTCTTGCAAGTATTTGAATTCCCTGGTTTTCAAAGGGAAATTTGCATCCCAGGGAATGA
AGGTGGTTTTGGCATTCAAAGCAAAGCAGGCAGATTGTTTGCCTTCAGCTAGGGGACGGCCGAATTGTTTC
AGTTTGGCGGAATAAACCTTGTGTGCTTAACGCCAGAAAGTAGCTTCTTCCATGCCTGAAGGAGAAAGCAA
GAGCCAGTTGCTGTGAGTCTGGGGAAGAAGTAAGGAGGAGTCACTTACTGTGTTTGAAAATCCCTCCGTGT
TACATGCCTAAGAAAGTTGCCTGTGCATTGTCTACCTGGATAAAGCGTATGACTTTTCTCCCTTTCAAGTAG
TTGAAGCCGTTCAGTCTTTCCAGCATTCCTGGAGGCATCCTGTGCTGCGAAGCTGAGAATAGGGCTTTACAG
ACGGTACCGCTAAAGGAGAAGGAAACCGCGTTTATCCCATTCTGGAACGCGAAGGAGGCAAAACCGTAGT
TGTGCTGAGTAGAAATTTTGTTAGCAGCAATTCTAAGTTCCCC
>BMBL01012666.1_2
ATTCACCACATGAAAGACATGGGAAAATACAGCGTTCTCCTTCAAGCTGCTTAACTGAATCTACTTAAGTTT
TCAGTTTTGTTAGAGGAAAGAGTCCGCCCAGCAAAACTGCTTTCTCCAATGTTCTCTGAGTTGGACAAGGCT
GGACGGTGCAGGCCCAGCAAGTATTTGAATCCCCTGGGTTTCTAAGGGAAGTTTGCATACCAGGGAATGAA
GGTACTGTTGGCTTTCAAAGCCAGACAGGTAGATTGTGTGCCTTCAGCTAGAGGACAATAGAAGAGGTTCA
GATTTGCTGACTAAAGCTTGTGTGCGTAACACGAGCATGTAGCTTCTTCCCTGCCTGAAAGAGAAAGCAAG
AGCTTGTTGCTCTCAGTCTGTGGATGAGAGGAGCACGATTCACTTTCTGTGCTTGAAAATCCCTCTGTTTTA
CATGTCTAAGAAAGTTCCTGTGCATTGTGTACTTTGATAAAGTGTACGACGTTTCTCCCTTTCAAGCAGTTG
AAGACGTTCAGGGTTTCCAGCATTCCGGGAGGCATTCTGTGCTGCGCTGCTGAGAATACCGCTTTACAGAC
GGTATCGCTAAAGGAGAAGAGAAACGGCGTTTCTCCCATTCTGCAAGGCTAAGGAGGCAAAACCGTACTTC
TCCTGAGTAAAACATTTGTTAGCAGTAAGTTTTAGTTTCCT
>BMBL01099827.1_1
ATTCACTGCATGAAAGAGAAAGCAAAATACAGTGTTATCATTCAACCTGCTTAACTTAATCCACAGAAGTT
TTCAGTTTTGGTAGAACAAACAGTCCTCACACCACCAGCTGCTTTCTCAAACGTTCTATCAGTGGGATAAGA
CTGTCGAGTGTCTGCCTTGCAAGTACTTGAATCCTCTGGTTGTCAAAGGGAACTTTGCATTACAGGGACCGA
AGGTACTTTTGTCATTCAAAGTAAAACAGACAGATTGTTTGCCTTCAGCTAGAGGACAGCAGAAGCAGTTC
ACTTTTCCTGATGAAAGTTTGTGTGCTCAACAGGAGCATGTACATTATTCCTTGCAAGAAAGAGAAAGCAA
AAGACAGTCGCTCTCAGTCTGGGGGATGAAATGAGAAGGATTTACCTACTGTGTTTGAAACTCCCTGTGTG
TTACAAGCCCAAGAAAGTTGCCTGTGCATTGTCTACCAGAGTAAAGAGTTTGAGTTTTCTCCCAGTCGAGTT
CGTAAAGCTCTGCGGGATTTACAGCTTTCTGGGGGGCACTCTGTGATGCATGGAGAGAATGCCGCCTTACA

GACGGAATCCCTGAAGAAGAAAGAAAGTGCGTTTCTCTCATTCTGGTATACGACAGCAGCAGCGCGGTAGT
TTTCCTAAGTAAACACTATGTTAGCAATACTTGCATGTTCCGA
>BMBL01099827.1_2
ATTCCCAGTATGAAAGGGAAGGCAAAATACAGTGTTCACATTCAAGCTGGTTAAGTTAAGCAACTTAAGTT
TTCAGTATTGGTAGAACAAACGGTCCTCACAGTACCAGCTCATTTCTCAAACATTCTCTCAGTGGAATAAAA
CTGGCAAGTGTCTGCCTTTCAAGTACTTGAATCCTCTGCTTTTCAAAGGAACTTTTCATTACAGGGACTAAA
GGTAGCTTTTGCATTCTAAGCAAAACAGGCAGACTGTTTGCCTTCAGCTAGAGGATGTCAGCAGGGGTTCA
GTTTTGCTGACTAATCCTTGTGTGCTTAACACAAGCATTTCTTATTCCTTGCAAGAAAGAGAAAGCAGAAGA
CAGATGCTCTCAATCTGGGGGATAAAGTGAGAAGAAGTTACTCACTGTGTTTGAAAATCCCTCTCTGTTACT
GGCCCAGGACAGCTGCCTGTGCATTGTATACCTGGATAAGAGTTTGAATTTTCTCCCATTGGAGTTCGTGAA
GCCCTGCGGGGTTTACAGCTTTTCAGGAGGCACTCTGTGCTGCGAGGCCGAGAATTCCGCCTTACAGAGGG
TATCCCTAAAGGAGAAGAGAGCGCTTTTCTCCCGTCCTGGTATACGACAGTGGTAGCGCTGTAGTTTTGCTG
AGTAAAGATTATGTTAGCAGCAAGTGCAAGTTCCCA
>BMBL01099827.1_3
ATTCAATGCACGAAAGAGAAAGCAAACTACAGTGTTCTCCTTCAAGCTGGTTAACTTAACCCACTGAAGCT
TTCAGTTTTGGTAGAATACACAGTCATAACACAACCTCCTGCTTTCACAAAATTTCTCTCAGTGGGATAAGA
CTGTCGAGCGTCTTCCTTGCAAGTATTTGAATCCACTGCGTTTCAAAGGGAACATGCAGTACAGGGGATGG
AGGTACCTTTGGCATTCTAAGCAAAACAGGTGGATTGTTTTCCTTCAGCTAGAAGACAGCAGAAGCAGTTC
AGTTTTGCTGAGAAAACCTTGTGTGCTTAACACGAGCAGGTATCCTATTCCCTGCAAGATAGAGAAAGCAA
AAGACAGTTGGTCTCAGTCTGGGTGATGAAGTGAGGAGGACTCAATTACTGTGTTTGAATCCCTTGGTGTG
AAATGCCTAAGAAAGTTGCCTGAGCATTATCTACCTGGGTCAAGAGTTTGACTTTTCTCCCGTTTGTGGGGT
TTACAGCTTTCCAGGAGGCCTTTTGTGCTGCGCAGACGAGAATCTACCCTTACAGACGGTATCACTAAAGG
AGAAGAGAAATTGGGTTTCTCCCATTCTGGTATATGAAAGCGGCAGCATTGTAGTTTTGCTGAGTGAAGAC
TATGTTAGCAGCAAGTGTAAGTTCTCA
>BMBL01099827.1_4
ATTCACTTCATGAAAGAGAAAGCAAAATACAGCATTCTCCTTCAAGCTGCTTCACTTCATCCACTGAAGTTT
TCAGTTTTGGTAGAAGAAACAGTCTGCACACCACCAGCTGCTTTCTCAAAGTTTCTCTCAGGGGGATGAGA
CTGGAGACAGCCTGCCTTGCAAGTGTTTGAATCTCCTGGTTTTCAAACGGAACTTTGCATAACAGGAAATG
AAGGTAATTTTAGCACTCAAAGCCAAATAGGCAGATTGTTTGCCTTCAGCTAGAGGATGGCAGAAGAGGTT
CAGTTTTGCTGACTAAACAGTGTGCTTAACATGACCATGTAGTTTCTGCCATGCCAGAAAGAGGAAGCAAA
AGCCAGTTGCTCTCAGTGTGGGGGATGGAGTGAGAAAGATTCACTTACTGTGTTTGAAATCCCTCTGTGTTA
CATACCTAATAAAGTTGCCGGGGCATTGTCTACCTGGGTAAAGGGTTTGACTTTTCTCCTGTTTGAGTAGGT
GAGCCCTTACAGGGTTTACAGCTTTCTGGGAACCATTCTGTGCTGGGCAGCCGAGAATGCCGCCTTACAGA
CGGTATCCGAAAAGGAGAAGAGATACCGCGTTTCTCCTGTTCTGTTGTACACCAGTAGCTCTGTAGTTTTGC
TGAGTAAAGACTACGTTAGCAGCAAGTGCAAGTTCCCA
>BMBL01099827.1_7
ATTCATTGCCTGAAAGAGAAAGCAAAATACAGTGTTCTCATTCAAGCTGGTTAACTTAATCAACTGACATTT
TCAGTTTTGGTAGAAGAAACTGTCCTTACAGCACCAGTGTGCTTTCTCAAACATTCTCTCAATGGGATAAGA
GTGGCAAGTGTCTGCCTTTCAATTACTTTAATTCCCTGGTTTTCAAAGGGAGCTTTGCATTACAACGAATGA
AGGTAACTTTCGCATTCTAGGAGAACTGATTGTTTAACCTTCAGATATTTGACAGCAGAGGCGGTTCTGTTT
TTCTGACTAAACCTTGTGTTGTAACCACATGCATGTTTCCTATTCACTGCAACAAAGAGAAACTAAACAACA
GTTGCTGTCAGTCTGGTTGATGAACTGAGAAAGATTCACTTAATATGTTCGATAGTCCCTTTGTCTTACATG
TCCAAGAATGTTGCCTGTGAATTCTCTTCCAGGGTAAACAAAGCATTTCACTTTTCTCTCCTTAGAGTACAC
GAAGCCTTACGGAGTTTGCAGCTTTCCCCGGGGAACTCTGTGCTGGGCGCTCAAGAATGTGGCCTTAGAGA
CCTTATTCCTTAAGGAGAAAACACAAAGCCCATTTCTCCCGTTCTGATACATGATCGCAATAGAGACATAG
TTTTGCTAAGGAAACACTACAGTAGCAGAGCCTGCATGTTTCCT
>BMBL01099827.1_8
TCACTGCATAAAAAAGAAAAATCAGAATTCAGTGTGCTCATTCAAGCTGGTTAACTTAATCAACTTATTAGT
TTTGAGAAACACAGTTCTCCCACCACCTGCAAGATTTCTCAAACATTCTCTTGATGGGATATGACTGGCGAG
TCTCTCGTTTTCCAGTACTTGATTCCCCTGCTTTTCAGAGGGAACTTTGCATTACAGGGAATGAAGTTACCTT
TTGCATTCTCAGAAGAACAAGCAGATTGTTTCACCTTCAGGTAAGGGATAGCAGAGGCGGTTCAGTTTTAC
TGACTAAACCTTGTGTTTTATCTACATGCCTGTTTCTTATTAACTGCAACAAAGAGAAACCAAACGACAGTT
GCTCTCATTCTGGTGGATGAAGTGAGAAGGATTTACTTAATGTGCTTGAAAATCCCTCTGTGTTACATGCCT
AAGAATGTCGCCTATGCATTGTCTGCCTGGGTAAAGCATTTCACTTTTCTACCCTTGAAGTACATGAAGCCC
TGCGGGGTTTACAGCTTTCCGGAAGGCACTCTGTTGCGTGGCTGAAAATGTGGTCTTATAGACAGTATGTCT
GAATGAGAAAACAAAATGTGTTTCTCCTGTTCTGGTATACGACTTCAGCAGAGATGTAGTTTTGCTAAGGA
AACACCACGTTAGCAGGACCTGCGTGTTCCAA

>BMBL01099827.1_9
TTTCACTTCATGAGAGAGAAAGCAGAGTACAGTGTGTTCATTCAAGCTGGTTAACTTAATCAACTTATCAGT
TTTGAGAAAACACAGTCCTCCCACCATCTGCAAGCTCTCTCATTCTCTCAGTGGAATAAGACTGGCTTGTGT
CTGGCTTTCCAGTATTGAACTCCCTGCTTTTCAAAGGGAAGTTTGCATTACAGGGAAAGAAGGTAGCTTTCA
CATTCTAGGAAGAACAAGCAGACTGTTTGGCCTTCAGCTAAAGACTGCAGAGGGGGTTCAGTTTTTTGCAT
ATTTCCTATTCACTGCCGCAAAGAGAACCAAATGACACTTGCTCTCAGTCTGGTGGATTAAGTGAGAAGGA
TTCATTTACTGTGTCTGAAAATCTCTCTGTATCACATGCCAAGGAATGTTGCCTATGTATGCTCTACCAGGG
TAAAGAGATTGACTTTTCTCCCCTTCGAGTACGTGAAGCCCTGCAGGATTTACAGTTTTCAGGGAGGCACTC
TGTGCTGCCCGTTCAGAAAGCGGCCTGACAGAAGGTATCTCTGAATTAGAAAAAAAGTGCCTTTCTCCTGT
TCTTATATACGACAGTAGTAGAGATGTAGTTTTAGTATGTAAAGACTATGTTAGCAGAACTTGCATGTTCCC
A
>BMBL01099827.1_10
TTTACAGCATGAAAGAGAAACCAAAAATATATTGTTCTCATTCAAGTTTGTTAACTTAATCAATTGAATAAG
CTATCAGTTTTGATAAAACACTGTTCCCACACAATCGAGAAAATTTCTCATACATTCTCTGAATTGGAAATG
AATGTCCAGCTTCTCTCTTTCAAGAACTTGTATCCCCCGCATTTTCAGATGTTTTCTAAAGGAGTTTTGAGTT
ATAGGGAAATGTGTGTGCATTTCACATTCTAGGAAGAACAATGCAGACTGTTTCCTGTTCAACTAAAAGAT
GGCAAAACCGATATAGTTTTGCTGACTGAATCATGTGTGGTAACCACGTGTGTGTTTCCAATTCACTACAAG
AAAGAGAAACCAAAATTCAGTTTTCTCATTCAGATTGGTTAATTGAATCTAGTTGATTAAGTTGTCAGTTTT
GATAAAACACACTTCCCACAGTATCCACAACCTTCCTCATACGTTCTCTGAATGGGAAATGAATGTCATGTT
TCTCCCTTTCCAGTACTTGAATCCTCCAGGTTTTCAGACTTTTCTTATGGGAACTTTGCATTACTGGGAATGG
ATTTGCCTTTCACAATCTAGGAAGAACAAGGCAGATTGTTTCCCATTCATCTAAAAGATGGCAAAAACATT
GTACTTTGCTGACTGAACCCTGTGTGGTAAGCACTTGTATGTTTCCA
>BMBL01099827.1_14
ATTCACTGCATGAAAGCGAAACCAAAACTGTTTTCTCATTCTAGTTGATTAACTTAATCTGGTTGATTAAGT
TATCTGTTTTGATAAAACACTCTTCCCATACGTTCTATGAATGGGAAAAGAATGTCACGTTTCTCTCTTTCAA
GTATATGAAGCCTTTGCGTATTTAAGTGTTTTCTATGAGAACTTCTAGTTTCCGTGAATGAATGTGTCTTTCA
CATTCTAGGAAGACCAAAGCGGACTGTTTCCCATTCAACTGCAATAAGCCAAAGGAGATGCAGTTTTGCTG
ACTAAACCTTGTAACCACGTGAATATTTCAAATTCACTGCATGAAAGAGAAACCAAAATGCAGTTTTCTCA
TTCTAGTTGATTAACTTACTCTAGTTGGTTAAGTGGTCTGTTTTGATAAAACACTCTTCCCACACATTCTGTG
AATGGGAAAAGAATGTACAGTTTCTCCCTTTCAAGTAAATGAGGACTTCAGGTATTCTGATGTTTTCTATGA
GAACTTTGGGTTACCAGGAATGAATGTGTCTTTCACTTTCTGGGAAGAACAAGGCAGATTGTTTCCCGTTCA
ATTACAAGACAGCAAAGGTATGTAGTTTTGCTGACTAAACCTTATGTTGTAAGCATGTACATGTTTCCA
>BMBL01099827.1_15
ATTCACTGCATGAAAGAGAAATCACAATACAGTTTCTTTCATTCTAGTTGATTATGTCAGAGGGTATTATCT
GTTTTGATGAAACACTGTTCCCACACGTTCTCTCAATGAAAAAAGAATGTCAACTTTCACACTATCAAGTAC
GGGAGTCTTCTGGGTTTTCAGATGTTTTCAATGGGAACTTTGCGTTACCGGAAATGAACGTGACGTTCACAT
TCTAGGAAGACAAAGGCAGATTGTTTCCAGTTCAATTAAAGGACAGCAAAGACAATGTTGTTTTGCTGACT
AAACCTTGTGTTGTAACTGCATGCATATTTCCAATTCGCTGCACGTAGGAGAAACCAAAATATTTTCTCTCA
TTCTGATTGATTAAGTCAGAAGGATTAACTTATTGTGTTTGAAAATAACTCTGTGTTACTTGCTGGAGAATG
TTGCCTATACATTCTCTACCAGGGTGAAGAATTTAACTTTTCTCCCATTCAAATACATGGAGCCATATGAGG
TTTAGATGTTTCCAAGAAGCACTCTGTGCTGCCTGGCCATAAATGTGCTTTACAGACAAAATATCTGAAGG
AGAAAACAATTCGAGTTTCTCCCGTTCAAGTATAGGACGGTAATGGAGAAGTAATTTTGCTAACTAAACAC
TGTCACCGGAAGTGCACATTCCCA
>BMBL01099827.1_16
ATTCACTTCATGAAAGTGAAAAAAAAATACAGTTTTCTCATTTTAGTTGATTTAACTTAATCTAGTTGATTAA
GTTGTCTGTTTTGTTAAAACACTGTTCCCACAGGATCAAAAACATTTCTTATATATTCTCTGACTGGGAAAA
GAATGTCAAGTTTCTTCCTTTCAACTACGTGATGCTTCTGGGTTTTCAGATGTTTTCTATGGGAACTCTGTGT
TACCAGAAATTAATGTGCCTTTAAAACTCTAGGAAGAAGAAGGTATGTTGTTTCCTGTTCAACTACGGGAA
GGCAAAAGCGATGTAGTTTTGCTGAGTAAACCTTGTGTTGTAAGCACGTGCATGTTTCCAATTCAGTGCATG
ACAGAGAAACCAAAATACAGTTTTCTCATTCTAGTTGATTAATTTAATCTTGTTTTGAGAAAACACTGTTCT
CGTTCTCTGGATGGGAAAAGAATACCAAATTTCTCCCTTTCAAGTACATGAAGCCCCAGTGTTTTCAGATAT
TTTCTATGGGAACTTTGGGTTATGGGGAATAACTTGGCCTTTCACATTCTAAGAAGGACAAGGCAGATTGTT
TCCTGTTCAACTACAGGTCAGCGAAAGCCATGTAGTTTTGCTAAGCAAACACTGTGTTACCAGAAAGTGCA
TGTTCCCA
>BMBL01099827.1_17
ATTCACCACATGGAAGGGAAACCAAAATACAGTTTCTCTTGTTCTAGTTGATTAAGTCAGAAGGTGTTATCA
GTTTGATAAAACACTGTTCCCACATGACCGAGAACCACTTTTATACCTTCTCTGAATGGGAAAAGAATGTC
AAGTTTCTCCTTTTCAAGTATGTGAAGCCTCTGAGTTTTCAAATTTTTTCAATGGAAACTTTGGGTTATGGAG

AATGAACATGCTTTTCACATTCTAGGAAGAACAAGGCAGATTGTTTCCTGCTCACCTACAAAACTGCAAAA
ATGATATACTTTTGCTGACTGAAACTTTTGTTGTAAACACGTGCATGTTTCCAATTCACTGCAAGAAAGAGA
AACCAAAATACAGTTTCTCATTCTGGTTGATTAAGTCAGAAAGATTAAATTACTGTGTTTGAAAACTTCTGT
GTTACTTGCACGAGAAGGTTGCCTATACTTTCTCTACCAAGGTAAAGAATTTAACTTTTTTCCCAATCTAAC
TGGAGAAGCCACACGGGATTTAGATGTTTTGAAGCAGTATTCTGTGTTTCCCAGGCTTGTATGTGCCTTAGG
GACAAAGTCTCTGAACAAGAAAACAATGCGAGTTTCTTCCATTCAAGTATAGGATGGCATTAATGATGTAG
TTTTGCTCAGCAAACATTATGTTACCAGACTGAGCACTTTATCA
>BMBL01099827.1_18
ATTCACTGTATGAAAGAGAAACCAAAATACAGTTTCTCTCATCTGCTTGATTAAGTCACATGGTATTATCTG
TATTGATAAAACTCTGTTCCTACATGATGGAGGATCACTCTTATACATTTTCTGAATTTGAAAATAATGTCA
AGTTTCTGACTTTCTAGTATGTGAGTGTTTTGGTTTTCAGGAATGAATGTGCCTTTCATAGTCTATGAAGAAC
AAGGGAGATTGTTTCCCGTTCAACTACAGGACAGCAAAAACTATGTAGTTTTACTAATTAAACCTTGTGTTG
TAAGCACGTGCATGTTTCTAATTCATCACATGAAAGATAAACCAAAACACAGTTTCTCTCATTCTGGTTGAT
TATGTCAGAGCGCTGACTTACTATGTTTGAAAACACTCTGTGTTTCATGCATGAGAATGGTTTGTATGCATT
CTCCACCTGGGTAAATAATTTAAATTTTGTCCCTTTCAAATAGATGAAACCATGCAGTGTTTAGATGTTTTC
AAGAAGTACTCTGTGCTGCCTGACCATGAATGGGCCTTACAGATAGAATCTCTGAACCAGAAAACAATGCA
AGTTTCTCCTGTTCAAGTATAGGACAACAATGTAGTTTTGCTAAGTAAATACTCTGTTACCAGAATGTGCAA
GTTCCCA
>BMBL01105564.1_1
ATTCAGTGCATGATATAGAAAGCAAAATACAGCGTTCTCCTTCAAGCTGGTTAACTTAATCCACTGAAGTTT
TCAGTTTTGTTAGAAGAAACTGTCTGCCCACCACCAGCTGCTTTCTCAACGGTTCTCTCAGTGGGATAAGAC
TGGAGATTGCCTGCCTTGCAAGCATTTGAATCCCCTGGTTATCAAAGCTATCTTTGCCTAACAGGGAACGAA
GGAACTTTTGGGATTCAAAGTCAAAGAGGTAGATTGTTTGCTTTTAGCGAGAGGATGTTAGAAGAAGTTCA
GTTTGGCTGAGCAAAGCTTGAGTGCTTCACGTGAGCATGTAGCCTCTTCCCTGCCAGAGAGAGGAAGCAAA
AGCCAGTTGCTCTCAGTCTGGGGGATGAAGTGAGAAGGATCCACATACTGTGTTTGAAAATCCCTCTGTGT
TACATGCCCAAGAATGTTGCCTGTGCATTGTTGACCTGGATAAAGAGTTTGACTTTTCTCCCTTTCGAGTAC
ATGAAGCCCTGCTATGTTTACAGCTTTCTGGGAGGCACTCTGTGCTGCCCAGATGAGAATGCCGCCTTGCCG
GAGGTATCCCTGAAGGAGGGGAGAAAGCGCGTTCTCCCCTTCTGCTATACAACAGTGGCAGCACTGTAGTT
TTGCTAATTAAGCACTATATTAACAGCACGTACATGTTCATA
>BMBL01105564.1_2
ATACAGTGCCTGAAGGAGAAAGCAAAATACACTGCTCTCGTGGAAGCTGGTTAACGTTTCTGTTTTGGTAG
AAGAAACAGTCCTCACACCACCAGCTGCTTCCTCAAACATTCTCTCAATGGGATAAGACTGGCGAGTGTCT
GCCTTTCAGGTACTTGAATCCCCTGCTTTTCAAAGGCAACTTTGCATTACAGGGAAGGAAGGTAACATTCGC
ATTCTAAGAAAAACAGGCAGATTGTTTGCCTTCAGCTAGAGGACCGCAGAAGCAGTTCAGTTTTGCTGACT
AAAGCTTGCTTGGTTAACACGAGCATGTATCCTATTCCCCGTAAGAAATAGAAACGGAATGGCAGTTGCTC
TCATTATGGGGGAATGAAGTGAGAAAGATTGACTTACTGTGTTAGAAAATCCCTCTTTATTATATGCTCAGA
AATGTTGTCTGTGCATTGTTTACCTGGGTAAAGACTTTGACTTTTCTCCCTTTCCAGTACATGAAGCCCTGCG
GGGTTTACAGCTTTCCGGGAGGCACTCTGTGCTGCGCCACCGAAAATGTAGCTACTGAGTCTTTAAGAAAA
GAACTGTTGAGTATTATCGTATTATAAAATGATGTATATAATTCACACCATGATTTT
>BMBL01043776.1_1
ATTCACTGCATGAAAGAGAAAGTAAAATAGTGTTCTCATTCAAGTTGGTTAACTTAATCAAGTGGATTGTTA
TGAGTTTTGATAAAACACTCTCCTCACCCCATCCACAAGCTTTCTCAAACATTCTCTCAGTTGGAATGATGA
GACTCTCTCTTTCAAGTACTTGAATCCCTCAAGTTTTCAGAGGGAGCTCTGTGTTACCCGGAATGAAGGTAC
CTTTCACATTTTAGGAAGAACAAGGCAGATTGTTTCACTTTCAGCTAAAAGACAGCAGAAACAGTGCAGGT
ATGCTGACTAAACATTGTGCTGTAAGCACTTTCATGTTTCCAATTCACCACAAGAAAGCAAAACCAAAAGA
CAGTTGCTCTCAGACTGGTAGATGAAGTCAAAAGGATTCACTTACTATGTTTCAAAATCCCTCTGTGTTATG
AGCCCAAGAATGTTGCCTATGCATTCTTCACCTGGATAAAGAGTTCGGATTTTCTCTCCTTCGAGTACGTGA
AGACCTGTGGGCTTAAGAGGTTTCTGGGAGGCCCTCTGTGCTGCCCAGCCAAGAACGCAGCCTTATAGATG
GTATCTCTAAAAGAGAAAACAATGTGCGTTACACTTATACAGGTATAAGAGAGCAGTAGACATGTGATTTG
CTAAGTAAACACTATGTTAACAGGGCATGCATGTTCCCA
>BMBL01043776.1_2
ACTCACTGCAAGAAAGAGAAAGCAAAATACAGTGTTCCCATTCAAGTTGGTTAACATAATCAAGTAGATTA
ACTTATGAGGTTTGATAAAACACACTCCTCACACCATCCACAAGCTTTCTCAAACATTCATTCAGTGGGATA
AGAATGGTGAATGTTGCCCTTTCAAGTACTTGAATCCGCCAGGTTTTCAGATGTTCTGTAACGGAGCTGTGT
GTTAGCCTGAATGCAGGTACTTTCCGCATTCCAGGAAGGACAAGGCAGATTGTTTCCAGTTCAGCTAAGGG
ACGATAAAAGCAGTGCAGGTTTGCTGAGTAAACCTTCTGTTGTGAGGACTTGCTTGTTTCCAATACACTGAA
AGAAAGAGAAATCAAAAGATGGCTGCTCTCAGTCTGGTGGATGAAGTCAGAAGGATTCACTTACTGTGTTT

GAAAATCTCTCTCTGTTCCATGCCCAAGACTGTTGCCTGTGCATTCTCCACCTGGGTAAAGAGCTCGACTTT
TCTCCCCTTTGAGTACGTGAAGCCCTGCAGGGTTCAAGGCTTTCTGGTGGGCACTCTATGCTGCACAGCCAA
GAAACCAGCCTTACACAAAATGTCTCTGAAGGGGAAAAGAAAGCAAATTTCTCCCATTCTGGTATACCGCA
GCAGTAGAGATGAAGTTTTGCTAAATAAACACTATGTTAGCAGGACGTGCATGTTCTCA
>BMBL01043776.1_3
ATTTCCTGCAGGAAAGAGAAAGCAAAATACAATGTTCTCATTCAAGTTGGCTAAGTTCAGTTCATCAAGTT
ATCAGTTTTGATAAAACACAGTCCTCACACCATCGAGAAGATTTCTCAAACATTGTTTCAGTGGGATAATTC
TGGTGAGTCTCTTGCTTTCAAGTGATTGAATCCCCTGGTTTTCAAGGGGGACTTAACAGAGAATGAAAGTAC
CCTTTGCATTTAGGAAAAACAAGTTAGATTCTTTTGCCTTCAGCTAAGGCATGGCAGAAGCAGTTCAGTTTG
CCTGACTAAACCTAGTGTTGTAAGCATGTGTAAGTTTCCTATTAACTGCAAGAAAGACAAAGCCAAAGACA
GTTGCTCTCAGTCTGCTAGATGAAGTGGGGATTCGCTTACTTTGTGTTGGAAAGTCCCTCTGTGTTACACAC
CCATGAATGTTGTCCAGGAATTCTCTACCTGGGTAAAGACTTTGATTTTTCTCCCCTTCCAATACGTGAAGC
ACCGCAGAGTTTACAGCTTTCCGGAGGAACTCTATGCTGCCCGGCTGAGAGAGTGGCCTTACACAGCGAGT
CTCTGAAGGGGAAAAGAAAGCGCGTTTCTCCCATTCTGGTATACCACAGCAGTGGAGATATAGTTTGGAGA
AGTAAACACTACGTTAGGGGGAAGTGCATGTTCCCA
>BMBL01043776.1_4
ATTCACCCCAGGAAAGAGGAAGCAAAATACGGCGTTCTCCGTCAAGCTGCTTAACTGAATCTACGGAAGTT
TTCAGTTTTGTTAGAAGAAACAGTGTGCCGACCAGTAGCTGCTTTCGCAAAGGCTCGCTCAGTGGGAGAAT
ACAAGAGAGTGCCTGCCTTGCAAGTATTTGAATCCCCTGGTTATCAAAGCTATCTTTGCCTAACAGGGAAC
GAAGGAACTTTTGGGATTCAAAGTCAAAGAGGTAGATTGTTTGCTTTTAGCGAGAGGATGTTAGAAGAAGT
TCAGTTTGGCTGAGCAAAGCTTGAGTGCTTCACAGGAGCGTGTAGCCTCTTCCCTGACAGAAAGAGGAAGC
AAAGCCAGTTGCTGTCAGTCTGAGGGATGAAGTGAGAAGGATTCACTACTGCGTTTGAAAATCCCTCTGTG
TTCCATGCCTAAGGAAGGAAGTTGCCTGTGCATTGTGTACCTGGGTAATGGGATCCACTTTTCTCCCTTTCA
AGCAGGTGAAGCCATTCTGGGTTTAAAGCATTCCTGATGACATTCTGTGCTGCTGGGCCGTGAATGCAGCTT
TACAGACGCTATCCCTAAAGGAGAAGAGAAACCTTTCTCCAGTTCTGGTATACCACCGTGGCAGAGCCGCC
GTTTTGCTGTGTCAAAAGTATGTTAGCAGCACTTATAACTTCCCA
>BMBL01006510.1_1
ATTCACCGTATAAAAGAGAAAGCAAAATACAGCGTTCTCCTTCAAGCTTCTTTACTGAATCTACTGAAGAA
AGAGTCCACCCACCACCAGCTCTTTGCTCAAATGTTCTCTCTCAGTGGGAGAAGACTGGAGAGTGTCTGCCT
TGGAAGCATTTGAATCTCCTGATTTTCAAAGGGAACTTTGCATAACAGGGAATGAGTTACTTCTGGCATTCA
AAGCGAAACAGGCAGATTGTTGGCCTTCAGCTAGAGGAAATGCAGAAGAGGTTCAGTGTTGCTGACTAAA
CCTTATTTGCTTAATGTGACCACGTAGCTTATCCTTGCCAGAAGGAGAAAGCAAAGCAAGTTGCTGTGAGT
CTGGAGGATGAAGTGAGAAGGAGTCACCTACTGTGTTTGAAAATTCCTATGTGTTACATGCCTAAGAAAGT
TGCCTCTGCATTGTCTCCCTCGGTAAAGGGATCCACGTTTCTCCCTTTCAAGTATGTGAAGCCATTCAGAGT
TTACAGCTTTCGAGGAGGCATTCTGTCCTGCGCGGCCGAGAATGCCACCTTTCAGACGGTATCACAGAAGG
GAGTGAAACCGTGTTTCTCTCGTTCTGGTATAGGACAGCGGTGGAGCCACAGTTTTGCTGAGTAAAGACTA
TGGTACCAGCAAGTGTAAGTTCCCA
>BMBL01061436.1_1
ATTCAGTCTATTAAAAATGAAGGCAAAATGCTGTGTTCTCATTCAAGCCTGTTTCTTTAATGAACTGAAATT
TTCACTTTTGTTAGAACAAGCAGTCCTGTCACCATCAGCTTCTTTGTGAAACATTCTCTGAATGGGTGTAAC
TGGCCTTTCAAGTACTTGGATCCTGTGGTTTTCGAATGGAAAGTTTAATTATAGAGATTGAAGGAACACTTT
GCATTGAAAGGAGAACAGTCAGATTGTTTGCCTTCACCGAGAGGACAGCACAAGCAGCTCAGCTTGTCTGA
CTAAAGCTTGTGCGGTTAATATCAGCATGTATCCTATTCTGTTCAAGAAAGAGAAACCAAAAGACAGTTGC
TCTCAGGCTGTGGGAATGAAGTGAGAAGGATTCACTTACTGTGTTAGAAATTTCCTCTGTGTTACATGCCCA
AGAACTTTCCCTGTGCTTTTTTGCCCTGGGTAAAGTGTTTGACTTTTCTCCCTTTCGAATACGTTTATCCCTG
CGGGGTTTACCGCTTTCCGTGTGGCTCTCTTTGCTGCGCTGCGGATAATGCCGCCTTGCATTCCGTATCGCTG
AAGGGAAGAAAAAGCGGATTTCTCCCATTCCCATATACGGCAGCAGCAGTGCTGTACTTTTGCTAACTAAA
CACTATGTTAGCAACATTTGCATGCTCCCA
>BMBL01061436.1_2
ATTCACTGTATGAGAAAGAAAAATACCGTGTTCTCATTCGACCCTGTATACTTAATGAACTGAAGTTTTCCG
TTTTGGTAGAACAAGCAGCCCTGACACCACCAGCTACTTTCTGAAACACTTTCTCAATGGGATAAGACTGA
CCAGTTTCTACCTTTCAAGTACTTGAGTCCTCTGGTTTTCAAATGGAACTTTGCCTTACAAGCAAAAATGAT
AACTTTCGCATTGTAAGGAAAATAGGCAGATTGTTTACCTTCAGCTAGAGGATAGCAGAAGCAGTTCAGGT
TTGCTAACTAATGCTTGTGTGGTTAACATGAGCATGTTTCCTATTCCCTGCAAGTAACAGAAACCTAAAGAC
TCTTGCTGTCAGTCTGGGTAATGAAGTGAGATGGAGTCACTTATTGTGTTTGAAAATCCCTCTCTGTTACAG
GCCCAAAAATGTAGCATGTACATTGTCTACCTGTGTAAAGAGTTTGCCTTTTCTCCCCTTCGAGTACGTGAA
GCCCTGCGGGGTTTACAGCTTTCAGGGAGGCCCTCTCTGCTGCACGGCGGTGAATGCCTTCTTACTGACATC

TTCACTGAAGAAGAGAAAACTCGTTTCTCCAGTTCTGGTATATGAGAGCGGCAGCAGTGTAGTTTTGCTAA
CTAAACACTGTGGAAGAAACACGTGCATGTTTCAC
>BMBL01061436.1_3
TTCTCTGCATGAAGAAGAAAGCAAAATACCGTGTTCTCATTCAAGCCTGTTTACTTAATCAACTGAAGATTT
TCAGTTTCGGTAGAGCCAACGGTCGTGACATAACCAGACACTTTCTGAAATATTTCTCAATGGGATAAGA
CTGGTGATTGTCTGCTTTTCCAGTACTTGAATCCCCTGGTTTTCAAATGGAACTTTGCCTCACAGGCAAAGA
AGGTAACTCTCATTCTAAGGAAAATAGGCAGATTGTTTGCCTTCAGCTAGAGGACAGTAGAAGCAATTCAG
TTTTGCTTACTAAAGCTTGTGTGGTTAACTTGAGCATGTTTCCTATTCCCTGCAAGAAAAAGAAACCAAAAG
ACAGTTGCTCTCAGTCTGAGCAATGAAGTGACAAGGTTTCACTTACTTTGTTTGAAAAATCCCTCTGTTTTA
CATGCCCAAGAAAGTTGCCCCTGCGTTGTTTACTGGGGTAAAGAGTTTGACTTTTCTCCCATTCGACCACGT
GAAGACCTGTGGGGTTTACTGCTTTACAGGAGGCACTCTGTGCTGCTAGGGAGAAAATGCCACTTTACAGA
CTCTATGTGTGTGGGAGAAGAGAAAACGCATTTCTCCCTTTCAGGTATATGACAGCAACAGCTGTGTTGTTT
TGCTAACTAAAGACTATGTTAGTAGCACATGTATCTTCCCA
>BMBL01061436.1_4
AATCAATGTATGAAAAAGGAAGCTAAGTACAGTGTTCTCACTGAAGCCTGTTTACTTAATCAACTGAAGTT
CTCGGTTTTGGTAGAGCAAACAGTCCTGACACCACCAGCTACTTTCTGAAACATTCTCTCATTGGGATAAGA
CTGACCAGTGTCTACCTTTCTAGTACTTGAATATCCTGGTTTTCAATTGAACTTTGCACTGTAGGGAATGAA
CATTTTGCATTAAAAAAAAACAAGCAGATTGTTTGCCTTCTGCTAGAGAACGACAGAAACAATTAATTGTT
GCTTACTAAAGCTCATGTGGTTAACATGAGCATGTTTCCTATTCCCTGCAAGAAAGACAAACCAAAAGACA
GGTGCTCTCAGTCTGGGGAATGAAGTGAGAAAGATTCACTTTGTTTGAAAATCCCTCTATGTTACATGCCCA
AGAAAGTTGCCTGTGCGTTGTCTACCTGGGTAAAGAGTTTGACTTTTCTCCCCTTTGAGTACTTGAAACCCT
GAGGGGTTTACAGCTTTCTGGGAGGCACTCCGTGCTGCACGGCCGAGAATGCTGCCTTACAGATGTTATCG
GTTGAAAGGGAGAAGCTTCATGTTCTTTTCACACTCAGATAATGTATAAGAAAAGTTTTTCACCG
>BMBL01061436.1_5
ATTTCCTGCATGAAAGAGAAACCAAAATACAGGCTTTTCATTTTAGTAGTTTAGCTTAATCAAGTTGATTAA
TTTATATGTTTTGATAAAACACTGTTGCCACGCCATCGAAAACCATTCCTACACATTCTCTGGGAAAACAAT
GTCAAGTTTCTCCCTTTCTCGTACTTGAAGCCCCTGGGCTTTCAGATGTTTCTATGGGATCTCTGGTTTACCG
AGAACCAATATGCCTTTCACATTCTAGGAGAAAAGAGGCTAATTGTTTCCCATTCAACTACAGAATGACAA
AAGCGATGTGGTTTTGCAAACTAAAGCTTGTGTTGTCTACAAGCACGTTTGTAATTCACTGCTCGAAAGAGA
AATCAAATATTGTTTCTTTTATGCTGCTTTAGTAAGTCTGAAAGATTAACTTCCTGTGTTTAAAAAACCCTCT
GTGTTACATGCATGAGAGTGTTGTCTTTACTTTCTCTACCAGGGTAAAGGGTTGAACTTTTCTCCCTTTCAAA
TACATGAAACCCTACGGGGTTTAGATGTTTTCAAGCAGTACTTGGTGCTTTCCGTTAGTGAATGTGCCTTAC
ATACAGAATCTCTGAAGGAGAAAACAATGCTTGTTTCACCTGTTCAAGTACAGGACCACAGCAGACATGCA
GTTTCTCTAAGTAAACACTATGTTACCAGAACGTGCGTGTTCCCA
>BMBL01061436.1_6
ATTCACTGCATGAAAGAGAAACCAAAATACAGGTTTTTCATTTTAGTAGTTTAGCCTAATCAAGTGATTAAG
TTATCTGTTTTGATAAAACGCAGTTGCCACAAGATAGAATACCATTCTTACACATTTTCTGAATGGGAAAGC
AATGACAGTTTTCTCCCTTTTTGGCATTTGATGCCTCTGGGCTTTCAGATGTTTTCTATGGGCTCTCAGGTTT
ACCAGAAATGAATGTGCCTTTCAAATTCTAGGAAGAACAAAGCTAATTGTCTCCCATTCAACTACATAACG
GCAAAAGCGATCTGATTTTGGTGACTAAATCTTGTTTCATTCATCCACATGTTTCTAATTCACTGCTCAAAA
GAGAAACCAAAATACTGTTTCTTTCATACTGCTTGAGTAAGTCAGAAGGATTAACTTACTGCGTTTGAAAAC
TCCTCTGTGTTAAATGCACAAGAGTGTTGCCTATACATTCTCTACCAAGGTAAAGTGCTGAACTTTTCTCCC
ATTCAAATACGTGAAGCCATATGGGGTTTAGATGTTTTCCAGCAGTACCCTGTGCTTTCTATTCGTGAATAT
GCCTTATAGACAGAATCTTTGAAGGAGAAAACAATGTGTGTTTCTCCCATTCAAGTACAGGATGGCGACAC
AGATGTAGTTTCATTAAGTAAACATGGTGTTACCAGAAAGTGCATGTTCCCA
>BMBL01061436.1_7
ATTCACAACATGAAAGAGAAAGCAAAATGCAGTTTCTCTCATTCAGCTTGAGTATCTCAGAAGGATTAACT
CACTGTGTTTGAAAAGCCCTCTGTGTTACATGCATGAGAATGTTGCCTATACATTCTCTACCAGAGAACTTT
TCTCCCATTCAAACACGTGAAGCCATATGGGGTTTAGATGCTTTCAAGCAGTACTCTGTGCTTTCTGTTCAT
GAACGTGCCTTACAGACAGAATCTCTGAAGGGGAAAACAATGCATGTTTCTCCGGTTCAAGTACAAGACGA
CAATAGAGATGCAGTTTCACTAAGTAAACACTGTGTTATCAGAATGTGCATGTTCCCAATTCACTACATGA
AAGAGAAAGCAAAATACAATTTCTCACATTCAGCTAGAGTAAGTCAGAAGAATTAAATTACTGTGTTTGAG
AAACACTGTGTTACAGGCATGAGAACATTGCCTATATTTTCTATATGTGGGTAAAGAATTGAACTTTTCTCT
CACTCAAATACGTGAAGCTATGCAGACTTCATATGTTTTCAAGTTGCACTCTGTGCTGCTCGGCTGTGAATG
TGCCTTACACACAGAATCTCTGACGGAAAAATAATGGGAGTTTCTCCCGTTCAAGTATAAGACAGTAATAG
CGACGTCGTTCTGCTAAGCACTGTGTTACCAGAAAGGGCATGTTCCCA
>BMBL01082959.1_1

ATTCACTGCCTGAAAGAGAAAGCAAAACAGTGTTCTCTTTCAAGTTGGTTAACTTAATCAATTGATTAAGTT
ATCAGTGTTTATAAAAACACAGTTCCTGCACAATCCACAAACTTTCCCATGCATTCTCTAAATGGAAAAAG
AATATCCAGCTTCTACCTTTCAAGTACTCAAATCCCCCGAGTTTTCAGATGTTTCCTAAGGGAACTTTGCAT
TACCGGGAATAAAGGTGGGTTTCACACTGTAGGAAGAACAAGGCAGATTGTTTCCAGTTCACCTAAAGGAT
GGAAAAAGTGATGTAGTTTTACTGACTAAAGCTTGTGTTGTAAGCATGGGCATGTTTCCCATTCACTGCAAG
AAAGAGAAACCCAAATACAGTTCCTCTCATTGTGGTGGAATAAGTCAGAAGGATTAAGTTATTGTGCTTGA
AAATCCCTGTGCTACATGCACAAGAATGTTGCTTATGCATTCTCCACCTGGGTAGAGTTTAACTTTTCTCAC
ATTCGAGTACGTGAAGCCATGTGGCATTTAGATGTTTCCAGTAGGCGCTCTGTGCTGCCCGGCCTCAAATGG
GCCTTACAGACAGAATTTCCAAAGGCGAAAACAATGCGAGTTTCTGCCGTTCAAGTATAACGCAGCAACAA
AGATGTCATTTCGCTAAGGAAACACTATGTTTAGCAGAACTTGCACGTACCCA
>BMBL01082959.1_2
ATTCACTGCATGAAAGAGGAAGCAAAATACAGTGTTCTCATTCAAGTTGGTTAACTTAATCAATTGATTAA
GTTATCAGTTTTGATAAAACACTGTTCCCACATGATCAATAAACTTTCTCATAATTCTCTGAATTGGAAAAG
AATGTCAAGGTTCTCCCTTTCAAGTACTTGAATCCCCCAGGTTTTCAGATGTTTTCTAAGGGAACTTTGCGA
TACCAGGAATGAATGTGTTTCACATTCTAGGCAGAACAAGACAGATTGTCTCCCATTCAACTAAAGGATGG
CAAAAGCAAGGTAATTTTGCTGACTAAACCTTGCGGTTTATGCTCAGGCATGTTTCCCATGCACTGAAAGA
AAGAGAAACCAAATTACAGATTCTCTCATTCTGCTGGAATAAGTCAGAAGGATTAACTTACTGTGGTTGAA
AATCTCTTTGTGTTACTTGCAAAAAATGTTGCCTATACATTCTCCACCTGGGTAAAGAGATAAGCTTTTCTC
CCATTTGAGTACGTGAAGGCATGTGGGGTTTAGGTGTTTTCAGTAGGCACTCCGTGCTTCCCAGCCATGAAT
GTGCCTTACAGACAGTATCTCTAAAGCAGAAAACAATGCGAGTTTCTCCCATTCAAGTATAGGACAGCAAT
AGAGATGTAGTTTTGCTAAGGAAACACTATGTTACCAGCATGTGCACGTTCCCA
>BMBL01041892.1_1
ATTCACTGCATGAAAGAGAAAGTAAAATAGTGTTCTCATTCAAGTTGGTTAACTTAATCAAGTGGATTGTTA
TGAGTTTTGATAAAACACTCTCCTCACCCCATCCACAAGCTTTCTCAAACATTCTCTCAGTTGGAATGATGA
GACTCTCTCTTTCAAGTACTTGAATCCCTCAAGTTTTCAGAGGGAGCTCTGTGTTACCCGGAATGAAGGTAC
CTTTCACATTTTAGGAAGAACAAGGCAGATTGTTTCACTTTCAGCTAAAAGACAGCAGAAACAGTGCAGGT
ATGCTGACTAAACATTGTGCTGTAAGCACTTTCATGTTTCCAATTCACCACAAGAAAGCAAAACCAAAAGA
CAGTTGCTCTCAGACTGGTAGATGAAGTCAAAAGGATTCACTTACTATGTTTCAAAATCCCTCTGTGTTATG
AGCCCAAGAATGTTGCCTATGCATTCTTCACCTGGATAAAGAGTTCGGATTTTCTCTCCTTCGAGTACGTGA
AGACCTGTGGGCTTAAGAGGTTTCTGGGAGGCCCTCTGTGCTGCCCAGCCAAGAACGCAGCCTTATAGATG
GTATCTCTAAAAGAGAAAACAATGTGCGTTACACTTATACAGGTATAAGAGAGCAGTAGACATGTGATTTG
CTAAGTAAACACTATGTTAACAGGGCATGCATGTTCCCA
>BMBL01041892.1_2
ACTCACTGCAAGAAAGAGAAAGCAAAATACAGTGTTCCCATTCAAGTTGGTTAACATAATCAAGTAGATTA
ACTTATGAGGTTTGATAAAACACACTCCTCACACCATCCACAAGCTTTCTCAAACATTCATTCAGTGGGATA
AGAATGGTGAATGTTGCCCTTTCAAGTACTTGAATCCGCCAGGTTTTCAGATGTTCTGTAACGGAGCTGTGT
GTTAGCCTGAATGCAGGTACTTTCCGCATTCCAGGAAGGACAAGGCAGATTGTTTCCAGTTCAGCTAAGGG
ACGATAAAAGCAGTGCAGGTTTGCTGAGTAAACCTTCTGTTGTGAGGACTTGCTTGTTTCCAATACACTGAA
AGAAAGAGAAATCAAAAGATGGCTGCTCTCAGTCTGGTGGATGAAGTCAGAAGGATTCACTTACTGTGTTT
GAAAATCTCTCTCTGTTCCATGCCCAAGACTGTTGCCTGTGCATTCTCCACCTGGGTAAAGAGCTCGACTTT
TCTCCCCTTTGAGTACGTGAAGCCCTGCAGGGTTCAAGGCTTTCTGGTGGGCACTCTATGCTGCACAGCCAA
GAAACCAGCCTTACACAAAATGTCTCTGAAGGGGAAAAGAAAGCAAATTTCTCCCATTCTGGTATACCGCA
GCAGTAGAGATGAAGTTTTGCTAAATAAACACTATGTTAGCAGGACGTGCATGTTCTCA
>BMBL01041892.1_3
ATTTCCTGCAGGAAAGAGAAAGCAAAATACAATGTTCTCATTCAAGTTGGCTAAGTTCAGTTCATCAAGTT
ATCAGTTTTGATAAAACACAGTCCTCACACCATCGAGAAGATTTCTCAAACATTGTTTCAGTGGGATAATTC
TGGTGAGTCTCTTGCTTTCAAGTGATTGAATCCCCTGGTTTTCAAGGGGGACTTAACAGAGAATGAAAGTAC
CCTTTGCATTTAGGAAAAACAAGTTAGATTCTTTTGCCTTCAGCTAAGGCATGGCAGAAGCAGTTCAGTTTG
CCTGACTAAACCTAGTGTTGTAAGCATGTGTAAGTTTCCTATTAACTGCAAGAAAGACAAAGCCAAAGACA
GTTGCTCTCAGTCTGCTAGATGAAGTGGGGATTCGCTTACTTTGTGTTGGAAAGTCCCTCTGTGTTACACAC
CCATGAATGTTGTCCAGGAATTCTCTACCTGGGTAAAGACTTTGATTTTTCTCCCCTTCCAATACGTGAAGC
ACCGCAGAGTTTACAGCTTTCCGGAGGAACTCTATGCTGCCCGGCTGAGAGAGTGGCCTTACACAGCGAGT
CTCTGAAGGGGAAAAGAAAGCGCGTTTCTCCCATTCTGGTATACCACAGCAGTGGAGATATAGTTTGGAGA
AGTAAACACTACGTTAGGGGGAAGTGCATGTTCCCA
>BMBL01041892.1_4
ATTCACCCCAGGAAAGAGGAAGCAAAATACGGCGTTCTCCGTCAAGCTGCTTAACTGAATCTACGGAAGTT
TTCAGTTTTGTTAGAAGAAACAGTGTGCCGACCAGTAGCTGCTTTCGCAAAGGCTCGCTCAGTGGGAGAAT
ACAAGAGAGTGCCTGCCTTGCAAGTATTTGAATCCCCTGGTTATCAAAGCTATCTTTGCCTAACAGGGAAC

GAAGGAACTTTTGGGATTCAAAGTCAAAGAGGTAGATTGTTTGCTTTTAGCGAGAGGATGTTAGAAGAAGT
TCAGTTTGGCTGAGCAAAGCTTGAGTGCTTCACAGGAGCGTGTAGCCTCTTCCCTGACAGAAAGAGGAAGC
AAAGCCAGTTGCTGTCAGTCTGAGGGATGAAGTGAGAAGGATTCACTACTGCGTTTGAAAATCCCTCTGTG
TTCCATGCCTAAGGAAGGAAGTTGCCTGTGCATTGTGTACCTGGGTAATGGGATCCACTTTTCTCCCTTTCA
AGCAGGTGAAGCCATTCTGGGTTTAAAGCATTCCTGATGACATTCTGTGCTGCTGGGCCGTGAATGCAGCTT
TACAGACGCTATCCCTAAAGGAGAAGAGAAACCTTTCTCCAGTTCTGGTATACCACCGTGGCAGAGCCGCC
GTTTTGCTGTGTCAAAAGTATGTTAGCAGCACTTATAACTTCCCA
>BMBL01022471.1_1
ATTCAGTGCATGAAAAAGAAAGCAATATGCAGTGTTCTCTTTCAAGCTGCTTCACTTAAACAACTTAAGTTT
TCAGTGTTGGTAGAACAAAGCGTCCTCATACCACCGGCTGCTTTCTCCACCTTTCTTGCAATGGAAGAATAC
TGGCGAATGTCTGCCTTTCAAGAGATTGCATCCCCTGGTTTTCAAAGGGAACTTTGCAGTACATGGAATGA
AGGTAAGATTTGCATTCTGTGAAAAACATGCAGATTTTTTGCCTTCAGCTAGAGAAGGGCGGAAGTGGATC
AGTTTTGTTGACTATATCCTGTGTGGTTAACACGAGCAGGTATCTTATTCCTTCCAAGACAGAGAAACCAAA
AGACAGTTGCTCTCATTCTGGGGGATGAAGTGAGATGGTTTCACTTACTGTGCTTGAATATCCCTCTATGTT
ACATGCCCAGGAAATTTTCCTGTCGATTGTCTCCCTGAGTAAAGTGTTTGAATTTTCTCCCCTTCGAGTACA
AGAGGCCCTGGGGGTTCACAGCTTTCCCGAACCACTAGTTGCTGCGCGGCCTAGAACACCGCCTTATAGAC
TGTATGCCTGAAGGAGAAGAGAAAGCGTGTTTTTCCTGTTCTGGTAAGACAGTGGCAATGCTGTAGTTTTGC
TAACTAAAGACTATATTAGCAGCACTTGCAAGTTCCTA
>BMBL01022471.1_2
ATTGAAAGCATGAAAGATAAAGCAAAACACAGTGTTTTCATTCAAGCTGCTTCACTTAAGCAACTTAAGTT
TTCCGTTTTGGTAGAAAAAACTGTCCTCACACCACCAGCTGCTTTCTCCAACATTCTTTCACTGGGATAAGA
CAGGCGAGTGTCTGCCTTTCATGTAATTGAATCCCCTGGTTTTCAAAGGGAACTTTGCTGTACATGGAATGA
AGGTAACATTTGCATTCTGTGAAAAACACGCAGATGATTTGTCTTAAACTAGAGGAGGGAAGAAGCGGTTC
AGTTTTGCTGACTAAACCTTGTGTGGTTAACACCAGCAGGTAGGTATCTTATTCCTTGCAAGAAAGAGAAA
CCAAAAGACAGTTGCTCTCATTCTGGGGGAATGAAGTGATCGGGATCCACTTACTGTGCATGAAAGTCCCT
CTGTGTTACATACTCAGCAATGTTGCATGTACATTGTCTCCCTGAGTAAAGAGTTGTAATTTTCTCCCGTTCG
AGTACGTGAAGCTCTGTGGGGTTTACAGCTTTCTGGGAGGCATTCTGTGCTGTGTGGCCGAGAACGCCCTCT
TACAGATGGTATCCCTGAAGCAGAAGAGAAAGCGTGTTTCTCCCTTTCTGGTATAAGACAGTGGCAATGCT
CTAGTTTTGCTAACTAAACACTATGTTGGCAGCACATGCATGTTCCCA
>BMBL01022471.1_3
GTTGAAAACATGAAAGAAAGAGCAAAATACAGTGTTCTCATTCAAGCTGCTTCACTTTAGCAACTTAAGTT
TTCAGTTTTGGTGAACATACCGTCCTCACACCACCAGCTGCTTTCTCCACCTTTCTTTCAAAGGAATAAGAC
AGGCGAGTGTCTGCCTTTCAAGTACTTGAATACACTGGTTTTCAAAGGGAGCACTGCAGTATGGGGAATGA
AGGTAACGTTAGCATTCTATGAAAAAAAACGCAGATTATTTGCCTTCAGCTAGAGGAGGGCAGAAGTGGTT
CAGTTTTGCTGACTAAAGCTTGTGTGGTTAACGCGAGCAGGTATCCTATTCCTTGCAAGAAAGAGAAACCA
AAAGACAGTTGCTGTCATTCTGGGGGATGAATTGAAAAGGATTCACTTACTGTGTTTGAAAATCCCTCTATG
CTACATGTCCAAGAAAGTTGCCTGTGCATTGTATCCCTGGGTAAAGGCTTTGAATTATATCCAGTTCAAGTA
CGTGAAGTCCTGCGTGGTTTACAGCTTTCCGGGAGGCACTCTGTGCTGCGCGGGTGAGAAGGCCTCCTTAC
AGACCTTATGCCTGAAGAAGAAGAGAAAGCGTGTTCCTCCCATTCTGGTATAGCCCAGTGGCAATGCGGTT
TTGCCAAGTAACCACTATTTTAGCAGCACTTGCAAGTTCCCA
>BMBL01022471.1_4
ATTCAAAGCATGACAGATAAAGCAAAATAAGTGTTCTCATTCAAACTGCTTCACTTAAGCAACTTAAGTTTT
CAGTTTTGGTAGAAAAAACTATCCTCACACCACCAGCTGCTTTCTCCCATGTTCTTTCAATGGGATAAGACT
GGCGAGTGTCTGCCTTTCTAGTACTTGAATCCCTTGGTTTTCAAAAGGAACTTTGCAGTACAGGGAAGGAA
GGTAACATTTGCATTCTATGAAAAACATGCAGATTGTTTGCCTTCCGCTAGAGGAGGGCAGAAGTGGTTCA
GTTTTGCTGACTAAAGCTTGTGTGGTTAACACGAGCAGGTATCCTATTCCTTGCAAGAAAGAGAAACCAAA
AGACAGTTGCTCTAGTTCTGGGGGATGAAGTGAGAAGGTTTCACCTACTGTGCTTTAATATCCCCTCTGTGT
TACATGCCCAAGAAAGTTGCATGTGCATTGTCTTCCTGGGTTAAGAGTTTGAACTTTCTCCTGTTCGATTAC
GTCAAGCCCTGTGTGGTTTACAGCTTTCCTGTAACCACTGGTTGCTGCGCGGCCAAGAACGCCACCTTATAG
AAGGTATCCCTGAAGGAGAAGGGTAAGCGTGTTTTTCCCATTCTGGTAAAGACAGCGGCAATGCTGTAGTT
TTGCTAACTAAACAGTATTTTAGCAGCACGTGCAAGTTTCCA
>BMBL01022471.1_5
ATTGAAAGCATGAAAGATAAAGCAAAATACAGTGTTCTCATTCAAGCTGCTTCACTTAAGCAGTTTTGGTG
GAATATACCGTCCTCACACCACCAGCTGCTTTCTCCACCTTTCTTTCAACGGAATAAGACTGGCGAGTGTCT
GCCTTTCAAGTAATTGAATACCTTGGTTTTCAAAGGGAACTTTTCAGTACATGGAATGAAGGTAACATTTGC
CTTCTATGAAAAAAACGCAGATTGTTTGCCTTCCGCTAGAGGAGGGCAGAAGTGGTTCAGTTTTGCTGACT
AAAGCTTGTGTGGTTAACACGAGCAGATATCCTATTCCTTGCAAGAAAGAGAAACCGAAAGACAGTTGCTC

TCATTCTGGGGGATGAAGTGAGAAGGATGCACTTACTGTGCTTGAAAATCTCTCTGTGCTACATATCCAAG
AAAGTTGCCTGTGCATTGTATCCCTGGGTAAAGGCTTTGAATTATCTCCCGTTCAAATACTTGATGCCCTGC
GGGGTTTACAGCTTTCAGGGAGGCACTCTGTGCTGTGCGGCCGAGATCGCCGCCTTACAGACGGTATCCCT
GAAGCAGAAGAGAAAGCGTGTTTCTCCCGTTCTGGTATACAACAGCAGCAACGCTGTAGTTGTGCTAACTA
AACACTATGTTAGCAGCACGTGCAAGTTCCCA
>BMBL01022471.1_6
ATTCAATGAATGAAAAAGAAATCAAAATACAGTGTTCACATTCAAGCTGGTTAACTTAATCTACTTAATTTA
TTGTTTTGCATAAAACACACTGCTCACACCATTTATAAGCTTCGTCCAACATTCTCTCACTGGGATAAGACT
GGGAGTCAATTCCTATGAAGTATTTGAATCCCCAGCTTTAAAAGGGAACTTTGCGTTACAGGGAATGAAGG
TAGCTTTCACATTCCAGGAAGAGCAAGCAGATTCTTTCGCCTTCAGCTAATGGCTCTCAGAGGCAGTTCAGT
TTTGATGACTAAAGCTTGTGTTGTAACCGCGTGCATGTTTCTTATTCAGTGCAACAAAGAGAAAGCAAACG
ACAGTTGCTCTCAGTCTGGGTGATGCAGTTAGAAGGATTCACTGAGTTTGAAAATCCCTCTGTGTGACATGC
CCAACAATTCTGCCTGTGCGTTGTCTACCTGGGTAAAGAGTTTGACTTTACTCCCCTTCCAGTAAGTGAAGT
CCTTCGGGTTTACAGCTTACCAGGTGTCACTCAGTGCTGTGCGGCCCAGAATGCGGCCTTACGGACAGTAT
GTCTGAAGGAGAAAACAAAGCGCGTTTCTCCCGTTCTGGTCTATGACCGCTTTAGAGATGTAGTTTTGCTAA
GTAAACGCTACTTTAGCAGGACCTGCATGTTCCCA

Supplementary Sequence File 3. TMAsat monomeric sequences (in fasta) manually isolated from the *Hydrodamalis gigas* assembled reference genome (accession GCA_013391785.1).

>JACANZ010423367.1
GTTCACTGTATATAAGAGAGCAAAATACAGTGTTTTCATTCAAGCTGGTTAACTTAATCAACTGAAGTTTTC
AGTTTTGGTAGAACAAACAGTTCTGACACCACCAGCTACTTTCTGAAACATTTTCTCAATGGGATAAGTCTA
GCGAGTGTCTGCCTTTCAAGTACTTCAATCCCCTGGTTTTCAAAGGAAATTTTGCATTACAGGGAACGAAGG
AACTTTCGCATTCTAAGGGAAACAGGCAGATTGCCTTTAGCTAGAGGACGGCAGAAACAGTTCAGTTTTGG
ATACGAAATCTTGTGTGGTTAACATGAGTAAGTATCCTATTTCCTGCAACAAAGAGAAACCAAAAAGTTGC
TCTCTGTTTCAGGAATGAAGTGAGAAGGATTCATTTACTGTGTTTGAAAATCCCTTTATGTTACTTGCCCAA
GAAAGTGGCTGGTGCATTGTCTACCTGGGTAAAAAGTTTGACCTTTCTCCCATTCCGGTACTTCAAGCCCTG
CGTGGTTTACAGCTTTCCGGGAGGCATTCTGTGCTGCACGGCTGAGAATGCGGCCTTACGGACGGTATCCCT
GAAGGAGAAGACAAAGCGTGTTTCCCCCATTCTGGTATATGACAGTGGCAGGGCTGTAGTTTTGCTAAGTA
AACACTGTTAGCAGCAAGTACATGTTCCCA
>JACANZ010389660.1
ATTCACTGCATGAAAAAGAAAGCAAAATGCAGTGTTCTCATTCCAGCCAGTTTACCTAATCAACTGAAGTT
TTCAGTTTTGGTACAACAAACAGTCAGGACAGCACCAGCTACTTTGTGAAACATTCTCTCAATGGGATTAA
CTGTCAAGGGTCTGCCTTTCTAGTACTTGAATCACCTGGTGTTCAAATGGAAATCTGAATTACAGGGAATGA
AGGAAGCTTTCACATTCAAAGAAACAGGCAGATTGTTTGCCTTCAGCTAGAGGACAGCAGAAGCTGTTCAG
TTTTGCTGACTAAAGCTTGTGTGGTTAACATGAGCAGGTATCCTATTCCCTGCAAGAAAGAGAAACCAAAA
GATAGTTGCTCTCACTCCGGGGAATGAAGAGAGAAGGTTTCACTTATTGTGTTTGAAAATCCCTCCGTTTTA
TATGCCCAAGAAACTTGCCTGTGCATTGTCTACCTGGGTCAAGAGTTTGACTTTTCTCCCCTTAGAATACGT
GAAGCCCTGCAGAGTTTATAGCTTTCCGGAGGCACTCTGTGCTGCGCGGCAGAAAATGCTGCCTTACAGAC
GGTATCGCTGAAGGAGAATAGAAAGCTCGTTTCTCCGTTTCTGGTATAGGACAGCGGCAGCATTGTATTTTT
GCTAACTAAACATTTTGTTAGCAGCTCATGCATGTTCCAA
>JACANZ010311924.1
ATTCCCAGTATGAAAGGGAAGGCAAAATACAGTGTTCACATTCAAGCTGGTTAAGTTAAGCAACTTAAGTT
TTCAGNNTGGTAGAACAAAAGGTCCTCACAGTACCAGCTCATTTCTCAAACATTCTCTAAGTGGAATAAAA
CTGGCAAGTGTCTGCCTTTCAAGTACTTGAATCCTCTGCTTTTCAAAGGAACTTTGCATTACAGGGACTAAA
GGTAGCTTTTGCATTCTAAGCAAAACAGGCAGACTGTTTGCCTTCAGCTGGAGGATGTCAGCAGGGGTTCA
GTTCTGCTGGCTAATCCTTGTGTGTTTAACACAAGCATTTCTTATTCCTTGCAAGAAAGAGAAAGCAGAAGA
CAGATGCTCTCAATCTGGGGGATAAAGTGAGAAGAAGTTACTCACTGTGTTTGAAAATCCCTCTCTGTTACT
GGCCCAGGACAGCTGCCTGTGCATTGTATACCTGGATAAGAGTTTGAATTTTCTCCCATTGGAGTTCGTGAA
GCCCTGCGGGGTTTACAGCTTTTCAGGAGGCACTCTGTGCTGTGAGGCCAAGAATTCTGCCTTACAGAGGG
TATCCCTAAAGGAGAAGAGAGCACATTTCTCCCATCCTGGTATACAACAGTGGTAGCGCTGTAGTTTTGCT
GAGTAAAGATTATGTTAGCAGCAAATGCAAGTTCCCAC
>JACANZ010229584.1
ATTCACTGTATGAAAAAGAAAGCAAAATGCAGCGTTCTCATTCAAGCCTGTTTACTTAATGTAGTGAAGTTT
TCCGTTTTGGTAGAACAAGCAGTCCTGCACCACCAGCTACTCTCTGAAACATTGTCTGAATGGGATATAACT
GGTCTTTCAAGTACTTTGATCCTCTGGTTTTCAAAGGGAAGTTTGTATTACAGGGAATGAAGGTAACTTTCG

CATTCTAAAGAAAAGAGGCAGATTGTTTGCCTTTAGCTAGAGGACAGCAGCAGCAGTTCAGTTTTGCTGAC
TAAAGCTTGTGTGGTTAATATGAGCATGTTTCTTATTCCCTGCAAGAAAGAGAAACCAAAAGACTGTTGCTC
TCAGTCTGAGAAGGATTCACTTACCGTGTTTGATAATATCTCGGTGTTACATGCCCAAGAAAGTTTCCTGTG
CATTGTCTACCTGGGTAAAGAGCTTGACTTTTCTCTCCTTCTAGTACGTGAAGCCCTGCGGGGTTTACAGCT
TTCTAGGAGGCACTCTGAGCTGCACGGCGGAGAATGCCTCCTTACAGACCTTATCGCTGAAGGGAAGAAAA
AGCACATTTCTCCCCTTCTCGTATACGACAGCATCAGCGCTTTAGTTTTTCTAACTAAACACTATGTTAGCA
GCACGTGCATGTTCCCA
>JACANZ010159291.1_1
AGCCAACTACAGTCTTCTCATTCAAGCTGGTTAACTTAATCAACTTAAGTTTTCAAATGTGGTAGAACAAAG
AGTCCTCACACCACCAGCTGCTTTCTCAAACATTCTCTCAATGGGATAAGATTGGCGAGTGTCTGCCTTTCA
AGTACTTGAATCCCCTCGGTTTCAAAGGGAACTTTGCATTACATGGAATGAAGGTACATTTTGCATTCTAAG
AGAAACAGGCAGATTGTTTGCCTCCAGCTAGATGATGGCAGAAGCGGTTCAGTTTCCCTGAGTAAATCTTG
TCTTGTCAGCACATGAATGTTTCCTATTCACTGCAGAAATGAGAAACCAAAAGATAGTTGCTCTCAGTCCG
GTGCATGAACTGAGAAGGATTCACTTACTGTCTTTGGAAATCCCTCTGTGTTACTTGCCCAGGACTGTTCCG
TGTGCATTCTTTACCTGGGTAAACACTTTCACTTTTCTCCATTTCGACTACATGAGCTCTGCGGGTTTCCAAC
TCTCTGGGAGGCACTCTGTGCTGTGCAGCGAAGAATGCAGCCTTACAGCCGATATCGCTGAGGGAGAAAAC
AAAGCGCATTTCTCCCATTCTGGCATACGACAGCAGTAGAGATGTAGTTTTGTAAAGTAAACACTACGTTA
ACAGGATGTGCATGTTCCCA
>JACANZ010159291.1_2
ATTTACCGCATGGAAGAGACAGCAAAATAGAGTGTTCTCATTTAATCTGATTAACTTAATCAAGCTAAGCT
ATCAGTTTTGATAAAACACAGTCGTCACACTATCTACAAGCTTTCTCAAACATTCTGTCAGTGGAATAGTAC
TGGTGAGTCTCTGCCTTTCAAGTACCTGAATCCTGAGGTTTTCAAAGGGAAATTTTCATTACAGTTAATGAA
GTAACTTTTGCATTCTAGGAAGAACAAGCAGATTATTTTCCCTTCAGCTATAAGATGGCAGAAGTGGTTCAG
TTTTGCTGACTAAACTTTGTGTTGCAAGCACGTGCATGTTTCCTATTCACTGCAAGAAAGGGAAACCAAAAT
ACAGTTGCTCTCATTCTAGAGTACGAATTGAGAAGGATTCACTTACTGTGTTTGAAAATCCCTTTGTGTTAC
ATGCCCAAGAATGTTGCTTATGCAGTCTCCACCTGGGTAAAAGTTTGACTTTTCTCCCCTTCGAGCACATGA
ATGCCTGCCAAATTTACAGCTTTCCAGGAGGCTATCTGTTCTGAACGGCTGAGAATGCAGCCTTACAGATG
GTATCTGTGAAGCAGAAAACAAAACGTGTTTCTGCCTTTCAAGTATGCTGCAGCAACAGAAATGTAGTTTT
GCAAAGAAACACTATGTTACCACAACATACATGTTCCC
>JACANZ010122094.1_1
ATTCACTACACGAAACATAAACCAAAATACAGTATTCTCATTCAAGTTGGTTAACTTAATCTAGTTGATTAA
CTTATCTGTTTTGATAAAACACAGTTCCCACATAATTGAGAACCCTTCTTATACATTCTCTGAATGGCAATA
GAATATCAAGTTTTTCCCTTTGTAAACTGTGAAGTCCACGAGTTTTCAGATATTTTCTATGCGAATTTTGTGT
TACCAGAAATGAATGAGACTTTCACAATCCAGGCATAACAAGACAGACTCTTTCCCATTCAACTACAGGAC
CACAAAAACAATGTACTTTTGCTGACTAAACCTTGTGTTGTAAGCACGTGCGTGTTTCCAATCCACTGCACA
AAAGAGAAACCAAAATACAGTTTCTCATATTCTGGTTGATTTAAGTCAGAAGGATTAACTTATTGTGTTTAA
AAATCCCTCTGTGTTACATGCCCGAGAATGTTGCCTATACATTCTCCACCTGGATAAAGAATTTAACTTTTC
TCCCATTAGACTACATGAAGCCATGCGGAGTTTAGATGTTTTCAGTATGCATTCTGTGTTACCTAGCCAAAA
ATGTGCCTTACAGACCGAATCTTTGGAGGAGAAAACAATGTGAGCTTCTTCCTTTCTAGTACTTGAATCCTC
CAGGTTTTCAAATGTTTTCTATGGGAACTTTGCAATACCGGGAATGAATTGCCTT
>JACANZ010122094.1_2
ATTCACTGCATGAAAAATAAAGCAAAACACAGTATTCTCATACAATCCTGTTTACTTAATCATCTGAAGTTT
TCAGTATTGGTTAGAACAAGCAGTCTTAGGACCACCAGCTATTTTCTGAAACATTCTCTCAATAGGATAAA
GTACTTGAATCTGCTGGTTTTCAAATGGAAATTTGAATTATGGGGAATGATGGAATGTTTCATATTCAAAAA
AAAACAAAAAACAAAACAGGCAGATTGTCTTCTGCTAGAGGACTGAAGAAGTAGTTCAGTTTTGCTGACTA
AAGCTTGCATGGTTAACATGAGCATGTATGCTATTCCCTGCAAGGAAGAGAAACCAAAAGACTGTTGCCCT
CAGTCTGGGGAACGAGGTGAGAAGGATTCACTTATTGTTTTGAAAATCCCTCCCAAGAAAGTTGCCTGTGC
ATTGTCTACCTGGGTAAAGGGTTTGCTTTTTCTCCTGTTTTTGAAAGTGAAGCCTTGTGGGGTTTACAGCTTT
CTGTGAGGCATTCTGTGCTGTGCGGCGGAGAATGCTGCATGACAGACTGTATCCCTAAAGGAGAAGACAAA
CCGCATTTCTCCCATTCTGGTATATGATAGCGGCAGCGTTGTAGTTTTCCTGAGTAAAGACAATCTTGGCAG
CAAGTGTAAGTACCCAT
>JACANZ010120854.1_1
ATTCACCACATGAAAGAGAAAACACATTACAACGTTCGCCTTCCAGCTGCTTAACTGAATCTACTGAAGTT
TCCAGTTTTGTTAGAAGAAAGTGTCCGCCCAGCACAAGCTACTTTCTCCAATGTTATCAGAGTGGGAGAAG
GCTGGAGAGTGCGGGCCAAGCACGTATTTGAATCCCCTGATTTTCAAAGGGATCTTTGCGTACCAGGGAAT
GAAGGGATTACTGGCTTTCAAAGCCACACCTGCCCATTGTCTGCCTTCAGCTAGAGGATGGTAGAAGAGGG
TCAGGTTTCCTAAGTAAACCTTGTGTGCTTAACACGATCCTGTAGCTTCTTCCCTGCCTGAAAGACAAAGCA

AGAGCCTGTTGCTCTCAGTCCAGGGTTGAAGGGTGAACGACTCACTTTCTGTGTTTCAAAATCCCTCTGTGT
TACATGCCTAAGGAAGTTGCCTGTGCATTGTCTACCCGGATACAGTGTATGACGTTTCTCCCTTTCCAGCAG
TTGAAGCCATTCAGAGTTTAGAGCATTCTGGAAGGGATTCTGTGCTGCGTGGCTGAGAATACCGCTTTACA
GACTATTCTGAAAGGAGAAGAGAAACCGCGTACCTCCCGTTCTGGAACACGAACGAGGCAAAACTGTAGT
GGTGGAGAGTAAACACTTTGTTAGCAGCAAGTGTAAGTTCCCA
>JACANZ010120854.1_2
ATTCCCCACGTGAAAGAGAAAGCAAAATGCAGCGTTCTCCTTCAAGTTGCTCAACTGAAGCTACTGAAATT
TCCCATTTGGTTAGAAGAAAATGTCCGCCCACCAGAAGCTGGTTTCTCAAACGTTCTCTCAGTGGGCAAAG
GCTGGAGAGTGCCTGCCTTGGAAGTATTTGAATCCTCTGGTTTTCAACGGAAATTTGGAATAAGAGGGAAT
AGAGGTACTACTGGCTTTCAAAGCAAAACGCAGAGATTGTTTGACTTCACCTAGAAGATGAAAGAAGAGG
TTCAGTTTTACTGACTAAAACTTGAGTGCTTACGCGAGTGTGTAGACTCTTCCCTGCCTGAAAGAGAAAGCA
AGAGCTTGTTGCTGTCAGTCTGGGGATGAAGGGAGAACGATTCACTTTCTGTGTTTGAAAATCCCTCTGTGT
TCCATGCCTAAGAAAGTTGCCTGTGCATTGTCTACCTGGAAAAAGTGTATGACTTTTCTCCCTTTCAAGTAG
TTGAAGCCGCTCAGGGTTTCCAACATTTGGTAGGCATTGTTTGCTGCACGGCTGAGAATACAGCTTTACAGA
CGGTAGCGCTAAAGGAGAAGAGAAACTGCCTTTCTCCCATTCTGGAACACGAATGATACAAAACCGTAGTG
GTGCTGATTAAAAACTTTGTTAGCAGCAATTCTTAGTTCCCA
>JACANZ010078570.1_1
ATTCACCACATGAAAGAGAAAGCAAAATACACCGTTCTCCTTCAAGCTGCTTAACAGAATCTACGGAAGTT
TTCAGTTTTGTTAGAGGAAAGAGTCCGCCCAGGACAAGCTGCTTTCTCAAACGTTCCTTGAGCGGGAGAAG
GCTGGAGTGTGGAGGCCTAGCAAAGATTTGAATGCCCTGGTTTTCAAAGGGAACTTTGCATTCCAGGGAAT
GAAGGTACTACTGGATTTCAAAGCAAAACAGTCAGATTGCGTTCAGCAAGAGGACGGCAGACGAAGTTCA
GTTTTGCTGACTAAACCTTGTGTGCTTAACACACACATGTAGCTTTTTCCCTGCCTGAAAGAGAAAGCAAGA
GTTTGTTGCTCTCAGATTGGGGATGAAGGGAGAACGATTCCCGTTCTGTGTTTGAAAATCCCTCTGTGTTAC
ATGCCTAGGAAAGTTGCCTGTGCATTGTCTACCTGGATAAAGTGTATGACGTTTCTCCCTTTCAAGTAATTG
AAGCCTTTTAGGGTTTCCAGCATTCCGGGAGGCATTCTGTGCTGCTCGGCTGAGCATACCGCTTTACACACG
GTATCGCTAAAGGAGAAAAGAAACCGCGTTTCTCCCATTCTGGAACGTGAAGTAGGCAAAACTGTTGTTCT
GCTTAGTAAACGTTTGTTAGCAGCAAGGGCAAGATCCCC
>JACANZ010078570.1_2
ATTCACCTCATGAAAGAGAAAGCAAAATACAGTGTACTCTTTCTTGCTGCTTTTCTCAATCTACTGAAGTTT
TCATTTTTGTTAGAAGAAAGAGTCCGCCCAGCACAAGCTTCTTTCTCAAATGTTCTCTGAGTGGGAGAAGGC
TGGAGAGAGCAGGCCTAGCAAGGATTTGAATCCCGTGGTTTTCAAAGGGAACTTTGCATACTAGGGAATGA
ATATACTACCGGCCATTAAACCCAAGCAGGCAGATTGTCTACCTTCAGCTAGAGGATGGCCGAAGAGGTTC
AGTTTGGCTGACTAAACCTTGGGTGCTTAACAGGAGCATGTAGCTTCTTCCCTGCCTGAAAGAGAAAGCAA
GAGTTTGTTGCTCTCAGTCTGGGGATGAACGGAGCACGATTCACTTTCTGTGTTTGAAAATCCCTCTGTGTT
ACATGCCTAAGAAAGTTGCCTGTGCATTGTCTACCTGGATAAAGTGTATGATGTTTCTCCCTTTGAAGTAGT
TGAAGCCTTTCGGGGTTTCCAGCATTCCGCGAGGCATTCTGTGCTGCGCGGCTGAGAATCTCGCTTTACAGA
TGGTATCACTAAAGGAGAAGAGGAACCGTGTTTCTCCCATTCTGGAATTCGAAGGAGGAAAAACCGTAGTT
GTGCTGAGTAAAAAGTTTGTTAGCAGCAAGGGCAAGTTCCCC
>JACANZ010060488.1
AGTTCACTGTCTGAAAGCAAAAGCAAAATACAGCATTCTCCTTCAAGCTGCTTTACTTAATACACTGAAGTT
TTCAGTATTGGTAGAAGAAACAGTCCGCACACCACCAGCTGCTTTGGGAAATGTTCTCCCAGTGGGATAAG
ACTGGAGAGTGCCTGCCTTACAAGTACTTGAATCCCTTGGTTTTCAAAGGGAACTTTGCATAACAGGGAAT
GAATGTACCTTTGGCATTCAAAGCAAAACAGGCAGATTGTTTGCCTTCAGCTAGAGGATGGCAGAAGAGGT
TCAGTTTTGCTGACTAAACTGTGTGCTTAACACGAGCATGTAGTTTCTTCTGTGCCAGAAAGAGGAAGCAA
AAGCCAGTTGCTCTCAGTGTGGGGGATGGAGTGAGAAGGATTCACTTACTGTGTTTGAAATCTCTCCGTGTT
ACATACCTAATAAAGTTGCCGGGGCCTTGTCTACCTGGGTAGAGGGTTTGACTTTTCTCTTGTTTGAGTAGG
TGAGCCCTTGCAGGGTTTACAGCTTTCTGGGAACCATTCTGTGCTGGGCAGCTGAGAATGCCGCCTTACAG
ACGGTATCCCTGAAGGAGGAGATAAAGAGGGTTTCCCCATTCTGGTATACAACAGAGGCAGCGCTGTACTT
TGGCTAACTTCTATTTTACCAGCATGTTCATGTTCCCA
>JACANZ010031279.1
ATTCACCACCTGGAAGAAAAAGCAAAACACACTGTTTTCCTTCAAGCTGCTTAACTGAATCTACTGAAGGT
TTCAGTTTTCTTAGAAGAAACAGTCCGCCCACCAGAAGCTGCATTTACAAATGTTCACTCTGTGGGAGAAG
AGTGGAGAGTGTCTCCCTTGGGAGAATTTGAATCCCCTGGTTTTCAAAGGGAAATTTGCATCACAGAGTAG
GAAGGTACATTTGGCTTTCGATGCAAAACAGGCAGATTGTTTGCCTTCAGCTAGAGGATGGCAGAAGAGGT
TCAGTTTTGCTGACTAAACCTTGTGTGCTTAAGGCGAGCGTTAACTCCTTCCCTGCCAGCTAGAGAAAGCAA
GAGACTGTTGCTCTCAGTCTGGGGATCGAGAACGATTCACTTACTGTGTTTGAAAATCCCTCTATGTTCCAT
GCCTAAGAAAGTTGCTTGTGTATTGTCTACCTGGAAAAAGTGTATGACTATTATCCGTTTTAAGTAGTTGAA
GCCATTCTGGGTTTACAGCATTCCGGGAGGGATTCTGTGCTGTGCGGTTGAGAATATCTCTCTACAGATGGT

ATCCCTAAAGGAGAAGAGAAACGGCATTTCTCCCCTTCTGAAACACGACTGAGGCAGAGCCGCTGTTTTGC
CGATTAAAAACTACGTTAGCAACAAGGAGACGTTCCCT
>JACANZ010507883.1
ATTCACCGTATGAAATAGAAAGCAAAATACAGCGTTCCCTTCAAGCTTCTTTACTGAATCTACTGAAGAAA
GAGTTCGCCCACCACCAGCTCTTTGCTCAAATGTTCTCTCTCAGAGGGAGAAGACTGGAGAGTGTCTGCCTT
GGAAGCATTTGAATCTCCTGATTTTCAAAGGGAACTTTGCATAACAGGGAATGAGTTACTTCTGGCATTCAA
AGCAAAACAGGCAGATTGTTGGCCTTCAGCTAGAGGAAATGCAGAAGACGTTCAGTGTTGCTGACTAAACC
TTATTTGCTTAATGTGAGCATGTAGCTTATACCTGCCAGAAAGAGAAAGCAAAGCAAGTTGCTGTGAGTCT
GGAGGATGAAGTGAGAAGGAGTCACCTACTGTGTTTGAAAATTCCTCTGCGTTACATGTCTAAGAAAGTTG
CCTCTACATTGTCTCCCTCGGTAAAGGGATCCACGTTTCTCCCTTTCAAGTATGTGAAGCCATTCAGAGTTT
ACAGCTTTCGAGGAGGCATTCTGTCCTGCGCGGCCAAGAATGCCGCCTTTCAGACGGTATCACAGAAGGGA
GTGAAACCGTGTTTCTCCCGTTATGGTATAGGACAGCGGTGGAGCCACAGTTTTGCTGAGTAAAGACTATG
GTACCAGCAAGTGTAAGTTCCCA
>JACANZ010255388.1
ATTCAGTGCATGAAAAAGAAAGCAATATGCAGTGTTCTCTTTCAAGCTGCTTCATTTAAACAACTTAAGTTT
TCAGTGTTGGTAGAACAAAGCGTCCTCATACCACCGGCTGCTTTCTCCACCTTTCTTGCAATGGTGGGAGAA
GGCTGGAGAGTGCAGGCCTAGCAAGTATTTGCATCCCCTGGTTTTCAAAGGGAACTTTGCAGTACATGGAA
TGAAGGTAAGATTTGCATTCTGTGAAAAACATGCAGGTTTTTTGCCTTCAGCTAGAGAAGGGTGGAAGCGG
ATCAGTTTTGCTGACTATATCCTGTGTGGTTAACACGAGCAGGTATCTTATTCCTTCTAAGACAGAGAAACC
AAAAGACAGTTGCTCTCATTCTGGGGGATGAAGTGAGATGGTTTCACTTACTGTGCTTGAATATCCCTCTAT
GTTACATGCCCAGGAAATTTTTCTGTCGATTTTCTCCCTGAGTAAAGTGTTTGAATTTTCTCCCCTTCGAGTA
CAAGAAGCCCTGGGGGTTCACAGCTTTCCCGAGCCACTAGTTGCTGCGTGGCCTAGAACACCGCCTTATAG
ACCGTATGCCTGAAGGAGAAGAGAAAGCGTGTTTTTCCTGTTCTGGTATAAGACAGCGGCAATGTTGTAGT
TTTGCTAACTAAAGACTATATTAGCAGCACTTGCAAGTTCCCAN
>JACANZ010245123.1
ATTCACTGCAAGAAAGAGAAAGCAAAATACAGTGTTCTCTTTCAAGCTGGTTAACTTAATCAGTTAAGTTTT
CAGTTTTGATAGAACAAACAGTCCTCACACCACGTACTAGATTTCTCAAACTTTCTCTCAATGGGATAAGAC
TGGCGAGTTTCTGTTTTTTATCTACTTGAATACCCTGGTTTTCAAAGGGAACTTTACATTACAGGGAATGAA
GTTACCTTTTGCATTCTAGGAAAAAGAGGGAGATTATTTCGCCTTCAGCTAGAGGACCGCAGAAGTGGTTC
AGTTTTGCTGATTAAACCTTATGTGGTTAGCACGTGTATGTTTCCTACTCCCTACAATAATGAGAAAGCAAA
AGACAGTTGCTCTTAGTCTGGGGGATGAACTAAGAAGGATTCCCTTAGTGTGCTTGAAGATCCCCCTGTGTT
ACATAACCAAGAAACTTGCCTGTGCATTGTCTATCTGGGTAAAGACTTTGACTTTTCTCCCGTTGGGTATGT
GAAGCCCTGTGGGCTTACAGCTTTTTGGGAGGCACTCTGTGCTGTGCGGCCAAGAATGCTGCCTTACAGAC
CTATCTCCATAGTAGAGAAAGTGCATTTCTCCCCTTCTGCCATATGATAGTGGCAGTGCTGTAGTTTTGCTA
ACTAAACACTTGTTAGCAGCACATACATTTTCCCAG
>JACANZ010129270.1_1
ATTCAATGCTTGAATGAGAGAGAAAAATACAGTGTTGTCCTTCAAGCTGCTTAACTTAAGCTCTCACTTTTG
ATCAAACACGGTCCTCACACCATCTACAAACTTTCTCAAGGGTTCTCTCAATGGGAAAAGGCAGGCGAGCG
TCTCCCTTTCAAGTGCCCGAATCCCTTGGGTTGCAAATGGAACTTTGCTTTACAGGGAATGAAGGTACCTTT
CACACTCTAAGAAGAACAAGCAGACTGTTTCGGCTTCAGCTAGAGGAGAGCAGAAGCGGTTCATTTTTGCT
GGATAAACCTTGTGTGGTAAACGCGTTCATGTTTCCTATTCACTGCAAGAAAGGGAAACCAAAAGACCCTT
GCTCTCCGTCTGGTGGATGAAGTGAGAAGGATTCACTTACTGTGGTTGAAAACCCCTCTGGGTTATATGCAC
AAGAATGTTGCCTGTGCATTATCCATCTTTGTAAAGAGTTCCCCTTGTCTCTGCTGCGAGTACGTGAAACCC
TGCGGGTTTAACGGCTTTCCAGGAGGCACTCTGTGCTGCGCAGCTGAGAATGCGGCCTTACAGATGGTATG
TCTGAAGTCAGAAAGAAAGCGCTTTCCTCCCGTTCTAAGACAGCAGTAGGGATGTAGTTTTGCTAAGCAAA
CACTATGTGCACCTTCCCA
>JACANZ010129270.1_2
ATTCACTGCATGAAAGAGAGAGCAAACTGCACTGTTCTCATTCATGCTAGTTAACTTTCATCAGTTTTGATA
AAATACAGTTCTCGCTCCATCTACAAGCTTTCTCAAGCATTCTCTCAGTTGGATTAGAACAGCGAGTGTCTC
CTTTTGAAGTACGTGAATCCCCTGGCTTTCCAAGTGAACTTTACCTTCTAGAGAATGAAAGTACCTTTCGCA
CTCTAGGAAGAACAAGCAGATTGTTTGCCTTCAGCTAGAAGACAGCAGAAGCGGTTCAGTTTTACTGAGTA
AACTTGTGTGGTAACCGCGCACGTATTTCCTATTCACTGCAAGACAGACAAACCAAAAGAAACTTGTTCTC
CGTCTAGTGGATGAAGTAGGAAGGATTCACTTACTGTGATTGAAAATCCCTCTGTGTTACATGCCCAAGAA
TGTTGCCTGTGCATTGTCTACCTGAGTAAAGGGTTTGCCTTTTCTCCCTTCAGAGTACGTGAAGCCTGTGGG
GTTTACAGCTTTCAGGGAGACAGTTTGTGCTGCGTAGTCAAGAGGGCGGCCTGAGAGCTGGCATGTCTGAA
GGAGGAAAGAAAGCACGTTTCTCCCGTTCTGGTATAGGTGGGTGGTAGAGATGAAGTTGTGCTAAGTAAAC
ACTATGTTAGCAGGACTTGCAAGTTCCCA

>JACANZ010754534.1
ATTCACTGCATGAAATGAGAGCAAAATACAGTGTTCTTATTCAAGCTGGTTAAGTTCTCAGTTTTGATAAAA
CTTGGTCCTCACAGCATCTATAAGCTTCCTCAAGCATTCTCTCAATGGGACAAGACTGGTGAGTGTTTCCCT
TTCAAGTTCTTGAATTCCCTGGTTTTCAAAGGGAACTTTACATTATAGGGAATGACAGTACCTTTCCCATTCT
AGGAAGAACAAGCCAATTGTTTCGCCTTCAGCTAGAGGATTGCAGAAGTGGTTTAGTTTTGCTGAGTGAAC
CTTGTGTGTTAATGGCCTGCGTGTTTCCTATTCTCAGCAAGAAGGAGAAACCAAAAGACAGTTGCTCTCCGT
CTGCTGGATGAAGTGAGAAGGATTCATTTACTGTGGTTGAAAATCCCTCTGTGTTACATGCCCAAGAATGTT
TCCTGTGCATTGTCTACCTGGGTGAAGAGTTTGACTTTTCTCCACTTCCAGTACATGAGGCCCTGCAGGCTT
TCCAGCTTTCCAAGAGGCACTGTCTGCTGCACGGTTGAAAATGCGGCCTTATAGACGGTGTGTCTGAAGGA
AGAAAGAAAGCATGTTCCTCCAGTTCTGGTATACAATGGCAGTAGAGATGTATTTTTGCTAAGGAAACACT
ATGTTAGCAGGA
>JACANZ010175637.1
ACTCACTGCACAGAAGAGAAGGCAAAGTAGAGTGTTCTCATTCAATCTGGTTATCTTAATCAACTTGATTA
AGATATCAATTTTGATAAAACAGAGTCCTCACACCAGCTACAAGCTTTCTCAAACATTCACTTGGTGGAAT
AAGACTGGAGACTCCCTCCCTTTGAAGTACTAGAATCCTACGGTTTTCAAAGGGAACTTTGCATTACAGGT
ATGGAAGTTACCTCTTGCATTGTAGGAAGAACAAGGCACATTGTTTTGCCATCAGCTAAAGGACGGCAGAA
CCGGTTCAGTTTTGCTGATTAAACCTTGGGTTGTAACCACCTGCATGTTTGCTATTCACTGCAAGAAAGAGA
ACCCAAAAGACAGTTGCTCTAAGTCTGGTGAATGAAGTGGGTAGGATTTACTTACTGTGTTTGAAAATTCCT
CTGTGTTACATGCCCATATACATTCTACACCTGGGTAAGGAGTTTGACTTTTCTCCCCTTCCAGTACGTGAA
GCCCTGCGGGGTTTACAGCTTTGCGGGAGGCACTCTAGGCTGTTCGGCTGAGAATGCCACCTTACAGACGG
TATGTCTGAAGGAGAAAAAAAGGCACATTTCTCCCATTCTGGTATACGACAGCAGTAGAGACTTAGTTTTG
CCAAGGAAACACTATGTTAGCAGGACCTGCATGTTCCCA
>JACANZ010271070.1
ATTCACTGCATGAAAGAGAAATCAGAATACAATCTTCTCATTCAAGTTGATTAACTTAATCATGTTGATTAT
GTTGTCAATCGTGACACAGCACAGTCCTCACACCATCCACAAGCTTTCTCAAATATTATCTCCATGGGATAA
GAAAGGAGAGTCTCTCCTTTTCAAGTACTTTTATCCCCTGAGTTTTAAGATGTTTTCTAAGGGATCTTTGTGT
TCCTGGAATGAAGGTATCTTTCACAGTCTAGGAAGAACAAGGCAGATTGTCTCGCCTTCAGCTGAAAGACG
GCAGAAGCTCTGCAGTTTTGCTGACTGAAACTTGTGTTGTAATCACATGCATTTTTCTATCCCCTGCAAGAA
AGAGAAACCAAAAGACAGTTGCTCTCAGTCTGGGGAATGAAGTGAGAAGAATTCCCTTACTGTGTTTGAAA
TTCCCTCTGAGTGAAATGCCCTCGAACGTTGCCTGGGCATTTTCGCCCTGGCTAAAGATTTTGAGTTTTCTCC
CGTTTGAGTAGGTGAAGCCCTGCGGGGTTTACAGCTTTCTGGGAGGCACTCTGTGCTTCGCAACGGACAAG
GCCAGCTTACAGACGGTATCGCTGAAACTGAGGAGAAAGCCCATTTCCCTCTTTATGGTATAGGACAGCAG
CAGTGCTGTACTTTTGCTAAGGAAACACTATGTTAGCAGCACCTTCGTGTTCCCA
>JACANZ010035624.1_1
ATTCAGTGCAGAAAAGAGAAAACAAAATACGGTGTTCCTTTGAAGAAGGTTAACTTAAGTTGATTAGGTCA
TTAGTATTGATAAAACACAGTAGTCACACCATCCACAAGCTTTGTCAAACATTCTCTCAGTGGGATAAGAA
TGGAAAGTCTCTCCCTTTCAAGTACTTGAATTCCCCGGATGTTCAGAGGTTTTCTAGGAGAACTTTGCATTA
CCCGGTATGAAAGTACCTTTTTCTCTCCAGGAAGAACAAGGCAGATTGCTTAGCATTCATGTGAAGGATGG
AAGAAGTGGTGCAGTTTTGCTGACTAAGCCTTGTGTTGTAAGCACGTGCATGTTTCCAGTTCACTGCAAGAA
ACAGAAACCAAAAGGCAGTTGCTCTCAGTCTGGTGGATGAAATCAGAAGGATTCACTAACTGTTTGGAAAT
CCCTGTGTTACATGCCCAAGAAAGTAGCCATGCTTTCTCCATGTGGGTAAAGAGTTTGACTTTTCTCCCCTT
TGAGCAAGTGAAGCCCTGCGAGGTTTAGAGGTTTCCGAGAGCCACTGTGTGCTGCCTGGCTGAGAATGTGG
CCTTCTGGAAGGTATCTCTGAAGGAGAAACAATGCACGTTTCTCCCATTCAGGTATACGACAGCAGTAGAG
ATGTGCTTCTGCTAAGTAAACACTGTATTAGCAGAACGTGCATGTTCCCAG
>JACANZ010035624.1_2
ATTCCCTGAATGAAAGAGAAAGCAAAACACAATGTTCTCATTCAAGTTGGTTAACTTAATCAAGTTGAATA
AGTTATGAGTTTTGATAAACCACAGTCCTCAAACCATCCAAAAGCTTTCTCAGACACTCTCTCAGAGGGAT
AAGAATGGCGAGTCTCTCCCTTTCTAGTACTTGAATCCCCCAGGTTTTCTGTTGTTTTCTAAGAGAACTTTGC
ATTACCCAGAATGAGGGTAACTTTTGCATTCTAGGACGAACAAGGCAGATTGCTTTGCAATCAAGAGACGT
ACAGCAGAAGCAGTACGGTTTTTTTTTTTTTTAACTAACCCTTGTGTTGTAAACACTTGCATGTTTCCAGTTC
ACTGCAAGAAAGAGAAACCAAAACAGAGTTGCTCTCAGTCTGATGGATGAAGTCAGAAGGATTCACTGAC
TGTGTTTGGAAATCCCTCTGTGTTACATGCCCAATAATGTTGCCCATACCTTCTCCACCTGAATAAAGACTT
TGACTTTTTTCCCCTTCGAGTACGTGAAGCTCTGCAGGGTTTAGAAGTTTCCAGGAGGCACTCTGTGCTGCC
CGGCCGACAATGCGGCTTTACATATGGTATCTCTGAGGAAGAAAACAATGCACTTTTCTCCCGTTCAAGTAT
ACAACAGTAGAGATGTGATTTTGCTAAGTAAAGACTATGTTAGCAAGATATGCGTATTCCCA
>JACANZ010035624.1_3
ATTCACTGTATTAAAGAGAAAGCAAAACACAGTGTTCTCATTCAGGACGGTTAACTTAATCAAATCGAATA
AGTTATCAGTTTTGATAAACCACAGTCCTCACACCATCCACAAGCTTTCTAAAACATTTTCTCAGTGGGATA

AGAATGGCGAGTCTCTCCCTTTCAAGTCCTTGAATCCTGCGGGTTTTCTGTTGTTTTCTAAAAGAACTTTGCG
TTACCCAGAATGGAGGACCTTTTGCATTCTAGGAAGAACAAGGCAGATTGTTTGCCACTCAGCTAAAGGAC
GGCAGAAGCTGTGCAGTTTGGTGACTAACCCTTGAGTTGTAAGCACATGCATGTTTCCAATTAACTGAAAG
AAAGGGAAACCAAAAGGCAGTTGCTCTCAGTCTGCTGGATGAAGTGAGGATGCACTTAACGTGTTTGAAA
ATCCCTCTGTGTTACATGCTCAAGAACGTTGCCTGTGCATTTTTGCCCTGGGTAAAGTGTTTGACTTTTCTCC
CATTCGAATACGTGTATCCCTGCGGAGTTTACCGCTTTCCGTGTGGCTCTCTTTGCTGCGCTGCAGATAATG
CCGCCTTGCATACCTTATCGCTGAAGGGAAGAAAAAGCGCATTTCTCCCATTCTCATATACGACGGCAGCA
GTGCTGTACTTTTGCTAACTAAACACTATGTTAGCAACATTTGCATGCTCCCA
>JACANZ010035624.1_4
ATTCATTCACATGGGCAAGAAAAAGACCGTGTTCTCATTCAACCCTGTATACTTAATCAACTGAGGTTTTCC
GTTTTGGTAGAGCAAGCAGCCCTGACACCACCAGCTACTTTCTGAAACATTCTCTCAAGGGGTTAAGATTG
ACCAGTTTCTGCCTTTGAAGTACTTGAGTCCCCTGGTTTTCAAATGGAACTTTGCCTTACAAGCAAAAATGA
TAACTTTCACATTGTAAGGAAAATAGACAGATTGTTTACCTTCAGCTAGAGGATAGGAGAAGCAGTTCAGG
TTTGCTAACTAATGCTTGTGTGGTTAACATGAGCGTGTTTCCTATTCCCTGCAAGTAACAGAAACCTAAAGG
CTCTTGCTGTCAGTCTGGGTAATGAAGTGAGATGGAGTCACTTATTGTGTTTGAAAATCCCTCTCTGTTACA
GGCCCAAAAATGTAGCATGTACATTGTCTATCTGTGTAAAGAGTTTGCCTTTTCTCCCCTTCGAGTACCTGA
AGCCCTGCGGGGTTTACAGCTTTCCGGGAGGCCCTCCGTTTCTCCAGTTCTGGTATACGAGAGCGGCAGAG
CTGTAGTTTTGCTAACTAATCACTGTGGAAGAAGCACGTGCATGTTCCCAT
>JACANZ010107986.1_1
ACTCACTGCAAGAAAGAGAAAGCAAAATACAGTGTTCCCATTCAAGTTGGTTAACATAATCAAGTAGATTA
ACTTATGAGGTTTGATAAAACACACTCCTCACACCATCCACAAGCTTTCTCAAACATTCATTCAGTGGGATA
AGAATGGTGAATGTTGCCCTTTCAAGTACTTGAATCCGCCAGGTTTTCAGATGTTCTGTAACGGAGCTCTGT
GTTAGCCTGAATGCAGGTACCTTCCGCATTCCAGAAAGGACAAGGCAGATTGTTTCCAGTTCAGCTAAGGG
ACGATAAAAGCAGTGCAGGTTTGCTGAGTAAACCTTCTGTTGTGAGGACTTGCATGTTTCCAATACACTGA
AAGAAAAAGAAATCAAAAGATGGCTGCTCTCAGTCTGGTGGATGAAGTCAGAAGGATTCACTTACTGTGTT
TGAAAATCTCTCTCTGTTCCATGCCCAAGAATGTTGCCTGTGCATTCTCCACCTGGGTAAAGAGCTCGACTT
TTCTCCCCTTTCAGTACGTGAAGCCCTGCAGGGTTTAAGGCTTTCTGGTGGGCACTCTATGCTGCACAGCCA
AGAAACCAGCCTTACACAAAGTGTCTCTGAAGGGGAAAATAAAGCGCATTTCTCCCATTCTGGTATACCGC
AGCAGTAGAGATGAAGTTTTGCTAAATAAACACTATGTTAGCGGGACGTGCATGTTCTCA
>JACANZ010107986.1_2
ATCTCCTGCAGGAAAGAGAAAGCAAAATACAATGTCCTCATTCAAGTTGGCTAAGTTCAGTTCATCAAGTT
ATCAGTTTTGATAAAACACAGTCCTCACACCATCTACAAGCTCTTTAAGACATTCTCTCAATGGGATAAGAT
GAGTGAGTGTTTCCCTTTCAAGAACTTGAATCTCCTGGTTTTCAAAGGAAGTTTGCATTACAGGGAAGGAA
GGGGCCTTTGGCATTGTAGAAAATCAAGGCAGATTGTTTCAGCTTCATCTGAAGCATGGCAGAAGCAGTTC
AGTTTTGCTGAATAAATCTCGTGTTGTAAGCACGTGCATGTCCGTTATTCACTGAAAGAGAAACTAAAAGTC
AATTTTGTCACTCTTCTGGATGAAATGAGAATGAGTCACTTAACCGGTTTGAAAATCTGTCTGTGTTACATG
CCCAAGAATGTTGCCTATGAATTGTCCATCTGGATAAAAGCTTGGATATTTCTCCCTTTTAAGTACTTGATTT
AAGTACTGCGGGATTTACAGCTTTCCAGGAGGCATTCTATGCTGCCTGGCGAGAATGCAGCCTTACAGACA
GTATATCTGAGGGGAAAAAGAAACCACGTTTTCTTCCATTCTGGTATACCACAGCGGTACAGATGGAGTGT
TGCTAAGTAAACCCTATGTTAGGCAGACGTGCATGTTCCCT
>JACANZ010061387.1_1
AATCACTGTAAGAATGAGAAAGCAATATAGAGTGTTCTCCTTCAAGCTGGTTAACATACGTTCATCAGATT
CTCAGTTTTGATCAAACACAGTCCTCACATCACCTACAAACTATCTCAAACATTGTCTCACTGAAATAAGAT
TGGTGTCTCCCTTTTAAGTACTTCAATCTCCCAGTTCTGAAAAGGAACTTTGCAGTACAGTGAATGAAGGTA
CGTTTTGTATTCTAGGAAGAACGCAAACTGTCTCGCCTTCCGTTTAAGCACGGCAGAAGCGGTTCAGTTCTG
CTGACAAAACATTGTGTTGTTACCATGTGCATGTTTTCTCTTCACTGCAAAAAAGGGAAACCAAAAGACAG
TTGCTCTCACTCTGGGGGATGAGGTCAGAAGGACTCACTTAAGGAGTCTGAAAATCTCTCTGTGTAACATG
CCAAAGAAGGTTGCCTATGCATTCTCCACCTGGGTAAAGTGTTTCAGTTTTGTCCCTTTGGCGTATGTGAAG
CCCTGCAGGGTTTACAGCTTTCTGCCAGGCAGTCTGTACTGCCTGTGGAGAACGCAGCTGTACAAACGGTA
TCTCTGAAGGAGAAAAGAAAGCGCCTTTCCCCTGTTCTGGTACAGCACACCAACAGAGTTGTAGTTTTGCT
AAGTGAACACTATCCTAACATTACCTGCAAGTTCCCCT
>JACANZ010061387.1_2
ATTCACCGTATGAATGAGAAAGCAATATACAATGTTCTCCTTCAAGCTGGTTAAGTTCATCAAGTTCACCGT
TTTGAACAAACACAGTCCTCACACCATCTACAAACTTTCTGAAACATTCTCTCACAGAGATAAGAGTGGCA
AACGTCTGCCTTTCAAGTACTTCAAGTTCCCAGTTCTGAAAAGGAACTTTTCAATACAGTGAATGAAGGTAC
GTTTTGTGTTCTAGGAAGAACAATGCAAACTGTCTCGCCTTAGCTAAAGCACGCTAGAAGCAGTTCAGTTA
TGCTGACAGAACATTGTGTTGTTAACATGTGCATGTTTTATCTTCACTGCAAGAAAGAGAAACCGAAAGAC

AGGGGCTGTCACTCGGGGGGATGAAGTAAGAAGGACTCACTTAAGGTGTCTGAAAATCCCACTGTGTTACA
TGCCAAAGAAGGTTGCCTATGCATTCTCCACCTGGGGAAAATGTTTCAGTGTTCTCCCTTGGCCTTACCCGA
AGCCCTGTGGGGTTTACACCTCTCCGTGAGGCAGTCCGTGCTGCCAGGGGAGAATGTGGCCTTACAGATGG
TATCTCTGAAAGTGAGAAGAAAGCGCCTTTCCCCTGTTCTGGTAAAGCACAGCAACAGAGTTGTTTTGCTA
AGCCAACACTATGCTAACACTACCTGCAAGTTCCCCC
>JACANZ010515044.1
ATCCCCTGCAAGGAATAGAAAGCAAAACAATGATCTCATTCAAGCTGGTTAACGTAATCGACTTAAGATAT
GAGTTTTGATAACACACAGTCCTCACACTGTCTAGAAGCTTCCTCCAACATTCTCTCAAAGGGAGAAGGCT
GGGGAGTGTCTCCCTTTCAAGGACTTGAATGCCCTTTCTTTCCAAGTGAACTTTGCATTACAGGGAAACAGG
GTACCTTTTGCCTACTTGGAAGAAAAAGTCAGATTTTTCTGCCTTCAGCTAAAGCACGGCAGAAGGTGTTCA
GTTTTGATGACTAAACCTTGTGTTGTAAGCACGTGTATGTTTCCTGTTAAGTGCAAGAAAGAGAAACTAAA
AGACAGTTGCTCTCAGTCTGGTGGATAAGGTCAGAAAGATTCACTTACTGTGTTTCAAAATCTGTGTTACAT
GCTCAGGAATGCTGCTTGTGCATGCTCTCCCTGGAGAAAGATTTTGGGTTTTCTCCCCCTGGACTTCTTGAA
TCCCTGTGGCTTTTGCACCTTTCTGGGAGGCGCTCTTCGCTGCATGGCCAAGAAAGCAGATTTTTCAGGCGG
CATCTCTGAAGGAGAAAACAAAGTGCTTTTCTCCCGTTCTGGTATACGACAGCAGTAGAGATATAGTATCG
CTAAGCAAACTCTATGTTAGCAGGACATGTATGTTCCCAC
>JACANZ010190329.1_1
ACTTACTGTATGAAAGAGAAACCAGAATACAGGGTTCTCATTCAAATTGGTTAAGCTAATCAAGTTTATTA
AGTTATCAGTCTTTATACAACACTGTACTCACACCATCCACAAGCTTTCCAAACATTATCTCAATGGGATAA
GAAAGGAGAGTCTTTCCATTTCAAATGCTTGAATCCCCCGGATTTTCAGACGTCTTCTAAGGGATCTTTGTG
TTACCAAGAATGAAGGTTACCTTTCCCAGTCTAGGAAGAGCAAGGCAGATTGTCCCATCTCAAGCTAAAGG
ACTGCAGAAGCGGTGCAGTTTTGAGGAGTGAACCTTCTGTTGTAAGCACAGGCATGTTTCCTATTCACTGTA
AGAAAGAGATACCAAAAGACAGTTCATGCCAATCTGGTGGATGTAGTCTGAAGGATTCAGTTACTGTGTTT
CAAAATCCCTCTGTGTTACATGCCCCAGAATGTTGCCTATGCATTGTCCACCTAAGTAAAGTGTTTGACTTT
TCTCCTCTTCGAGTACATGAAGCCCTACAGCATTTACAGCTTTCGCGAGCACTCTGTGCTGCACTGCTGAGA
ATGCGGTCTTACAGGCAGTATCTCTGAAGGAGAAAAGAAAGTGCCTTTCTCCCGTTCTGCTGTAGGACAGC
AGTAGAGACGTAGTTTTGCTAAGTAAACACTATGTTAGCATTCCATGCATGTTCCCA
>JACANZ010190329.1_2
ATTCACTGTATCAATGAGAAAGCAATATACAGTGTTCTCATTCAAGCTGGTTAACTTAATCAAGTACATTAA
GTTCTCAGTTTTGATAAAACACACTCCTAACACCATCTATAGGCTTTCTCAAACATTCTCTCAATGGGATAA
GAGTGGCAAGTGTCTGCCTTTCAATTACTTTAATTCCCTGGTTTTCAAAGGGAGCTTTGCATTACAATGAAT
GAAGGTAACTTTCGCATTCTAGGAGGAACTGATTGTTTAACCTTCAGATATTCTGTTTTTCTGACTAAACCTT
CTGTTGTAACCACGTGCATGTTTCCTATTCACTGCAACAGAGAGAAACTAAACAACAGTTGCTGTCAGTCTG
GTTGATGAACTGAGAAGGATTCACTTAATATGTTCGATTGTCCCTCTGTCTTACATGTCCAAGAATGTTGCC
TGTGAATTCTCTTCCAGGGTAAAGCATTTCACTTTTCTCTCCTTAGAGTACGTGAAGCCTTACGGAGTTTAC
AGCTTTCCCTGGGGAACTCTGTGCTGGGCGCTCAAGAATGTGGCCTTAGAGATCTTATTCCTTAAGGAGAA
AACAAAGCCCATTTCTCCCGTTCTGATACATGATGGCAATAGAGACATAGTTTTGCTAAGGAAACACTACG
GTAGCAGAGCCTGCATGTTTCCTA
>JACANZ010402190.1
ATTCACTGCATGAAAGAGAAATCAAACTACAGTTTTCTCCTTCAAGCTGGTTAACTTAATACAGTTGATTAA
GTTATAAGTTTTCATCAAACACAGTTCTCACACGATCCACAACACTTCTCATACATTCTCTGAATGGGAGAA
GAATGTCAAGCTTCTCCCTTTCAAATATTGAATCCCCGTGGTGTTCAGATGTTTTCTGCGGGAGCTTTGCGTT
ACCAGGAATGAATGTCCTTTCACATTCTAGGAAGAACAAGGCAGACTGTTTCCTGTTCACGTAAAGGACAG
CAATAGTGATGTAGTTTAGCTGATTAAACCCTGTGTTGCAAGCACGTGTATGTTTCCAATTCACTGCAAGAA
AGAGAAACAAAAGTACTGTTTCTCTCATTAGGGTGGATGAAGTCAGAAGTATTAACTTACTGTGTTTGAAA
ATCTCTCTGTGTTACATGCACAAAAATGTTGCCTATACATGCTCCACCTTGGTAAAGAGTTGAACTTTTCTC
CCATTCGAGTACATGAAGCCAAGTAGGGTTTAGATGTTTTCACTAGGCACTCTGTGCTGCCCGGCTGAGAA
TGTGCCTTACAGACAGTATCTCTAAAGCAGAAAACAATGTGAGTTTCTCCCATTCAGGTGTAGGACGGCGA
TAGTGATGTCGTTTTGCTAAGTTGAAACTGTGTTACCAGAAAGTGCATGTTCCCA
>JACANZ010202422.1
AATTAGTGCCTTAAAGAGAAACCAGAATACAGTTNNNNNNNNNNNTTGGGTAACTTAATCTACTTGAGTAAG
TTATCAGTTTGCATAAAACACACTGCCCACAGGATCCACAACGTTCCTCCTACATTCTCTGTAAGGAAGAA
GAATGTCAAGTGTCTGCCTTTAAAGTACTTGAATTCCCCGGGCTTTCAGAGGTGTTCTGGGGAAAATGTGTG
CTACCAGCAGTGAATGTGCATTTCGCATTCTAAGAACAAGAAGGTAGATTCTTTCCCGTTCAGCTACAGGA
CGGCAGAAGCGTGGTTGTTTTGCTGACTAAACCTTGTGTTGTAATCACGTGCGTGTTTCTTACCACAAGAAA
GAGAAACCTAAAGACAGTTGTTCTCAGTCTGCTGAAAGAAATGAGAAGGATTCACTTACCATGTATGAAAA
TCCCTCTGTGTTAAGTGTCCAAGAATGCGGCCTATGTATTCTCCTTCTAGGTAAAGAGTTTAACTTTTCTCTC
CTTCGAATAGGTGAAAACCTGCAGGGTTTACAACTTTCCGGGAGGCACTCTATGCTGCCTGGAGGAGAATG

TGGCCTTACAGACAGTATCTCTGAAGGGGAAAAGAAAGCAGGTTTCTTCTGTTCTGGTATACCACAGCAGA
AGAGATGTAGTGTTGCTAAGTAATCCCTATGTTACCAGGACATGCATGTTTCCAC
>JACANZ010316682.1
ATTCACTGCATGAAAGAAAAAGCAAAATACAGAGTTCTCATTCAAGTCGGTTAACTTAATCAATTAATTAA
GCTATCAGTTTTGAGAAAACAGTTTTCTCACACGAGCCACAAACTGTCTCATACATTCTCTGAATGGGAAA
AGAACGTCAAGCTTCTCCCTTTCCAGAACTTGAGTCCCCTGAGTTTTCAAATGTTTCCTAAGGGAACACACG
TTTTGCATTCTACGAAGAAGGCAGATTGTTTCCCGTTCAATGAAAGGACTGCAAAAGCGATCTAATTTTGCT
GACTAGAACTTGTGCTTTAAGGACGTGCGCGTTTCCTATTCACTGGAAGAAAGGGAAACCGAAATACAGTT
TCTCTCCTTCGGGCGGAATCAGTAAGAAAGATTAACTTACTGTGCTTAAAAATCCCTTTGTGTTACACGCAC
CAGAATGTTGCATATACATTCTTTGCCTGGGTAAAGAGTTTATTTTCTCCCCTTGGATTACATGAAGCCATG
CGGGGTTTAAGTGTTTTCAGTAGGCACTCTGTGCTGTCCGGCTAACACTATGCCTCATGGACCGTGCTTTAA
AGGGAGAAAACAATTCGAGTTTCAGCTGTTCAAGTATACGGGAGCAACAGAGATGGCGTTTTGCTAAGTGA
ACATTGCTTTAGCAGAAAGTGCATACTCCCAT
>JACANZ010418236.1
TTTACTGCATGAAAGAGAAAGCAAAATACAGCATTCTCATTCAAGTTGGTTAACTTAATCAAGTGAATGAA
GTAACGAGTTTTGATAAAACTCCTTCCTCACACCGTCCACAAGTTTTCTCAATCTCTTAGTGGGATAAGAAT
GACAAGTCTCTCCCTTTCAAGTACTTGAATCCCTCGGATTTTGAGATGTTTTCTAAGGGCACTCTGCAGTAC
CCAGAATGAAGGTACCTTTCCCATTCTAGGAAGAACAAGGCAGATTGCTTCGCATTCAGCTAAAGGAAGGC
AGAAGCGGTGCAGGTTTGCTGACTAAACTTTGTATTGTAGATGTTTCTAATTCACTTCGAGAAAGAGAAAC
CTAAAGACAGTTACTCTCAGTCTGGTGGATCAAGACAGAAGGATTCACTTACCGTGTTTGAAAAATCCTCT
GTGTTACATGCCCAAGAATATTGCTATGCATTCTCCACCTGGGTAAATATTTTGACTTTTCTCCCGTTAGAG
ACGTGAAGCCCTGCAGAGTTTAGAGGTTTCCAGGAGGCACTTTGTGCTGCCCAGATGAAAATGCAGGCCTA
CAAACGGTATCTCTGAAGGAGAAAACAATGTGCATTTCTCACTTTGACATACAAGACAGAAGTAGGGTTGT
ACTTTTGCGAAGCAAACACTATATTAGCAGGACGTGCATGTTCCCAN
>JACANZ010596973.1
ATTCACCACACGCAAGAGAAAGCAAAATACAGCGTTCTCTTTCAGGCGCTTAACTGTATGTAGTGAAGTTT
TCAGTTTTGTTAGAAGAAACAGTCCGCACACCACAAGCTGCTTTCACAAATGTTCTCAGTGGCATGAGAGT
GCAGAGTGCTGGCCTAGCAAGTATTTGAATCTCCTGCTTTTCAAAGGAATCTTTGCATACCAGGGAATGAA
GGTACTACTGGCTTTCAAAGCAAAACAGGCAGATTTTTTGCCTTTAGCCAGAGGACGGCAGAAGAAGTTCA
GTTTTGCTGACCAGATCTTGTGTGCTTAACACGAGCATGTAGCGTCTTCCCTGCTTGAAAAGGTAAGCAAGA
GCTTGTTGCTCTCAGTCTGGGGATGAAGGGAGAATAATTGACTTTTCCTGTTTGAAAATTCCTCTGTGTTAC
ATACCAAAGAAAGTTGCCTGTGGATTGTCTACATGGATAAAGTGTATGACGTTTCTCTCTTTCAAGTAGTTG
AAGCCCTTCAGGGTTTCCCGCATTCCGTGAGGCATTCTGGGTTGCGCGGCTGAGAATACTGCTTTACAGACG
GTATCGCTAAAGGAGAAGAGAAACCGCGTTTCTCCCTTTCTGGAACGCGAAA
>JACANZ010334892.1
CCCCTTCTGCTATAAGACAGCGGCAGGGCTGTAGTTCTGCTAACTAAACAGTATGTTCATCTAGGTAAATTT
TCAGTTTTGGTAGAACAAACAGTCCTCACACCATCAGCTGCTTTCTCAAACATTCTCTCTATGGGAAAAGAC
TGGGGAGTGTCTGAATTTCAAGTTCTTGAATTACCTAGTTTTCAAAGGGAACTTTGCCTTAAAGGGAATGAA
GGTGACATTTGCATTTTAAGAAAAAAGGGCAGATTGTTTGCCTTCAGCTAGTGGATGGCAGAAGCGATTCA
GTTTTGCTGACTAAAGCTTGTGTGTTTGACCCGAACATGTATCCGATTTCCTGCAGGAAAGAGAAACCCAAT
GACAGTTGCACTCCCTCTGGGGGATGAAGTGAGTAGGATAGACTTACTCTGTTTGAAAATCCCCTGTATTAC
ATGCTCAAGAATGTTGCCTGTGCTTTGTTTACCCGGGTAAAGACTTTGACTTTTCTCTCTTTAGAGTGCGTGA
GGCCCTGCTATGTTTACAGCTTTCCGGGAGGTACTCTGTGCTGTGTGGCCGAGAATGCCGCGTTACAGACGC
TATCCCTGAAGGAGGAGAGAAAGGGCCTTTCTCCCCTTCTGTTACATGGCAGCGGCAGTGCTGTCGTTTTGC
TAACTAAACACTATGTTAGCAACACATATATGTTCCCAA
>JACANZ010280849.1
ATTCACTGAATGAAAGAGAAAGCAAAATNNNNNNNNNNNTCACACCACCAGCTGCTTTCTCAAACTACATCT
CAATGGGATAATACTGGGGAGTGTATGCCTTTCAAGTACTTTAATCCCCTGGTTTTCAAACTTAACTTTGCT
TTACAGGGAATGAAGGTAACATTTGCATTCTATGAAAAACAGGCAGATTGCCTTCAGCTAGAGGACGGCAA
AAACGATTCAGTTTTACTGACTAAACCTTGTGTGGTTGACAAGACCATGTATCCTATTCCTTGCAAGAAAGA
GAAACCAAGTTGCTCTCATACTGGGGAATGAAGTGAGAAGGATTCACTTACTGTGCTTGAAAACCTCTCTG
TTTTCCATGCTGAAGAGAGTTGCGTGTACATTGTCTACCTGGGTAAAGAGTCTGAATTATTTCTCTTCCGAG
TACCTGAAGGCCTGTGGGGTTTACAGCTTTCCGGGAATGATTCTGTGCTGTGCGGCTGAGAATGCCGCCTTA
CAAACGCTATCTCTCAAGGAGAAGACAGAGTGTTTCTCCCGTTCTGGTATACAACAGCAGCAGCGCTGTAG
TTTTCCAGACTAAACACTATGTTAGCAGCAGGGGCAAGATCCAA
